# Alternating styrene–propylene and styrene–ethylene copolymers prepared by photocatalytic decarboxylation†

**DOI:** 10.1039/d3sc03827k

**Published:** 2023-10-02

**Authors:** Emmanuelle Schué, Dillon R. L. Rickertsen, Angie B. Korpusik, Alafate Adili, Daniel Seidel, Brent S. Sumerlin

**Affiliations:** a George & Josephine Butler Polymer Research Laboratory, Center for Macromolecular Science & Engineering, Department of Chemistry, University of Florida Gainesville FL 32611 USA sumerlin@chem.ufl.edu; b Center for Heterocyclic Compounds, Department of Chemistry, University of Florida Gainesville FL 32611 USA

## Abstract

Synthesis of olefin–styrene copolymers with defined architecture is challenging due to the limitations associated with the inherent reactivity ratios for these monomers in radical or metal-catalyzed polymerizations. Herein, we developed a straightforward approach to alternating styrene–propylene and styrene–ethylene copolymers by combining radical polymerizations and powerful post-polymerization modification reactions. We employed reversible addition–fragmentation chain transfer (RAFT) copolymerization between styrene derivatives and saccharin (meth)acrylamide to generate alternating copolymers. Once polymerized, the amide bond of the saccharin monomers was highly reactive toward hydrolysis, an observation exploited to obtain alternating styrene–acrylic acid/methacrylic acid copolymers. Subsequent mild decarboxylation of the (meth)acrylic acid groups in the presence of a photocatalyst and a hydrogen source under visible light resulted in the styrene-*alt*-ethylene/propylene copolymers. Alternating copolymers comprised of either propylene or ethylene units alternating with functional styrene derivatives were also prepared, illustrating the compatibility of this approach for functional polymer synthesis. Finally, the thermal properties of the alternating copolymers were compared to those from statistical copolymer analogs to elucidate the effect of microarchitecture and styrene substituents on the glass transition temperature.

## Abstract 2

We developed a straightforward approach to alternating styrene–propylene and styrene–ethylene copolymers by combining radical polymerizations and powerful post-polymerization modification reactions.

## Graphical abstract

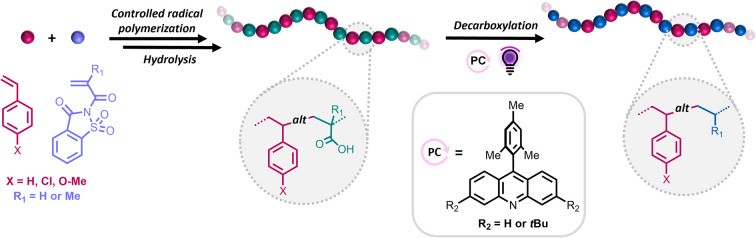

## Introduction

Polyolefins, such as ethylene-based and propylene-based polymers, comprise many common commodity plastic materials. Polyolefins exhibit outstanding chemical resistance and wide-ranging mechanical properties, making them excellent candidates for various applications from disposal containers to ultra-high strength fibers and automobile manufacturing.^1^ However, their simple chemical structure, especially with respect to the lack of functional groups within the polymer backbone, limits their miscibility as homopolymers and impedes their usage in various applications.^2^ Hence, designing polyolefin copolymers has become a significant research focus in the last few decades to expand their utilization into next-generation materials while maintaining their advantageous thermal and mechanical features.^2–6^ Copolymers bearing olefins and polar vinyl monomers, such as acrylates, have already demonstrated enhanced surface properties, dyeability, and miscibility.^7^ Several synthetic approaches based on transition metal-catalyzed α-olefin copolymerization with polar monomers or even polymerization followed by post-polymerization modification methods have led to controlled copolymer compositions and advanced copolymer architectures (mainly random and block structures).^2,8–12^

Another potentially attractive class of olefin copolymers would be those prepared with styrenic comonomers. Polystyrene is typically a brittle polymer with limited ductility and impact strength, while polyolefins in general are ductile thermoplastics with high-impact performance.^13^ Significant research has focused on designing olefin–styrene copolymers to combine their respective features.^14^ Styrene–ethylene random copolymers are particularly attractive due to their impressive viscoelastic performance and thermo-mechanical properties, making them particularly relevant for applications from beauty and personal care containers to foams and compatibilizers.^15–17^ Because they are considerably more deactivated to radical addition, olefins exhibit distinct reactivities compared to styrene, which makes them challenging to copolymerize by conventional methods. For example, styrene polymerizes efficiently *via* conventional radical polymerizations while ethylene requires more extreme conditions (ethylene pressure over 2000 bar and temperature > 200 °C).^18^ Relying on heterogeneous Ziegler–Natta systems for the copolymerization of styrene with ethylene has limitations in terms of copolymer composition, often resulting in homopolymer mixtures or copolymers with low styrene incorporation.^14,19,20^ In the last decades, the development of transition metal-catalyzed polymerizations or “single-site” catalysis has led to copolymers bearing styrene with diverse olefin content and structures.^21^ Nevertheless, reproducibility and control over comonomer sequence remains a significant challenge. It is well-established that, even with an identical comonomer composition, copolymers can display considerably different physical properties depending on their microstructure.^22^ Alternating copolymers have the defined microstructures among synthetic copolymers and often exhibit distinct physical properties compared to their random copolymer counterparts.^23,24^ Recently, pseudo- or ideal alternating copolymerizations of ethylene with styrenic derivatives could be achieved by using advanced scandium-based catalysts.^25,26^ Interestingly, alternating styrene–propylene copolymers remain out of reach using insertion–coordination polymerization methods.^27^

The emergence of reversible-deactivation radical polymerization (RDRP) has opened the path to polymeric materials with tailored structures in terms of composition, topology, functionality, and molecular weight.^28,29^ Concurrently, the last decades witnessed the impressive development of a renewed suite of post-polymerization modification strategies that allow the synthesis of functionalized macromolecules that can not be synthesized directly by polymerization.^30^ The use of mild and highly efficient reactions, especially click reactions,^31^ has proven to be a versatile synthetic strategy to elaborate on-demand functionalized polymer materials, including olefin-based copolymers.^12^ Post-polymerization decarboxylation has become a robust approach to generate alkyl polymers, such as polyolefins. Single electron transfer (SET) on redox-active esters by using either metal complexes or organic photocatalysts has been shown to efficiently induce decarboxylative radical generation directly on the polymer backbone, which has greatly facilitated access to statistical ethylene-based and propylene-based copolymers.^11,32–36^ Interestingly, photochemical decarboxylation using an acridine catalyst was described by Oda in 1991.^37^ This seminal work utilized a stoichiometric amount of acridine in the presence of a hydrogen atom source to synthesize alkanes *via* a proton-coupled electron transfer (PCET) mechanism. Since this pioneering example, Larionov has developed catalytic acridine-catalyzed decarboxylation for various useful transformations, such as the formation of alkenes, sulfonamides, and *N*-alkylations.^38–40^ More recently, our group has relied on this acridine-catalyzed decarboxylation for a straightforward route to access acrylate–olefin copolymers.^41^

**Scheme 1:**
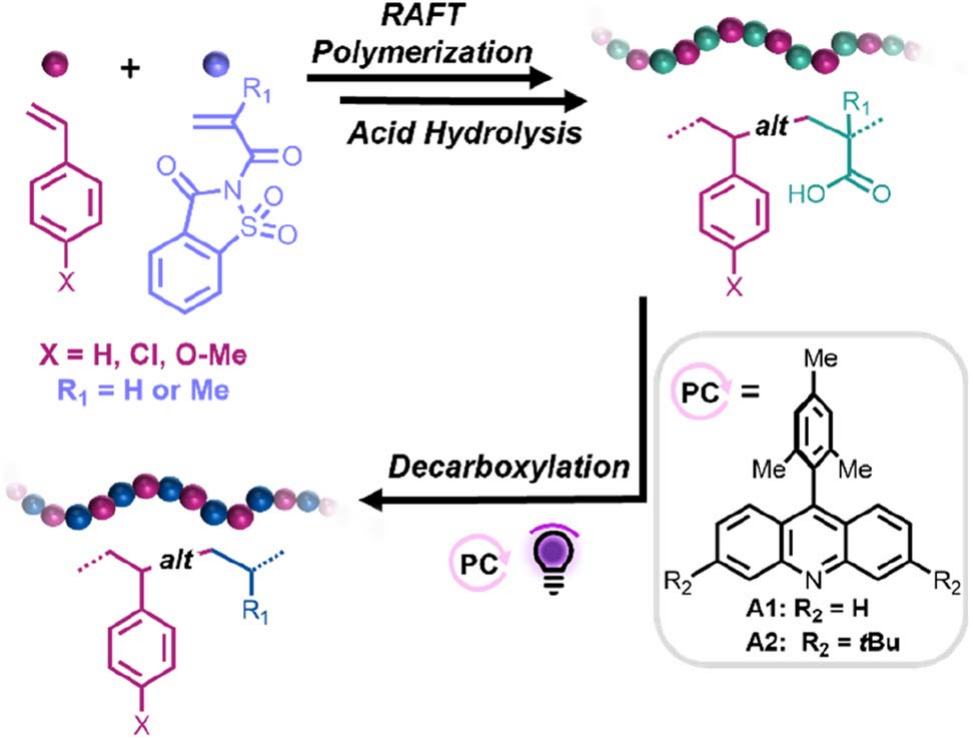
General synthetic approach toward the fabrication of alternating styrene–propylene and styrene–ethylene copolymers.

Herein, we propose a straightforward synthetic strategy to access alternating styrene–ethylene and styrene–propylene copolymers. In this approach, reversible addition–fragmentation chain transfer (RAFT) copolymerizations between styrene derivatives (Sty-X) and saccharin (meth)acrylamide (SacchMA or SacchA) were conducted to generate alternating copolymers. As described in the literature, SacchMA is a bulky and electron-withdrawing *N*,*N*′-disubstituted methacrylamide.^42^ Due to its chemical structure and inherent low reactivity, it does not undergo homopolymerization under general radical polymerization conditions. However, copolymerization of the electron-deficient SacchMA with electron-rich comonomers is accompanied by a two-way high cross-propagation rate to result in highly efficient alternating comonomer incorporation. We reasoned that subsequent mild hydrolysis of the saccharin monomer units would lead to the formation of alternating styrene–acrylic acid/methacrylic acid copolymers and that decarboxylation of the (meth)acrylic acid groups in the presence of an acridine photocatalyst and hydrogen atom source would yield styrene-*alt*-ethylene/propylene copolymers. While perfectly alternating styrene–ethylene copolymers have been prepared by Cui *et al.*,^25^ this would represent the first report, to our knowledge, of alternating styrene–propylene copolymers. The thermal properties of the obtained alternating copolymers could then be compared to their statistical analogs. Such a simple and versatile synthetic strategy offers access to a library of alternating copolymers, also suitable for styrene derivatives bearing polar functional groups, which are usually more difficult to achieve with common coordination–insertion polymerizations (Scheme 1).

## Results and discussion

**Fig. 1:**
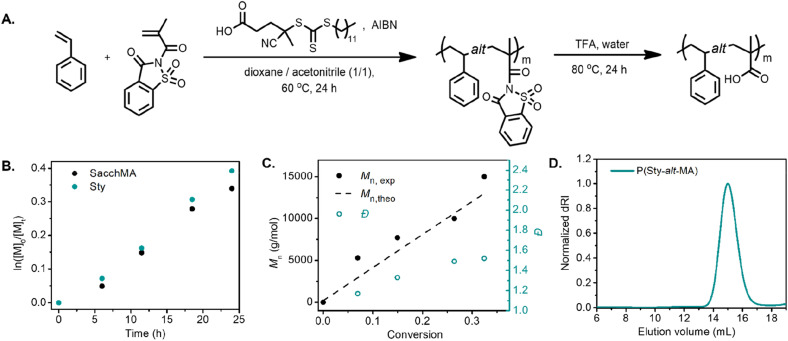
(A) Synthetic scheme of poly(styrene-*alt*-methacrylic acid). (B) Pseudo-first-order kinetic plot of the RAFT copolymerization of styrene and saccharin methacrylamide. (C) Number-average molecular weight *vs.* conversion. (D) SEC analysis of the resulting poly(styrene-*alt*-methacrylic acid) after hydrolysis.

We began our investigation with the copolymerization of styrene (Sty) with saccharin methacrylamide (SacchMA) by RAFT polymerization.^42^ The copolymerization was performed with an equimolar feed of each monomer at 60 °C in dioxane/acetonitrile (1/1 v/v) in the presence of 4-cyano-4-[(dodecylsulfanylthiocarbonyl)sulfanyl]pentanoic acid (CDP) as the chain transfer agent and azobisisobutyronitrile (AIBN) as initiator (Fig. 1A). The polymerization was monitored by ^1^H NMR spectroscopy and size exclusion chromatography (SEC). The pseudo-first-order kinetic plot of the polymerization suggested constant radical concentrations (Fig. 1B). Notably, the comonomers were consumed at the same rate, implying that the alternating sequence was achieved. The molecular weight *vs.* conversion graph evidenced linear evolution consistent with a controlled polymerization (Fig. 1C). The resulting copolymer poly(styrene-*alt*-saccharin methacrylamide) (P(Sty-*alt*-SacchMA)) was isolated and analyzed by SEC and ^1^H NMR spectroscopy. SEC analysis indicated a monomodal peak with relatively narrow dispersity, which further confirmed the polymerization was controlled (Fig. S12,†*M*_n,exp_ = 8400, *Đ* = 1.34). The NMR spectrum established the incorporation of styrene and saccharin methacrylamide monomers in the copolymer chain. Signals corresponding to the saccharin fragment and styrene could be observed at 8.3–7.6 ppm and 7.2–6.7 ppm, respectively (Fig. S28†). It should be noted that the integral intensities obtained from the isolated and purified copolymer did not indicate the equal incorporation of each comonomer that would be expected for an alternating copolymerization. Given that NMR analysis of the aliquots taken during the copolymerization demonstrated equal rates of consumption for each comonomer, we believe this observation may be due to partial hydrolysis of the saccharin amide bond occurring during purification of the final copolymer.^23^

We further investigated the alternating copolymerization process with styrenic derivatives that exhibited different electron densities. 4-Methoxystyrene (StyOMe) and 4-chlorostyrene (StyCl) were copolymerized with SacchMA using the previously described polymerization conditions. In both cases, the styrenic monomers were consumed at the same rate as SacchMA, suggesting the production of the alternating copolymers P(StyOMe-*alt*-SacchMA) and P(StyCl-*alt*-SacchMA), respectively. Interestingly, the pseudo-first-order kinetic plots suggested constant radical concentrations and indicated faster copolymerization rates compared to the unsubstituted styrene. Moreover, the molecular weight *vs.* conversion graph showed a linear evolution of molecular weight with dispersities remaining low (Fig. S7 and S8†). Post-polymerization modification of the copolymers was carried out to obtain a series of alternating styrenic–methacrylic acid copolymers. Although the saccharin amide bond of the monomer is bench stable, the bond becomes more reactive toward hydrolysis or alcoholysis after polymerization,^23^ which makes this monomer unit particularly attractive for post-polymerization functionalization. Indeed, the saccharin methacrylamide monomer units were readily hydrolyzed in trifluoroacetic acid (TFA) at 80 °C to afford alternating poly(styrene-*alt*-methacrylic acid) (P(Sty-*alt*-MA)), poly(4-methoxystyrene-*alt*-methacrylic acid) (P(StyOMe-*alt*-MA)), and poly(4-chlorostyrene-*alt*-methacrylic acid) (P(StyCl-*alt*-MA)). Quantitative hydrolysis was confirmed by ^1^H NMR and ^13^C NMR spectroscopy (Fig. S31–S36†). In the ^1^H NMR spectrum, signals corresponding to the saccharin fragments at approximately 8.3–7.6 ppm disappeared, and the signals attributed to the newly formed carboxylic acid were observed at 12.0–11.8 ppm. The ^13^C NMR spectrum revealed a signal attributed to the sp^2^ carbon of the carboxylic acid at 178 ppm. The SEC analysis, however, demonstrated a clear shift to higher elution volume, indicating a higher hydrodynamic volume (Fig. 1D, *M*_n,exp_ = 19 100, *Đ* = 1.19), despite the mass loss expected for the loss of the pendent saccharin moieties. This initially unexpected observation was attributed to swelling of the polymer after hydrolysis since favorable polymer–solvent interaction *via* hydrogen bonding between carboxylic acid units and DMAc could lead to higher hydrodynamic volumes. Nevertheless, each SEC trace demonstrated a narrow and monomodal polymer distribution (Fig. S13B–S14B†).

We then studied the copolymerization of styrene and styrenic derivatives with saccharin acrylamide (SacchA). First, the monomer was synthesized by reaction of saccharin with acryloyl chloride in the presence of triethylamine. Similarly to its methacrylamide analog, the monomer exhibits limited solubility in most organic solvents and readily decomposes in polar solvents such as DMF and DMSO.^42^ Interestingly, *N*-methyl-2-pyrrolidone (NMP) could solubilize the monomer, and monomer decomposition was negligible in this solvent at ambient temperatures (less than 2% of monomer decomposition at *T* < 50 °C in 5 h). To gain insight into the reactivity of this novel monomer, we first performed the homopolymerization of saccharin acrylamide by conventional radical polymerization. The polymerization was initiated by 2,2′-azobis(4-methoxy-2,4-dimethylvalero-nitrile) (V70) in NMP at 40 °C. The polymerization reached 82% monomer conversion after 30 min. The formation of a polymer was confirmed by SEC analysis (Fig. S1,†*M*_n,exp_ = 14 000, and *Đ* = 2.7). The monomer reactivity was further investigated by copolymerization with styrene by conventional radical polymerization for evidence of potential alternating copolymerization behavior. Polymerizations with different initial comonomer feeds were conducted with the same conditions as the homopolymerizations. The monomer reactivity ratios were calculated by the Fineman–Ross method, resulting in values that were nearly zero: *r*_SacchA_ = 0.053 and *r*_Sty_ = −0.058 (Fig. S2†). Given these results, it is reasonable to assume that the comonomer pair affords an alternating sequence.

**Fig. 2:**
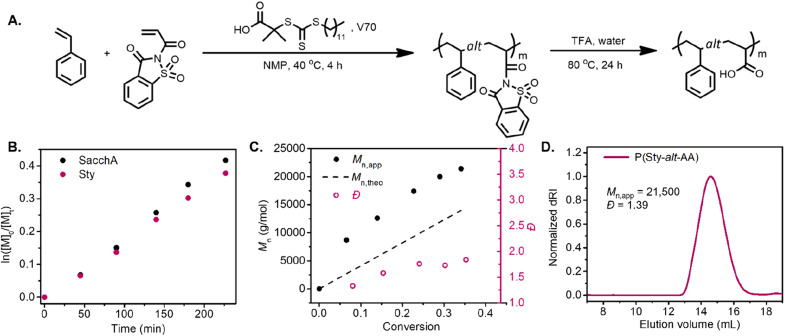
(A) Synthetic scheme of poly(styrene-*alt*-acrylic acid). (B) Pseudo-first-order kinetic plot of the RAFT copolymerization of styrene and saccharin acrylamide. (C) Number-average molecular weight (*M*_n_) *vs.* conversion. (D) SEC analysis of the resulting poly(styrene-*alt*-acrylic acid) after hydrolysis.

RAFT copolymerization of SacchA with styrene was likewise performed to prepare a well-defined alternating copolymer. The copolymerization was conducted with an equimolar feed of each monomer in NMP at 40 °C, in the presence of 2-(dodecylthiocarbonothioylthio)-2-methylpropionic acid (DDMAT) as chain transfer agent and V70 as initiator (Fig. 2A). The comonomers were consumed at identical rates, suggesting that alternating copolymer poly(styrene-*alt*-saccharin acrylamide) was achieved. The pseudo-first-order kinetic plot suggested constant radical concentrations, and the molecular weight *vs.* conversion graphs evidenced a linear evolution consistent with a controlled polymerization (Fig. 2B and C). Again, we further investigated the alternating copolymerization process with the styrenic derivatives Sty–OMe and Sty–Cl, employing the previously described polymerization conditions. In both cases, the styrenic monomers were consumed at the same rate as SacchA, suggesting the production of the alternating copolymers P(StyOMe-*alt*-SacchA) and P(StyCl-*alt*-SacchA), respectively. As previously observed in the copolymerizations with SacchMA, the copolymerizations were controlled and faster with the substituted styrenic derivatives (Fig. S10 and S11†). Surprisingly, SEC analysis indicated a monomodal polymer peak in the cases of P(Sty-*alt*-SacchA) and P(StyCl-*alt*-SacchA) copolymers, while the SEC trace of P(StyOMe-*alt*-SacchA) evidenced broader dispersity with a shoulder in the high molecular weight region of the peak (Fig. S19A,†*M*_n,exp_ = 31 500 and *Đ* = 1.80). To reach narrow molecular weight distributions in RAFT polymerization, the addition rate constant (*k*_add_) of the propagative species to the chain transfer agent must be faster than the propagation rate to encourage only a small amount of monomer addition per chain activation–deactivation cycle.^43,44^ We believe the highly electron-rich character of the methoxy substituent StyOMe and the electron-deficient nature of SacchA lead to considerably higher cross-propagation rate constants than the other styrenic monomers and could lead to reduced polymerization control.

Post-polymerization modification of the copolymers was likewise carried out with TFA to hydrolyze the saccharin acrylamide units into carboxy moieties and successfully obtain the respective alternating poly(styrene-*alt*-acrylic acid) (P(Sty-*alt*-AA)), poly(4-methoxystyrene-*alt*-acrylic acid) (P(StyOMe-*alt*-AA)) and poly(4-chlorostyrene-*alt*-acrylic acid) (P(StyCl-*alt*-AA)). SEC analysis and NMR spectroscopy evidenced clean polymer hydrolysis (Fig. 2D and S40–S45†).

Statistical styrene–methacrylic acid and styrene–acrylic acid copolymer analogs were also synthesized to allow evaluation of any sequence effects on the thermal properties of the targeted styrene–olefin copolymers. The statistical copolymers were prepared by RAFT copolymerizations of styrene derivatives with *tert*-butyl methacrylate or *tert*-butyl acrylate, followed by hydrolysis in acidic conditions to yield the statistical styrene–methacrylic acid (P(StyX_mol%_-*stat*-MA_mol%_)) and styrene–methacrylic acid copolymers (P(StyX_mol%_-*stat*-AA_mol%_)) respectively, with X = H, O–Me, Cl. Due to the difference in monomer reactivities, the comonomer feed for each copolymerization was adjusted to achieve nearly equal monomer incorporation as in the alternating copolymers, and the polymerizations were stopped at <10% conversion to prevent comonomer feed drift. After hydrolysis, the three copolymers had similar molecular weights and similar styrene–(meth)acrylic acid compositional ratios (*i.e.*, 50/50), as determined by ^1^H NMR spectroscopy (Fig. S46–S63†).

The resulting alternating and statistical copolymers were then subjected to a proton-coupled electron transfer (PCET) process *via* acridine-catalyzed decarboxylation. In the presence of phenylthiol as a hydrogen donor, the carboxylic acid groups on the acrylic or methacrylic acid units should be converted to ethylene or propylene units, respectively. This strategy was employed to decarboxylate the (meth)acrylic acid units to yield alternating styrene–propylene or styrene–ethylene copolymers. The proposed mechanism for this process involves the formation of a photoactive hydrogen-bonded complex of the (meth)acrylic acid and the acridine catalyst, initiating PCET under purple light irradiation, followed by *in situ* decarboxylation. Hydrogen-atom abstraction from a thiol by the resulting secondary or tertiary backbone-centered radical yields the alternating olefin copolymer, and the reduced acridine reacts with the resulting thiyl radical to regenerate the photocatalyst.

**Fig. 3:**
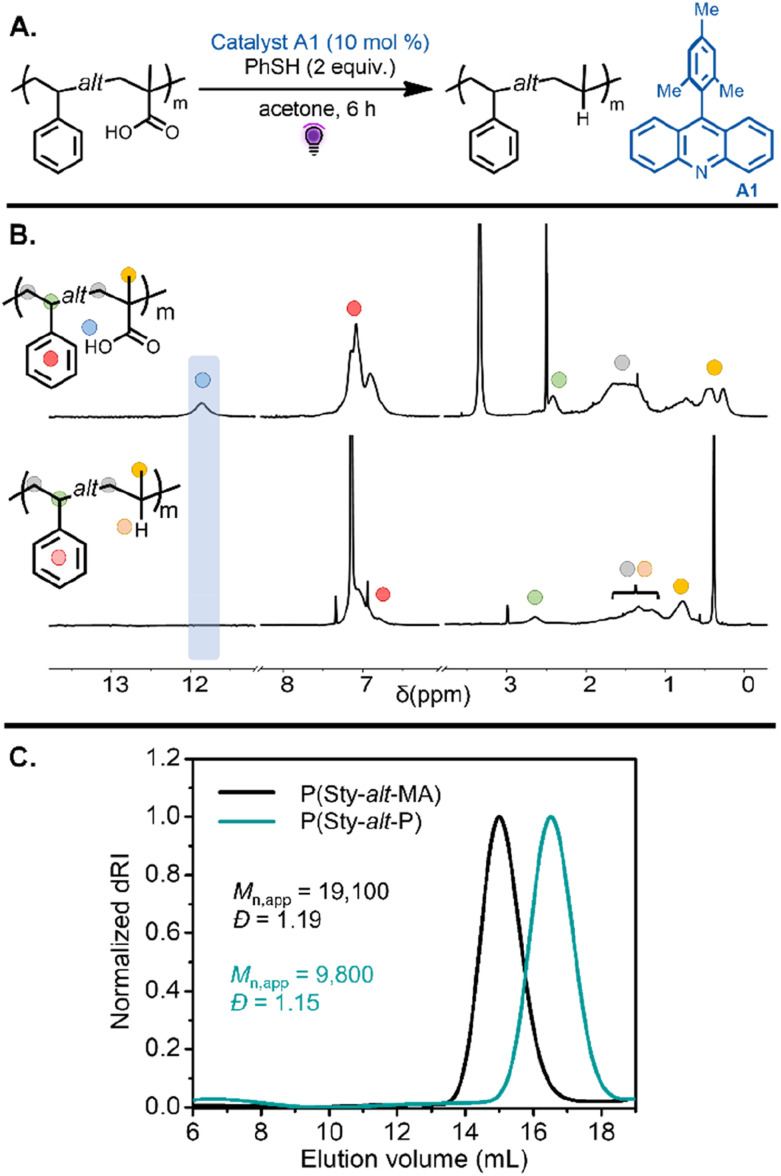
(A) Decarboxylation of P(Sty-*alt*-MA) was performed with A1 under purple light irradiation, leading to an alternating styrene–propylene copolymer. (B) ^1^H NMR spectra of P(Sty-*alt*-MA) in DMSO-d_6_ and the resulting P(Sty-*alt*-P) in benzene-d_6_ after decarboxylation, showing the disappearance of the methacrylic acid proton and a shift of the methyl signal. (C) SEC chromatograms of P(Sty-*alt*-MA) and the resulting P(Sty-*alt*-P) after decarboxylation indicated a decrease in molecular weight of the copolymer.

Investigation into this decarboxylation approach began with the alternating copolymers bearing methacrylic acid units. Inspired by our previous work,^41^ the reaction was first tested on P(Sty-*alt*-MA) by using 10 mol% of 9-mesitylacridine (A1) with respect to carboxylic acid groups in the polymer chain and 2 equiv. thiophenol as a hydrogen atom source (Table 1, entry 1). The reaction was carried out in acetone under purple light for 6 h (Fig. 3A). The resulting poly(styrene-*alt*-propylene) P(Sty-*alt*-P) copolymer was isolated and analyzed by ^1^H and ^13^C NMR spectroscopy and SEC. The ^1^H NMR spectrum showed the complete disappearance of the carboxylic acid proton signal, suggesting the reaction was quantitative (Fig. 3B). Moreover, the methyl proton signals at 0.5–0.25 ppm shifted downfield to 0.8 ppm, consistent with the methyl pendant of a propylene repeat unit. The ^13^C NMR spectrum confirmed quantitative decarboxylation, showing a disappearance of the carbonyl signal at 178 ppm (Fig. S64†). The SEC traces demonstrated a clean shift toward lower molecular weight, as expected for a reduction in molecular weight due to the release of CO_2_ upon decarboxylation (Fig. 3C). It should be mentioned that the decrease in apparent molecular weight observed in the elugram was potentially amplified by a reduction in the hydrodynamic volume of the copolymer due to the conversion of methacrylic acid units into propylene units that are expected to swell less in the eluent.^41,45^

**Table 1:** Library of alternating and statistical styrene–propylene and styrene–ethylene copolymers prepared by RAFT copolymerizations of the corresponding saccharin (meth)-acrylamide followed by hydrolysis and photocatalytic decarboxylation

Entry	Copolymer	Copolymer composition^a^ StyX/P or E (mol%)	Catalyst	*M*_n,app_ b (g mol^−1^)	*Đ* b	*T*_g_ c (°C)	*T*_g_ breadth^c^ (°C)
1	P(Sty-*alt*-P)	50/50	A1	9800	1.15	71 ± 1	17
2	P(Sty-*stat*-P)	54/46	A1	12 000	1.39	63 ± 1	20
3	P(StyOMe-*alt*-P)	51/49	A1	12 300	1.21	70 ± 1	14
4	P(StyOMe-*stat*-P)	51/49	A1	8000	1.23	64 ± 1	17
5	P(StyCl-*alt*-P)	50/50	A1	10 600	1.32	110 ± 1	19
6	P(StyCl-*stat*-P)	47/53	A1	14 500	1.37	80 ± 0	20
7	P(Sty-*alt*-E)	50/50	A2	9700	1.37	26 ± 1	13
8	P(Sty-*stat*-E)	47/53	A2	14 800	1.30	24 ± 0	16
9	P(StyOMe-*alt*-E)	50/50	A2	9600	1.69	39 ± 2	17
10	P(StyOMe-*stat*-E)	54/46	A2	10 500	1.19	46 ± 0	20
11	P(StyCl-*alt*-E)	50/50	A2	11 000	1.22	47 ± 1	18
12	P(StyCl-*stat*-E)	49/51	A2	8800	1.34	56 ± 0	21

a Copolymer composition was determined by ^1^H NMR spectroscopy of the copolymer before decarboxylation.

b Apparent molecular weights and dispersities were estimated by SEC in *N*,*N*-dimethylacetamide with a polystyrene calibration.

c Glass transition temperatures and breadths were determined by differential scanning calorimetry (DSC).

Following the success of the decarboxylation reaction on the alternating styrene–methacrylic copolymer, we similarly sought to decarboxylate MA units on our alternating and statistical copolymer library to obtain a series of poly(styrene-*co*-propylene) copolymers (Table 1, entries 2–6). Similar NMR spectroscopy and SEC analysis were conducted, again revealing quantitative conversion of MA units into propylene units (Fig. S65–S74†) and clean shifts toward lower molecular weight in the SEC traces (Fig. S13–S17B†).

**Fig. 4:**
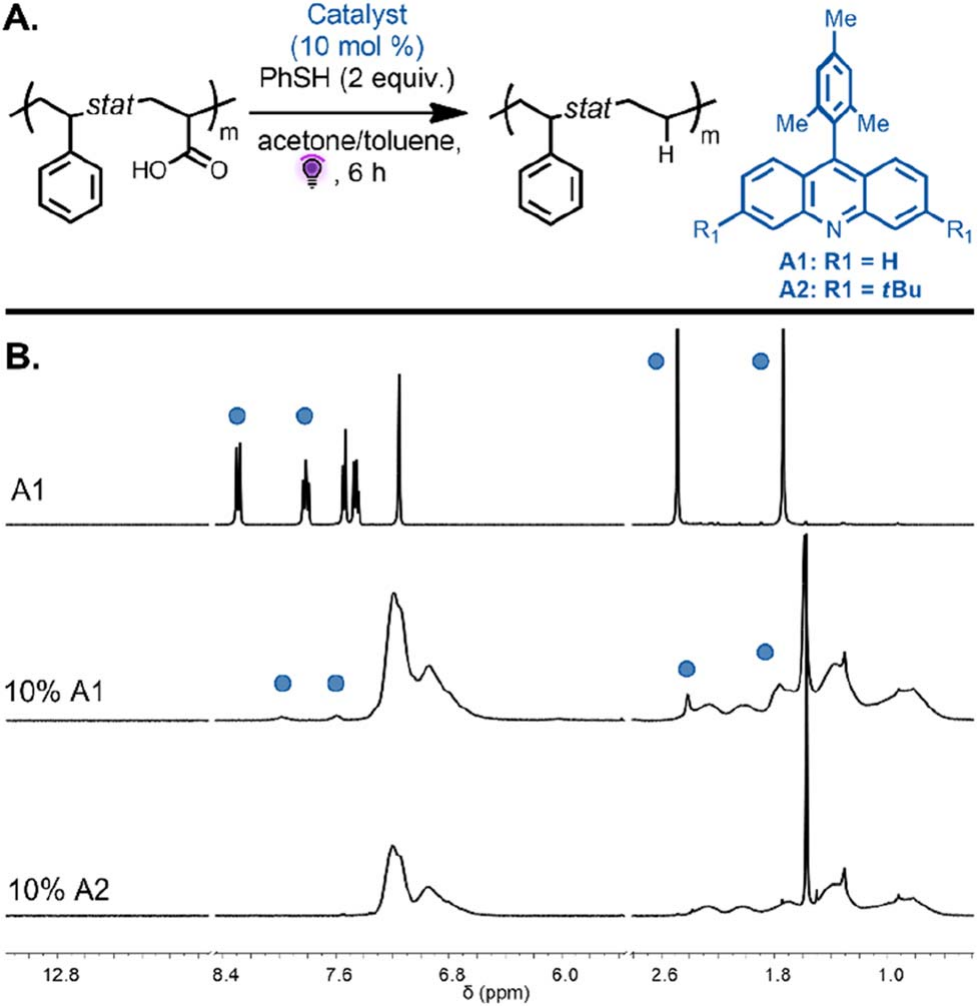
(A) Decarboxylation of P(Sty_56_-*stat*-AA_44_) performed with A1 and A2 under purple light irradiation, leading to an alternating styrene–propylene copolymer. (B) ^1^H NMR spectrum of photocatalyst A1 (top), the P(Sty_0.56_-*stat*-E_0.44_) product obtained with 10 mol% of photocatalyst A1 showing photocatalyst addition to the backbone (middle), and the P(Sty_0.56_-*stat*-E_0.44_) product obtained with 10 mol% of photocatalyst A2 showing no catalyst addition to the polymer backbone. All spectra recorded in (CD_2_Cl_2_). The blue circles are referring to the signals corresponding to the catalyst A1 added on the copolymer backbone.

We next focused on the decarboxylation of AA units on both alternating and statistical copolymers. The statistical copolymer P(Sty_0.56_-*stat*-AA_0.44_) was decarboxylated with 10 mol% of A1 catalyst and thiophenol (2 equiv.) in acetone under purple light. The polymer became insoluble over the course of the reaction due to the decrease in polymer solubility during the reaction as the AA units were converted to ethylene units and potential intermolecular backbone-radical recombination. The poor solubility potentially induced inter-chain association that impeded hydrogen-atom abstraction from thiophenol to favor intermolecular radical recombination. When the same reaction was performed in a solvent mixture of acetone/toluene (2/1 v/v), the copolymer remained in solution during the reaction and was then purified by precipitation leading to the decarboxylated polymer P(Sty_0.56_-*stat*-E_0.44_) (Fig. 4A). SEC analysis revealed a monomodal peak, strongly suggesting the absence of intermolecular coupling (Fig. S3,†*M*_n,exp_ = 5300 and *Đ* = 1.36). While the ^1^H NMR spectra of the isolated copolymers showed complete disappearance of the signal corresponding to the carboxylic acid proton, they also evidenced unexpected signals at 7.95 and 7.54 ppm (Fig. 4B). These proton resonances can be attributed to the photocatalyst aromatic protons of the product that result from the *in situ* generated backbone radicals on the polymer undergoing recombination with the radical present on the catalyst before being capped by the proton donor.^37^ This reaction was potentially avoided during the analogous decarboxylation of MA units due to the steric hindrance of the methyl group on the polymer backbone. The spectra suggest that approximately 45% of the loaded catalyst was incorporated into the polymer backbone. To inhibit this undesired side reaction, a catalyst bearing *tert*-butyl substituent groups (3,6-di-*tert*-butyl-9-mesitylacridine A2, 10 mol%) was employed to investigate the decarboxylation reaction on the same copolymer, P(Sty_0_._56_-*stat*-AA_0_._44_). We reasoned that bulky substituents would limit the extent of the undesirable recombination reaction. Excitingly, ^1^H NMR spectroscopy of the purified copolymer revealed complete disappearance of the carboxylic acid protons, and the proton resonances of the catalyst were no longer detected. The signals at 1.8–1.2 ppm, corresponding to the methylene protons, shifted downfield to approximately 1.1–0.9 ppm (Fig. 4B, bottom spectra). Moreover, SEC analysis indicated a monomodal peak distribution and a decrease in apparent molecular weight due to the removal of the carboxylic acid groups (Fig. S2,†*M*_n,exp_ = 6200 and *Đ* = 1.27). These results are consistent with quantitative decarboxylation and inhibition of the previously observed side reaction that resulted in catalyst incorporation.

After optimizing the decarboxylation reaction on the statistical styrene–acrylic acid copolymer, we proceeded to apply the reaction to a library of alternating and statistical poly(styrene-*co*-ethylene) copolymers (Table 1, entries 7–12). In all cases, quantitative conversion of the carboxylic acid of the AA units into E units was observed (Fig. S75–S86†). SEC chromatograms again showed clean shifts toward lower molecular weight, confirming a reduction of the hydrodynamic volume of the copolymer upon loss of CO_2_ (Fig. S18–S23B†). Moreover, alternating styrene–ethylene copolymer microstructure is accurately determined by ^13^C NMR spectroscopy.^21^ To further confirm the alternating feature of our copolymers, we carefully analyzed the obtained ^13^C NMR spectrum of P(Sty-*alt*-E) (Fig. S76†). We found that the methylene signals at 37.0, 36.5, and 25.5 ppm and the benzylic signal at 45.5 ppm match reported assignments and are characteristic of an alternating sequence.

After synthesizing the entire copolymer library, we decided to further investigate the efficiency of the decarboxylation reactions triggered by A1 and A2 on styrenic copolymers containing MA and AA units, respectively. Our goal was to determine the minimum catalyst loading required for complete conversion of MA and AA units into propylene and ethylene units, respectively, and evaluate the efficiency of both catalysts. We tested catalyst A1 on the statistical copolymer P(Sty_0.45_-*stat*-MA_0.55_) with 2.5, 8, and 10 mol% loading with respect to the MA units. In parallel, catalyst A2 was applied to the statistical copolymer P(Sty_0.56_-*stat*-AA_0.44_) with 2.5, 8, and 10 mol% loading in relation to the AA units. We observed quantitative and clean decarboxylation in all cases, even with the lowest catalyst loadings (see Fig. S5 and S6†). These results highlight the versatility of post-polymerization modification to access functional copolymers that are difficult to obtain using current polymerization methods. Finally, we investigated the series of styrene–propylene and styrene–ethylene copolymers to elucidate the effects of copolymer microstructure on thermal properties. The glass transition temperatures (*T*_g_) of the obtained statistical and alternating copolymers were determined by differential scanning calorimetry (DSC) (Fig. S87–S92A†). The breadth of the glass transition was evaluated using the peak onset and offset in the derivative of the heat flow curve (Fig. S87–S92B and C†). To compare the *T*_g_ of alternating and statistical copolymers, we carefully selected statistical copolymers exhibiting monomer ratios close to 1 : 1 to minimize any composition effects. It should be mentioned that in some cases the dispersity of the alternating and statistical copolymers differ, but we believe the molecular-weight distribution has very little influence on the glass transition temperature.^46^

As shown in Table 1, both the value of *T*_g_ and the breadth of the transition depended on the copolymer microstructure and sequence. Notably, the glass transitions of the statistical copolymers tended to be slightly broader. This increase in breadth is attributed to the more random and inhomogeneous sequence of the statistical copolymers (homodiads, homotriads, *etc.*), leading to a more diverse array of microenvironments than is present in the alternating copolymers.^24^ As expected, the *T*_g_ values of the styrene–propylene copolymers were higher than those of the styrene–ethylene copolymers. In each copolymer library, 4-chlorostyrene-based copolymers demonstrated the highest *T*_g_, while Sty–OMe and unsubstituted styrene Sty–H demonstrated similar transition temperatures.

## Conclusions

These results represent a straightforward method for preparing alternating styrene–propylene and styrene–ethylene copolymers by leveraging controlled radical polymerization and powerful post-polymerization modification reactions. Alternating copolymers were generated by conducting RAFT copolymerization between styrene and the electron-deficient saccharin methacrylamide or acrylamide. After hydrolysis of the saccharin monomer, we obtained alternating styrene–methacrylic acid and styrene–acrylic acid copolymers. The desired styrene-*alt*-ethylene/propylene copolymers were obtained through mild and direct decarboxylation under visible light in the presence of a photocatalyst and a hydrogen source. The synthetic approach also enabled the creation of olefin copolymers with functional styrene derivatives that are difficult to obtain using conventional coordination–insertion polymerizations. Furthermore, due to architecture and styrene substituent effects, significant differences in glass transition temperatures and breadths were found between the thermal properties of the obtained alternating copolymers and their statistical analogs. Overall, this study demonstrates the versatility of the presented method for the preparation of functional copolymers and expands the range of possible synthetic routes for obtaining these materials.

## Author contributions

Conceptualization, E. S., D. R. L. R., A. B. K., A. A., D. S., B. S. S.; methodology, E. S., D. R. L. R., A. B. K., A. A., D. S., B. S. S.; validation, E. S., D. R. L. R., A. B. K., A. A., D. S., B. S. S.; investigation, E. S., D. R. L. R., A. B. K., A. A.; data curation, E. S., D. R. L. R.; writing, E. S., D. R. L. R., A. B. K., A. A., D. S., B. S. S.; supervision, D. S., B. S. S.; project administration, D. S., B. S. S.; funding acquisition, B. S. S., D. S.

## Conflicts of interest

There are no conflicts to declare.

## Supplementary Material

SC-014-D3SC03827K-s001
