# Supplementary material for: Alternating styrene–propylene and styrene–ethylene copolymers prepared by photocatalytic decarboxylation
Source: Chem Sci. 2023 Oct 2;14(40):11228–36. doi: 10.1039/d3sc03827k (PMC10583696; doi:10.1039/d3sc03827k)
Supplement: SC-014-D3SC03827K-s001 [file SC-014-D3SC03827K-s001.pdf]

Electronic Supplementary Material (ESI) for Chemical Science.
This journal is © The Royal Society of Chemistry 2023

Alternating Styrene-Ethylene and Styrene-Propylene Copolymers Prepared by Photocatalytic Decarboxylation

Emmanuelle Schué, Dillon R. L. Rickertsen, Angie B. Korpusik, Alafate Adili, Daniel Seidel,* Brent S. Sumerlin*

George & Josephine Butler Polymer Research Laboratory, Center for Macromolecular Science & Engineering, Department of Chemistry, University of Florida Gainesville, FL 32611 (USA)

Center for Heterocyclic Compounds, Department of Chemistry, University of Florida Gainesville, FL 32611 (USA)

1. Materials and Instrumentation.. 2
2. Experimental Procedures 4
2.1 Monomer synthesis 4
2.2 Photocatalyst synthesis 4
2.3 General synthetic procedures for alternating P(StyX-*alt*-P) copolymers 5
2.3 General synthetic procedures for alternating P(StyX-*alt*-E) copolymers 7
2.4 General synthetic procedures for statistical P(StyX-*stat*-P) copolymers 8
2.5 General synthetic procedures for statistical P(StyX-*stat*-E) copolymers 10
2.6 Free radical polymerization of SacchA 12
2.7 Determination of monomer reactivity ratio by the Fineman-Ross method 13
2.8 Photocatalyst studies 14
3. Characterization.. 18
3.1 RAFT Polymerization Data 18
3.2 SEC Analysis 20
3.3 NMR characterizations 27
3.4 DSC thermograms 59
4. References 62

1. Materials and Instrumentation

Materials

Saccharin (Sacch, Sigma-Aldrich), triethylamine (Et_3N, Sigma-Aldrich), acryloyl chloride (Sigma-Aldrich), methacryloyl chloride (Sigma-Aldrich), *N*-Methyl-2-pyrrolidone (NMP, extra dry over molecular sieve, Thermofisher), trifluoroacetic acid (TFA, Sigma-Aldrich), acetonitrile (ACN, extra dry over molecular sieve, Thermofisher), thiophenol (PhSH, Acros Organics) were used as received unless otherwise stated.

Styrene (Acros Organics), 4-methoxystyrene (Sty-OMe, Sigma-Aldrich), 4-chlorostyrene (TCI, *tert*-butyl methacrylate (*t*BuMA, Acros Organics), *tert*-butyl acrylate (*t*BuA, Acros Organics), 4-methoxystyrene (Sty-OMe, Sigma-Aldrich) were filtered through basic alumina before use. Dioxane (TCI) was dried over molecular sieves (4Å).

Azobisisobutyronitrile (AIBN) and 2,2'-Azobis(4-methoxy-2,4-dimethylvaleronitrile) (V-70, Fujifilm Wako Chemical Laboratories) were recrystallized in methanol prior to use. 2-(Dodecylthiocarbonothioylthio)-2-methylpropionic acid (DDMAT)[1] and 4-cyano-4-[(dodecyl-sulfanylthiocarbonyl)sulfanyl] pentanoic acid (CDP)[2] were synthesized as previously reported.

Instrumentation

NMR Spectroscopy

^1H NMR and ^{13}C NMR spectroscopy were recorded on a Bruker 400 MHz NMR spectrometer and a Bruker 600 MHz NMR spectrometer. Deuterated chloroform ($CDCl_3$), deuterated dichloromethane (CD_2Cl_2), deuterated benzene (benzene-d_6), and deuterated dimethyl sulfoxide (DMSO-d_6) were used as the solvent, and the residual solvent signal served as a reference.

Size Exclusion Chromatography (SEC)

SEC was performed in *N,N*-dimethylacetamide (DMAc) with 50 mM LiCl at 50 ˚C and a flow rate of 1.0 mL/min. The system utilized an Agilent isocratic pump, degasser, and autosampler. The columns were a Viscogel I-series 5 µm guard column and two ViscoGel I-series G3078 mixed bed columns with molecular weight range $0-20 \times 10^3$ and $0-100 \times 10^5$ g/mol). Detection consisted of a Wyatt Optilab T-rEX refractive index detector operating at 658 nm and a Wyatt miniDAWN Treos light scattering detector operating at 659 nm. Absolute molecular weights and molecular weight distributions were estimated with a polystyrene calibration.

Differential Scanning Calorimetry (DSC)

DSC experiments were performed on a TA instruments DSC 2500 (TA Instruments, New Castle, DE), equipped with a Fusion Cell, T4P Tzero heat flow technology, and an RSC 90 two-stage refrigerated cooling system (TA Instruments, New Castle, DE). Samples were sealed in TA Instruments TZero pans for heat capacity measurements. Ramp experiments were heated under nitrogen (50 mL/min) at 10 ˚C/min from -30 to 200 ˚C and cooled from 200 to -30 ˚C, with 5-min isotherms at each extreme (3 cycles). All DSC experiments were recorded using the Thermal Advantage for Q Series software from TA.

Photoreactor

Decarboxylations were performed using a Penn PhD Photoreactor M2 with a 395 nm LED. The light source was operated at an intensity level of 85%.

Melting point

Melting points were recorded on an Electrothermal Digital Mel-Temp 3.0 melting point apparatus.

High-resolution mass spectra (HRMS)

High-resolution mass spectra were obtained from an Agilent 6230 ESI-TOF instrument.

2. Experimental Procedures

2.1 Monomer synthesis

Saccharin methacrylamide was synthesized as previously reported.[3]

Synthesis of saccharin acrylamide (SacchA)

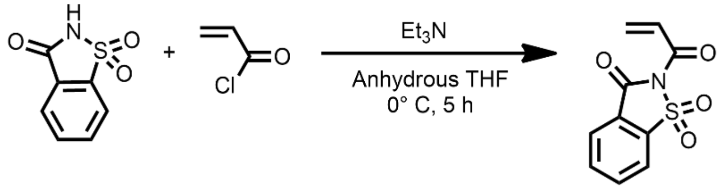

In a 250-mL flask, saccharin (1.0 equiv, 38 mmol, 7.0 g) was dissolved in 100 mL of anhydrous THF under inert atmosphere. Triethylamine (1.05 equiv, 40.0 mmol, 5.57 mL) was added, and the mixture was cooled to 0 ˚C. Acryloyl chloride (1.05 equiv, 40.0 mmol, 3.26 mL) was added dropwise, and the reaction was stirred at room temperature for 5 h. The residue was concentrated under reduced pressure and redissolved in dichloromethane. The organic phase was washed with water (×3), brine (×3) and dried over magnesium sulfate. The crude product was concentrated in a small amount of dichloromethane and recrystallized to afford a white solid (4.5 g, 50%).

^{1}H NMR (400 MHz, $CDCl_3$): δ 8.18 (d, *J* = 7.6 Hz, 1H), 8.08 – 7.83 (m, 3H), 7.34 (dd, *J* = 16.9, 10.5 Hz, 1H), 6.80 (dd, *J* = 16.9, 1.3 Hz, 1H), 6.12 (dd, *J* = 10.5, 1.3 Hz, 1H).

^{13}C NMR (101 MHz, $CDCl_3$) δ 162.9, 157.6, 138.4, 136.5, 135.3, 134.9, 127.8, 126.4, 125.1, 121.3.

2.2 Photocatalyst synthesis

The unsubstituted acridine photocatalyst A1 was synthesized as previously reported.[4, 5]

3,6-Di-tert-butyl-9-mesitylacridine (A2)

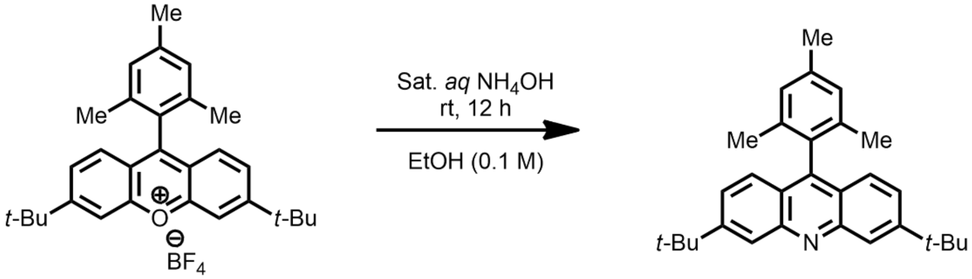

3,6-Di-*tert*-butyl-9-mesitylxanthylium tetrafluoroborate[6] (1.25 g, 2.50 mmol, 1.00 equiv) was dissolved in ethanol (0.1 M) and stirred until complete dissolution. Following complete dissolution, conc. NH_4OH solution (8.4 mL) was added, and the reaction mixture was left to stir at room temperature for 12 h. The ethanol was removed under reduced pressure. Water (35 mL) was added and extracted with diethyl ether (3 × 30 mL). The combined organic layers were washed with brine (35 mL) and dried over Na_2SO_4. The solvent was removed under reduced pressure, and the crude residue was purified by silica gel chromatography using hexanes containing ethyl acetate (0–2.5%) followed by recrystallization in hot hexanes, yielding a white solid in 65% yield (668 mg).

R_f = 0.28 in hexanes/EtOAc (95:5 v/v).

Mp = 243 – 246 °C

^{1}H NMR (600 MHz, $CDCl_3$): δ = 8.19 (s, 2H), 7.47–7.44 (comp, 2H), 7.43–7.39 (comp, 2H), 7.07 (s, 2H), 2.44 (s, 3H), 1.72 (s, 6H), 1.45 (s, 18H).

^{13}C NMR (150 MHz, $CDCl_3$): δ = 152.9, 150.2, 145.6, 137.7, 136.7, 132.2, 128.2, 125.5, 124.9, 124.2, 123.2, 35.2, 31.8, 21.2, 19.5.

HRMS (ESI-TOF): *m/z* calcd for $C_{30}H_{35}N$ $[M + H]^+$: 410.2842; found: 410.2852.

2.3 General synthetic procedures for alternating P(StyX-*alt*-P) copolymers

RAFT copolymerization of styrene (derivatives) and saccharin methacrylamide (SacchMA)[7]

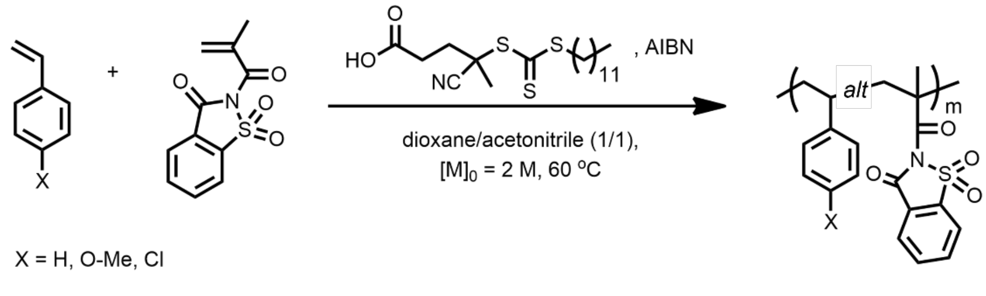

Styrene (100 equiv, 8.20 mmol, 1.42 mL) was dissolved in dry dioxane/acetonitrile (1/1 v/v, 11.5 mL) and was sparged with argon. SacchAM (100 equiv, 12.4 mmol, 3.11 g), CDP (1.00 equiv, 0.124 mmol, 0.0500 mg), and trioxane (50 mg, internal standard) were added to the Schlenk flask under an inert atmosphere. The solution was sparged with argon again, and AIBN (0.200 equiv, 4.07 mg, 0.0250 mmol) was added. The flask was then immersed in a preheated oil bath at 60 ˚C. The polymerization was monitored by taking aliquots at regular time intervals by syringe under argon. The reaction was quenched by opening the flask to air and cooling it in an ice bath. Monomer conversions were calculated by ^{1}H NMR spectroscopy in $CDCl_3$ using trioxane as an internal standard. The final polymer was dialyzed against acetone before removing the solvent under reduced pressure to yield the pure polymer.

P(StyOMe-*alt*-SacchMA) and P(StyCl-*alt*-SacchMA) were prepared with the same procedure: [StyX; SacchMA; CDP; AIBN] = [100;100;1;0.2] in dioxane/acetonitrile (1/1) at 60 ˚C.

Hydrolysis of saccharin pendent units into methacrylic acids[3]

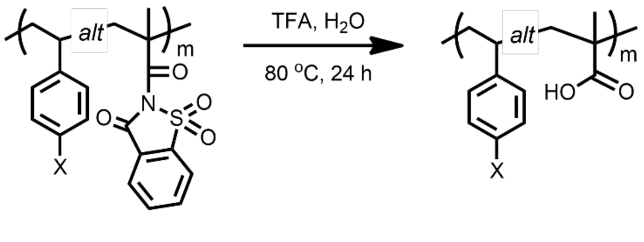

X = H, O-Me, Cl

The alternating copolymer P(Sty-*alt*-SacchMA) (0.705 g) was suspended in the solvent mixture solvent of TFA/H_2O (14/1 v/v, 15 mL). The mixture was stirred for 24 h at 80 ˚C. The reaction mixture was poured into Et_2O to remove TFA. The precipitated mixture of polymer and saccharin was purified by dialysis against acetone to obtain the alternating copolymer P(Sty-*alt*-MA) as slightly brown solid.

The same experimental procedure was used for the hydrolysis of the alternating copolymers P(StyOMe-*alt*-SacchMA) and P(StyCl-*alt*-SacchMA) to yield, respectively, P(StyOMe-*alt*-MA) and P(StyCl-*alt*-MA).

Direct decarboxylation of methacrylic acid units[8]

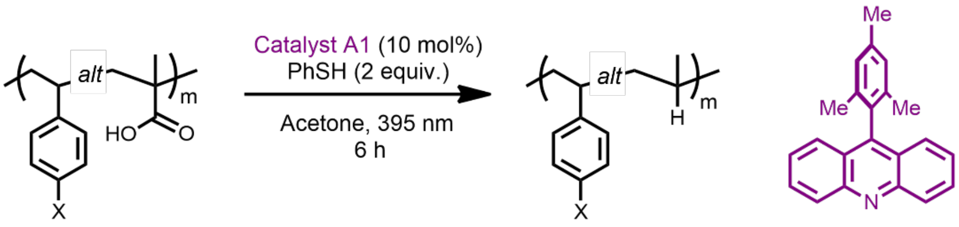

X = H, O-Me, Cl

To a flame-dried vial charged with a stir bar was added P(Sty-*alt*-MA) (76.1 mg) and acridine photocatalyst A1 (12 mg, 0.040 mmol, 10 mol% with respect to MA units). Acetone (3 mL) was added, and the solution was stirred until dissolution. The vial was then purged by bubbling nitrogen through the reaction mixture for 5 min. Thiophenol (2.0 equiv, 0.80 mmol, 82 μL) was added, and the reaction mixture was irradiated with 395 nm light at room temperature for 6 h. The solvent was removed, and the polymer was purified by precipitation in methanol (×3) to yield the pure P(Sty-*alt*-P).

P(StyOMe-*alt*-P) and P(StyCl-*alt*-P) were synthesized by using the same experimental reaction conditions using P(StyOMe-*alt*-MA) (88.1 mg) and P(StyCl-*alt*-MA) (89.9 mg).

2.3 General synthetic procedures for alternating P(StyX-*alt*-E) copolymers

RAFT copolymerization of styrene (derivatives) and saccharin acrylamide (SacchA)

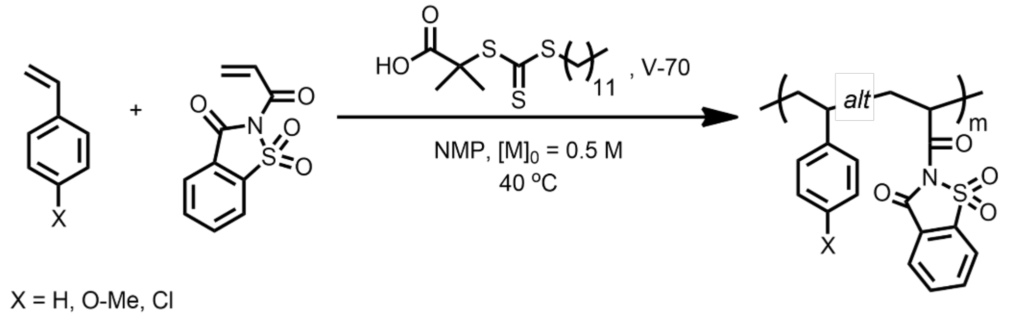

SacchA (120 equiv, 8.24 mmol, 1.95 g), styrene (120 equiv, 8.24 mmol, 0.950 mL), DDMAT (1.0 equiv, 25 mg, 0.070 mmol), trioxane (44 mg, internal standard) were added to a dried Schlenk flask and dissolved in dry NMP (31 mL) under inert atmosphere. The solution was sparged with argon, and *V-70* (0.100 equiv, 7.00 µmol, 2.10 mg) was added under inert atmosphere. The flask was then immersed in a preheated oil bath at 40 ˚C. The polymerization was monitored by taking aliquots at regular intervals by syringe under argon. The reaction was quenched by opening the flask to air and cooling it in an ice bath. Monomer conversions were calculated by ^{1}H NMR spectroscopy in CD_2Cl_2 using trioxane as an internal standard. The final polymer was dialyzed against acetone before removing the solvent under reduced pressure to afford the pure polymer P(Sty-*alt*-SacchA).

P(StyOMe-*alt*-SacchA) and P(StyCl-*alt*-SacchA) were prepared with the same procedure: [StyX; SacchA; DDMAT; *V70*] = [120;120;1;0.1] in dioxane/acetonitrile (1/1, [M]$_0$ = 0.5 M) at 40 ˚C.

Hydrolysis of saccharin pendant units into acrylic acids

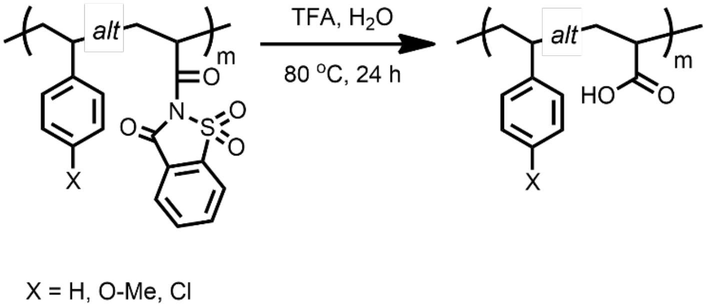

The alternating copolymer (0.60 g) was dissolved in trifluoroacetic acid (12 mL) and water (1.0 mL) in a 25-mL round-bottom flask. The reaction flask was then heated in an oil bath at 80 ˚C for 24 h. The resulting brown solution was poured into cold diethyl ether, and the precipitate was isolated by centrifugation. The solid was dissolved in acetone and purified by dialysis against acetone before removing the solvent under reduced pressure to yield the pure polymer (white solid).

The same experimental procedure was used for the hydrolysis of the alternating copolymers P(StyOMe-*alt*-SacchA) and P(StyCl-*alt*-SacchA) to obtain, respectively, P(StyOMe-*alt*-AA) and P(StyCl-*alt*-AA).

Direct decarboxylation of acrylic acid units

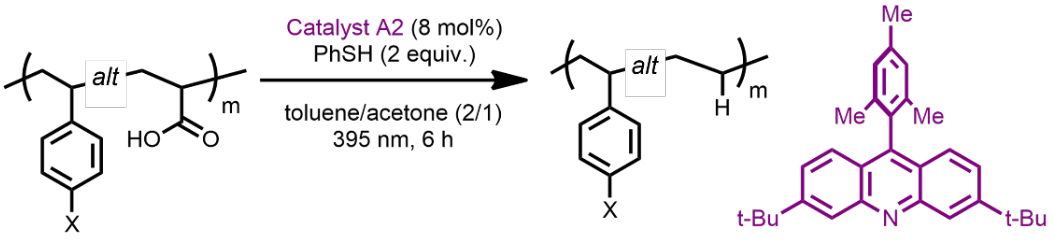

X = H, O-Me, Cl

To a flame-dried vial charged with a stir bar was added P(Sty-*alt*-AA) (70.5 mg) and acridine photocatalyst A2 (8 mol% with respect to AA units, 0.030 mmol, 13 mg). Acetone (2 mL) was added, and the solution was stirred until dissolution, followed by PhMe (1 mL). The vial was purged by bubbling nitrogen through the reaction mixture for 5 min. Thiophenol (2.0 equiv, 0.80 mmol, 82 µL) was added, and the reaction mixture was irradiated with 395 nm light at room temperature for 6 h. The solvent was removed, and the polymer was purified by precipitation in methanol (×3) to afford the pure P(Sty-*alt*-E).

P(StyOMe-*alt*-E) and P(StyCl-*alt*-E) were synthesized by using the same experimental reaction conditions using P(StyOMe-*alt*-AA) (82.5 mg) and P(StyCl-*alt*-AA) (84.3 mg).

2.4 General synthetic procedures for statistical P(StyX-*stat*-P) copolymers

RAFT copolymerization of styrene (derivatives) and tertbutyl methacrylate (tBuMA)

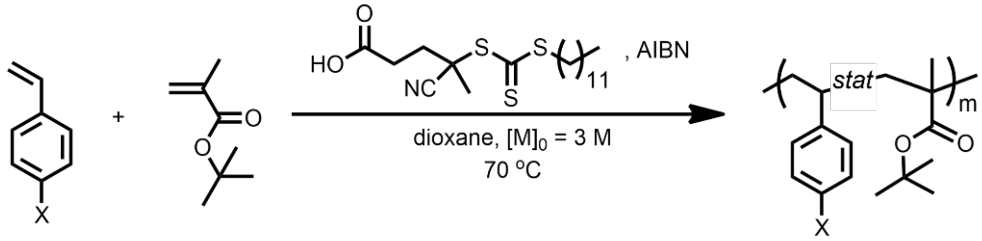

X = H, O-Me, Cl

CDP (1.0 equiv, 0.074 mmol, 30 mg) was combined with *t*BuMA (800 equiv, 60.0 mmol, 9.67 mL), styrene (1000 equiv, 60.00 mmol, 8.540 mL), AIBN (0.100 equiv, 7.00 µmol, 1.22 mg) in dioxane (40 mL) in a 100-mL Schlenk flask. The solution was sparged with argon at room temperature before submerging the flask in a preheated oil bath set to 70 ˚C. The polymerization was stopped at conversions ≤ 10 %. The resulting copolymer was dialyzed against acetone before removing the solvent under reduced pressure to yield pure P($Sty_{0.54}$-*stat*-*t*$BuMA_{0.46}$), which was characterized via ^{1}H NMR spectroscopy and SEC.

P(StyOMe$_{0.51}$-*stat-t*BuMA$_{0.49}$) was likewise synthesized using the ratio [Sty: *t*BuMA: CDP: AIBN] = [100: 100: 1: 0.1] in dioxane at 70 ˚C.

P(StyCl$_{0.47}$-*stat-t*BuMA$_{0.53}$) was synthesized using the ratio [StyCl: *t*BuMA: CDP: AIBN] = [500: 700: 1: 0.1] in dioxane at 70 ˚C.

Hydrolysis of t-butyl methacrylate units into methacrylic acids (MA)

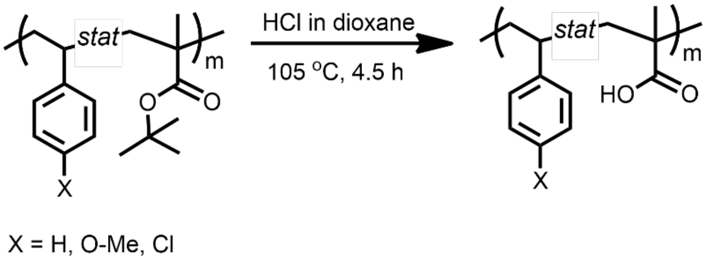

The copolymer P(Sty$_{0.54}$-*stat-t*BuMA$_{0.46}$) (0.562 g) was dissolved in dioxane (21 mL), and hydrochloric acid (2.1 mL, 37%) was added to the solution. The mixture was refluxed for 4.5 h at 105 ˚C. After completion of the hydrolysis, the polymer was precipitated in diethyl ether (×2) and dried under vacuum to yield P(Sty$_{0.54}$-*stat*-MA$_{0.46}$).

The same reaction conditions were used for the hydrolysis of the alternating copolymers P(StyOMe$_{0.51}$-*stat-t*BuMA$_{0.49}$) and P(StyCl$_{0.47}$-*stat-t*BuMA$_{0.53}$) to obtain, respectively, P(StyOMe$_{0.51}$-*stat*-MA$_{0.49}$) and P(StyCl$_{0.47}$-*stat*-MA$_{0.53}$).

Direct decarboxylation of methacrylic acid units

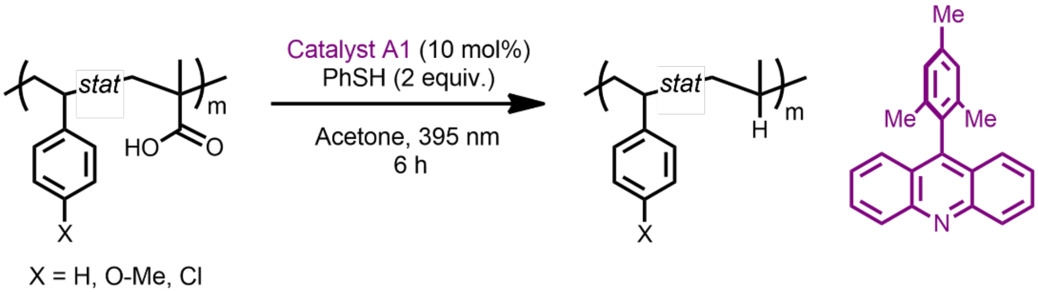

To a flame-dried vial charged with a stir bar was added P(Sty$_{0.54}$-*stat*-MA$_{0.46}$) (76.7 mg) and acridine photocatalyst A1 (10 mol% with respect to MA units, 0.040 mmol, 11 mg). Acetone (4 mL) was added, and the solution was stirred until dissolution. Following dissolution, the vial was purged by bubbling nitrogen through the reaction mixture for 5 min. Thiophenol (2.0 equiv, 0.70 mmol, 75 µL) was added, and the reaction mixture was irradiated with 395 nm light at room temperature for 6 h. The solvent was removed, and the polymer was purified by precipitation in methanol (×3) to afford the pure P(Sty$_{0.54}$-*stat*-P$_{0.46}$).

P(StyOMe$_{0.51}$-*stat*-P$_{0.49}$) was synthesized using the same experimental reaction conditions with P(StyOMe$_{0.51}$-*stat*-MA$_{0.49}$) (88.5 mg), acridine photocatalyst A1 (10 mol% with respect to MA units, 0.04 mmol, 11.7 mg), and thiophenol (2.0 equiv, 0.8 mmol, 80 µL) in acetone (3 mL).

P(StyCl$_{0.47}$-*stat*-P$_{0.53}$) was synthesized using the same experimental reaction conditions with P(StyCl$_{0.47}$-*stat*-MA$_{0.53}$) (88.6 mg), acridine photocatalyst A1 (10 mol% with respect to MA units, 0.04 mmol, 12.6 mg), and thiophenol (2.0 equiv, 0.8 mmol, 87 µL) in acetone (3 mL).

2.5 General synthetic procedures for statistical P(StyX-*stat*-E) copolymers

RAFT copolymerization of styrene (derivatives) and tertbutyl acrylate (tBuA)

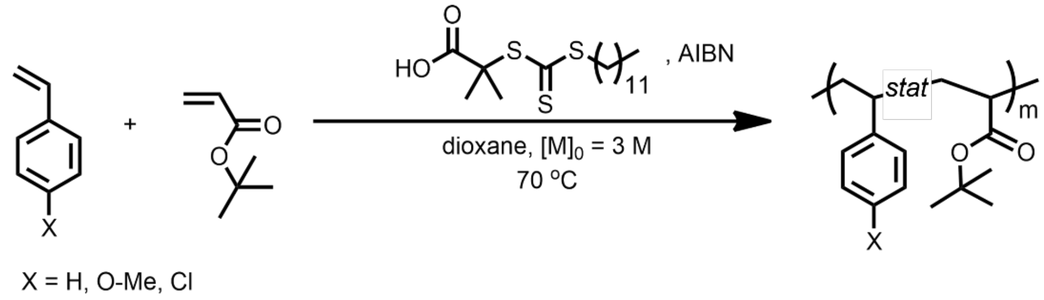

DDMAT (1.0 equiv, 0.096 mmol, 0.035 g) was combined with *t*BuA (1650 equiv, 159.0 mmol, 23.24 mL), styrene (600 equiv, 58.0 mmol, 6.62 mL), AIBN (0.100 equiv, 9.00 µmol, 1.58 mg) in dioxane (40 mL) in a 100-mL Schlenk flask. The solution was sparged with argon at room temperature before submerging the flask in a preheated oil bath set to 70 ˚C. The polymerization was stopped at conversions ≤ 10 %. The resulting copolymer was dialyzed against acetone before removing the solvent under reduced pressure to yield pure P(Sty$_{0.54}$-*stat*-*t*BuMA$_{0.46}$), which was characterized via ^{1}H NMR spectroscopy and SEC.

P(StyOMe$_{0.54}$-*stat*-*t*BuA$_{0.46}$) was likewise synthesized using the ratio [Sty: *t*BuMA: CDP: AIBN] = [120: 120: 1: 0.1] in dioxane at 70 ˚C.

P(StyCl$_{0.49}$-*stat*-*t*BuA$_{0.51}$) was synthesized using the ratio [StyCl: *t*BuMA: CDP: AIBN] = [300: 600: 1: 0.1] in dioxane at 70 ˚C.

Hydrolysis of t-butyl acrylate units into acrylic acids (AA)

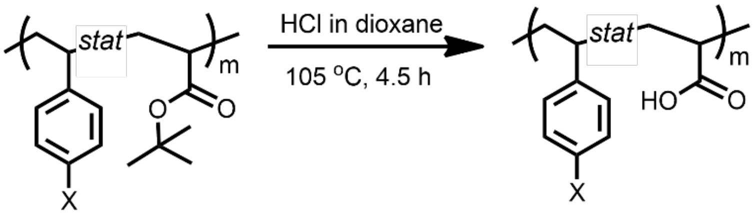

X = H, O-Me, Cl

The copolymer P(Sty$_{0.54}$-*stat*-*t*BuA$_{0.46}$) (0.627 g) was dissolved in dioxane (26 mL), and hydrochloric acid (2.6 mL, 37%) was added to the solution. The mixture was refluxed for 4.5 h at 105 ˚C. After completion of the hydrolysis, the polymer was precipitated in diethyl ether (×2) and dried under vacuum to afford the copolymer P(Sty$_{0.54}$-*stat*-AA$_{0.46}$).

The same reaction conditions were used for the hydrolysis of the alternating copolymers P(StyOMe$_{0.54}$-*stat*-*t*BuA$_{0.46}$) and P(StyCl$_{0.49}$-*stat*-*t*BuA$_{0.51}$) to obtain, respectively, P(StyOMe$_{0.54}$-*stat*-AA$_{0.46}$) and P(StyCl$_{0.49}$-*stat*-AA$_{0.51}$).

Direct decarboxylation of acrylic acid units

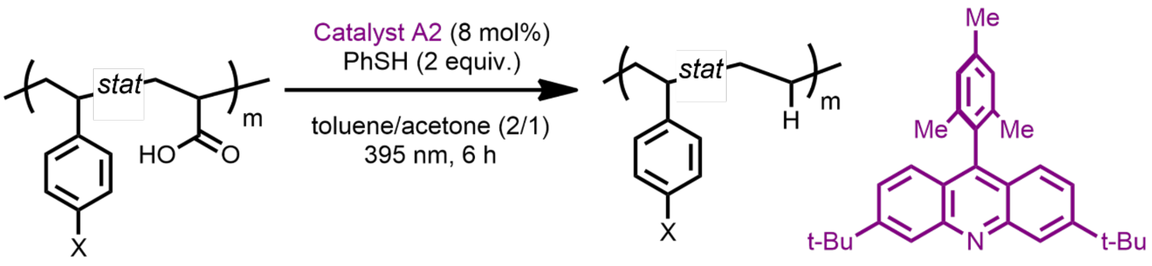

X = H, O-Me, Cl

To a flame-dried vial charged with a stir bar was added P(Sty$_{0.47}$-*stat*-AA$_{0.53}$) (69.7 mg) and acridine photocatalyst A2 (8 mol% with respect to AA units, 0.030 mmol, 14 mg). Acetone (2 mL) was added, and the solution was stirred until dissolution, followed by PhMe (1 mL). The vial was purged by bubbling nitrogen through the reaction mixture for 5 min. Thiophenol (2.0 equiv, 0.80 mmol, 87 µL) was added, and the reaction mixture was irradiated with 395 nm light at room temperature for 6 h. The solvent was removed, and the polymer was purified by precipitation in methanol (×3) to afford the pure P(Sty$_{0.47}$-*stat*-E$_{0.53}$).

P(StyOMe$_{0.54}$-*stat*-E$_{0.46}$) was synthesized using the same experimental reaction conditions with P(StyOMe$_{0.54}$-*stat*-AA$_{0.46}$) (84.5 mg), acridine photocatalyst A1 (8 mol% with respect to AA units, 0.030 mmol, 12 mg), and thiophenol (2.0 equiv, 0.70 mmol, 75 µL).

P(StyCl$_{0.49}$-*stat*-E$_{0.51}$) was synthesized using the same experimental reaction conditions with P(StyCl$_{0.49}$-*stat*-AA$_{0.51}$) (83.7 mg), acridine photocatalyst A1 (8 mol% with respect to AA units, 30.0 µmol, 13.4 mg), and thiophenol (2.0 equiv, 0.80 mmol, 83 µL).

2.6 Free radical polymerization of SacchA

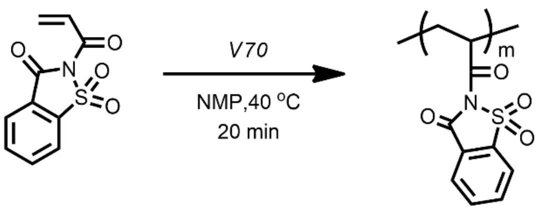

In a flame-dried vial, SacchA (100 equiv, 0.840 mmol, 0.200 g) was dissolved in anhydrous NMP (3.5 mL) and sparged with argon at room temperature for 15 min. The initiator *V70* (0.20 equiv, 0.020 mmol, 5.2 mg) was added under inert atmosphere, and the polymerization mixture was immersed in a preheated oil bath at 40 ˚C. The polymerization was conducted for 20 min and was then quenched with air to afford the homopolymer (P(SacchA)).

SEC characterization

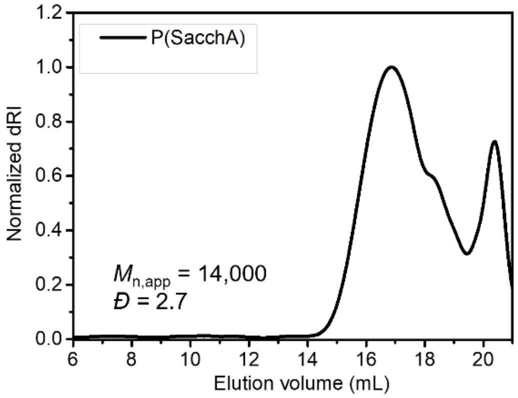

Figure S1. *SEC trace of crude P(SacchA) obtained by conventional radical polymerization.*

2.7 Determination of monomer reactivity ratio by the Fineman-Ross method

The monomer reactivity ratio was calculated by defining SacchA as M_1 and Sty as M_2. Several conventional radical copolymerizations of SacchA and Sty were conducted (*V70* as in initiator in NMP at 40 ˚C) with different monomer feeds ($[M_1]_0$: $[M_2]_0$ = 90:10, 70:30, 50:50, 40:60, 30:70, and 10:90). The monomer reactivity ratios r_1 and r_2 of the copolymer was calculated from the Fineman-Ross equation (1):

$$\mathbf{G} = \boldsymbol{r}\mathbf{1}\mathbf{H} - \boldsymbol{r}\mathbf{2} \qquad (1)$$

With $G = \frac{F\,(f-1)}{f}$

$$H = r1\left(\frac{F^2}{f}\right) - r2$$

$F = M_1/M_2$ (molar ratio for monomer feed composition)

$f = d[\mathrm{M}_1]/d[\mathrm{M}_2] = m_1/m_2$ (molar ratio for copolymer composition)

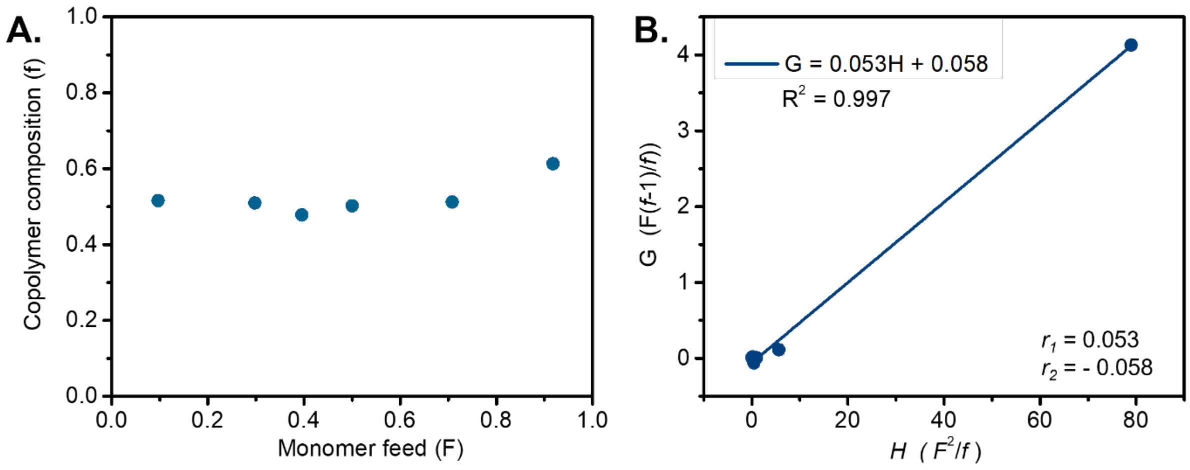

Figure S2. *A) Copolymer composition vs. monomer feed graph. B) Determination of monomer reactivity ratio of SacchA and Sty by the Fineman-Ross method.*

M_1 (SacchA)	M_2 (Sty)	m_1 (SacchA)	m_2 (Sty)	F ($[M_1]/[M_2]$)	f (m_1/m_2)	H (F^2/f)	G ($F(f-1)/f$)
0.92	0.08	0.61	0.39	11.19	1.58	78.96	4.13
0.71	0.29	0.51	0.49	2.43	1.05	5.62	0.11
0.50	0.50	0.50	0.50	1.00	1.01	0.99	0.01
0.40	0.60	0.48	0.52	0.66	0.91	0.47	-0.06
0.30	0.70	0.51	0.49	0.42	1.04	0.17	0.02
0.10	0.90	0.52	0.48	0.11	1.07	0.01	0.01

2.8 Photocatalyst studies

Photocatalyst coupling reaction studies on P(Sty$_{0.56}$-stat-AA$_{0.44}$)

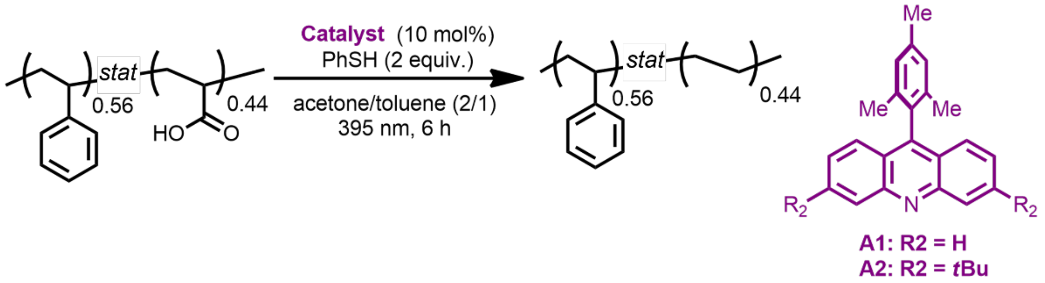

To a flame-dried vial charged with a stir bar was added P(Sty$_{0.56}$-*stat*-AA$_{0.44}$) (72.0 mg) and acridine photocatalyst A1 or A2 (10 mol%). Acetone (2 mL) was added, and the solution was stirred until dissolution, followed by PhMe (1 mL). The vial was purged by bubbling nitrogen through the reaction mixture for 5 min. Thiophenol (2.0 equiv, 0.70 mmol, 72 μL) was added, and the reaction mixture was irradiated with 395 nm light at room temperature for 6 h. The solvent was removed, and the polymer was purified by precipitation in methanol (×3).

The isolated polymers were characterized by ^{1}H NMR spectroscopy and SEC chromatography.

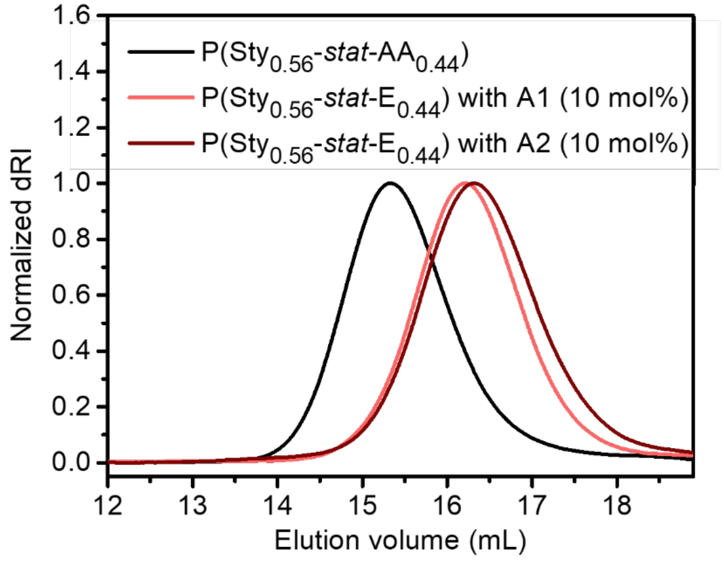

Figure S3. *SEC traces of P(Sty$_{0.56}$-stat-AA$_{0.44}$) (black trace) and P(Sty$_{0.56}$-stat-E$_{0.44}$) using 10 mol% of photocatalyst A1 (pink trace) or A2 (brown trace).*

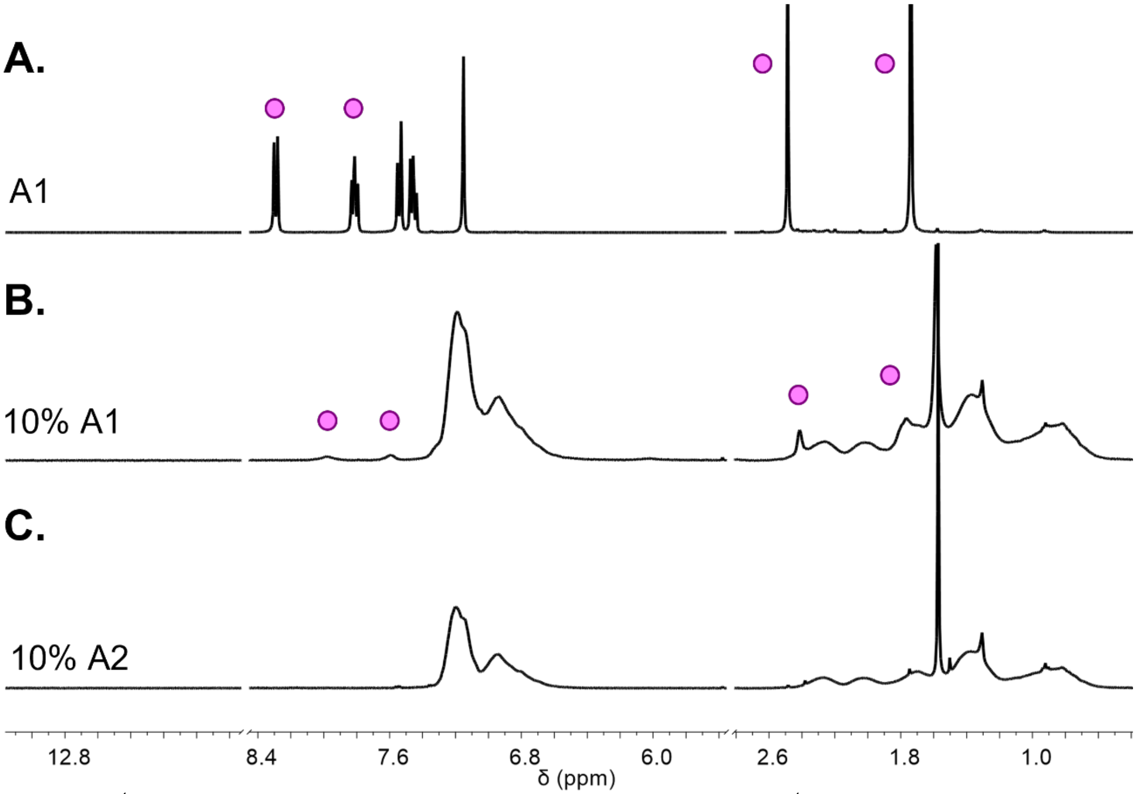

Figure S4. *A) ^{1}H NMR (CD_2Cl_2) spectrum of photocatalyst A1. B) ^{1}H NMR (CD_2Cl_2) spectrum of* P(Sty$_{0.56}$-*stat*-E$_{0.44}$) *using 10 mol% of photocatalyst A1 showing photocatalyst addition to the backbone. C) ^{1}H NMR (CD_2Cl_2) spectrum of* P(Sty$_{0.56}$-*stat*-E$_{0.44}$) *using 10 mol% of photocatalyst A2 showing no catalyst addition to the polymer backbone.*

A1 catalyst loading study for the decarboxylation of MA units on P(Sty$_{0.45}$-stat-MA$_{0.55}$)

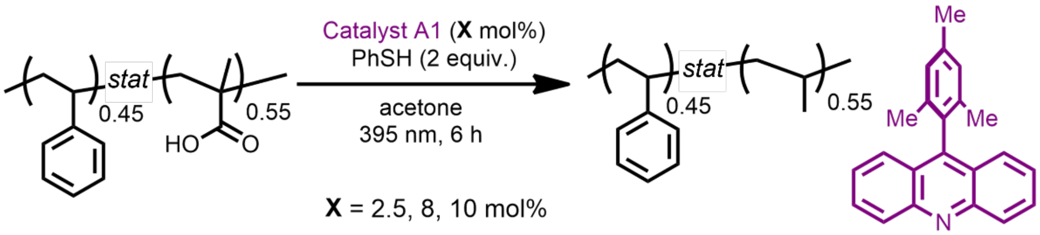

To a flame-dried vial charged with a stir bar was added P(Sty$_{0.45}$-*stat*-MA$_{0.55}$) (75.4 mg) and acridine photocatalyst A1 (**X** mol% with respect to MA units). Acetone (3 mL) was added, and the solution was stirred until dissolution. Following dissolution, the vial was purged by bubbling nitrogen through the reaction mixture for 5 min. Thiophenol (2.0 equiv, 0.90 mmol, 90 µL) was added, and the reaction mixture was irradiated with 395 nm light at room temperature for 6 h. The solvent was removed, and the polymer was purified by precipitation in methanol (×3).

The isolated polymers were characterized by ^{1}H NMR spectroscopy and SEC chromatography. In all cases, the proton NMR showed quantitative decarboxylation.

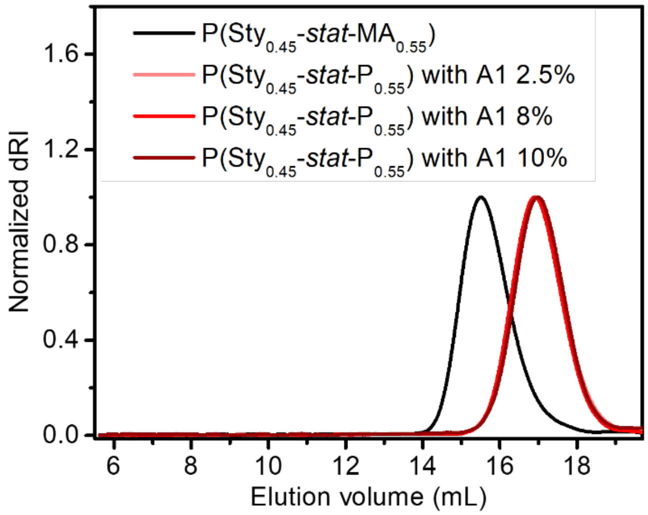

Figure S5. *SEC traces of* P($Sty_{0.45}$-*stat*-$MA_{0.55}$) *and P($Sty_{0.45}$-stat-$P_{0.55}$) using 2.5, 8, and 10 mol% of photocatalyst A1.*

A2 catalyst loading study for the decarboxylation of *AA units on P($Sty_{0.56}$-stat-$AA_{0.44}$)*

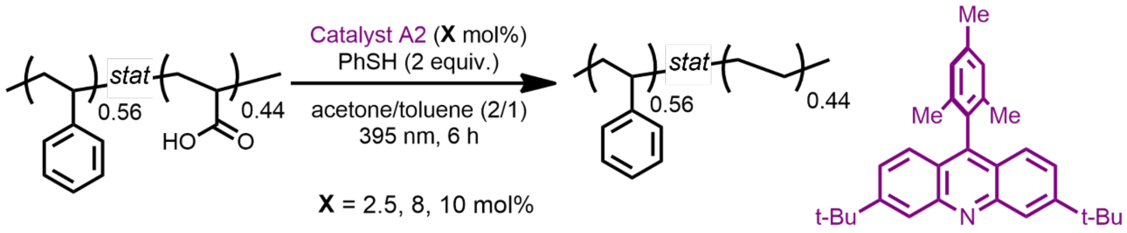

To a flame-dried vial charged with a stir bar was added P($Sty_{0.56}$-*stat*-$AA_{0.44}$) (72.0 mg) and acridine photocatalyst A2 (**X** mol%). Acetone (2 mL) was added, and the solution was stirred until dissolution, followed by PhMe (1 mL). The vial was purged by bubbling nitrogen through the reaction mixture for 5 min. Thiophenol (2.0 equiv, 0.70 mmol, 72 μL) was added, and the reaction mixture was irradiated with 395 nm light at room temperature for 6 h. The solvent was removed, and the polymer was purified by precipitation in methanol (×3).

The isolated polymers were characterized by ^{1}H NMR spectroscopy and SEC chromatography. In all cases, the proton NMR showed quantitative decarboxylation.

SEC Chromatogram

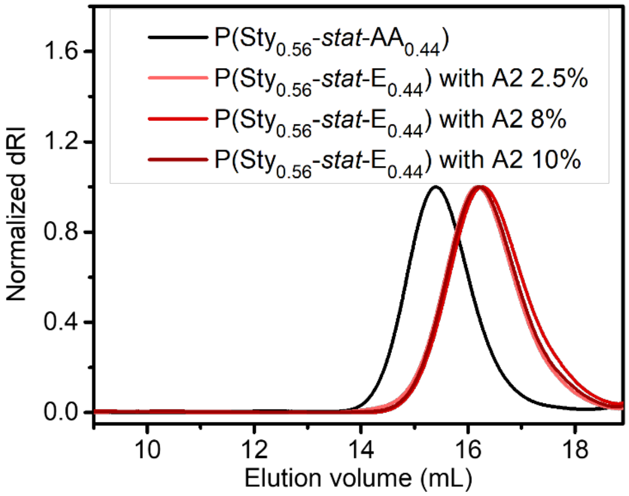

***Figure S6**. SEC traces of P(Sty$_{0.56}$-stat-AA$_{0.44}$) and P(Sty$_{0.56}$-stat-E$_{0.44}$) using 2.5, 8, and 10 mol% of photocatalyst A2.*

3. Characterization

3.1 RAFT Polymerization Data

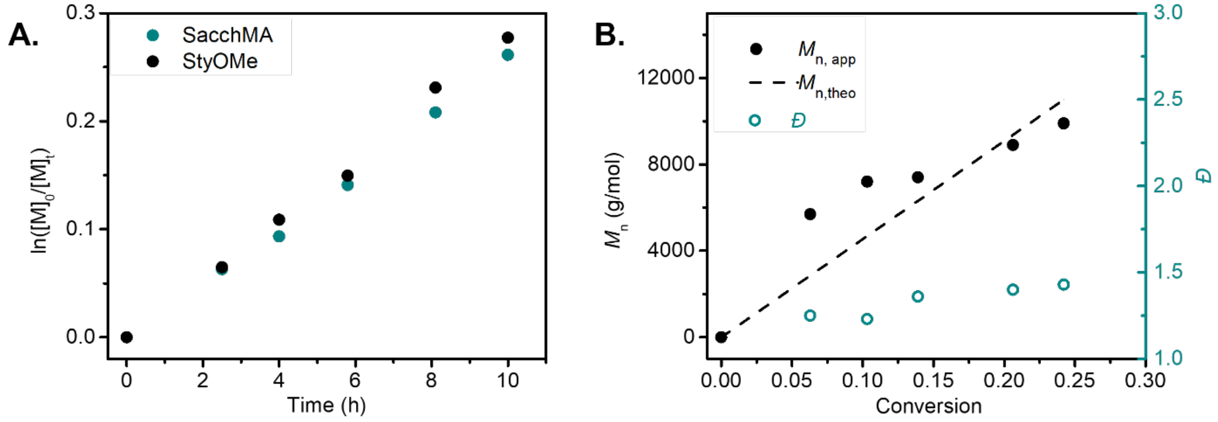

Figure S7. *A) Pseudo-first-order kinetic plot of StyOMe and SacchMA during RAFT copolymerization to synthesize P(StyOMe-alt-SacchMA). B) Evolution of number-average molecular weight and dispersity with monomer conversion.*

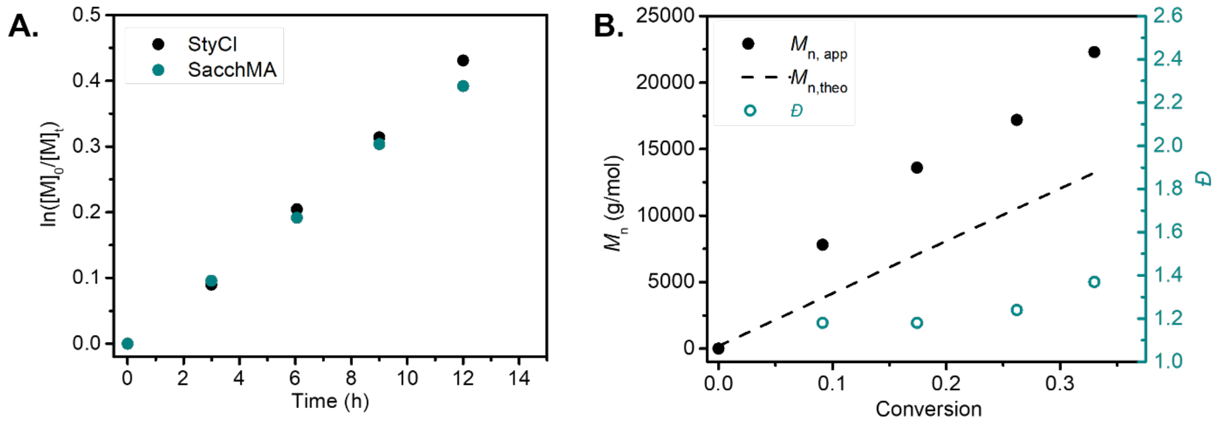

Figure S8. *A) Pseudo-first-order kinetic plot of StyCl and SacchMA during RAFT copolymerization to synthesize P(StyCl-alt-SacchMA). B) Evolution of number-average molecular weight and dispersity with monomer conversion.*

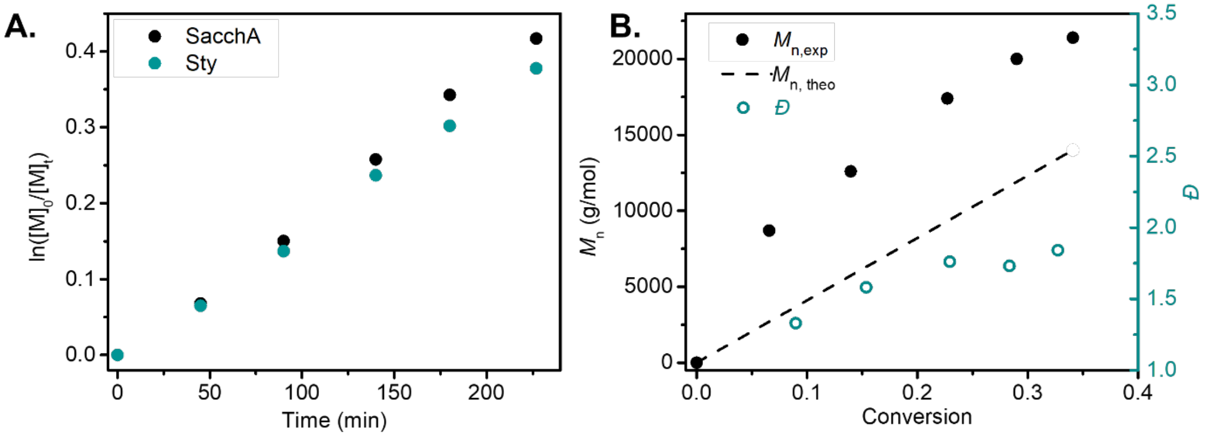

Figure S9. *A) Pseudo-first-order kinetic plot of Sty and SacchA during RAFT copolymerization to synthesize P(Sty-alt-SacchA). B) Evolution of number-average molecular weight and dispersity with monomer conversion.*

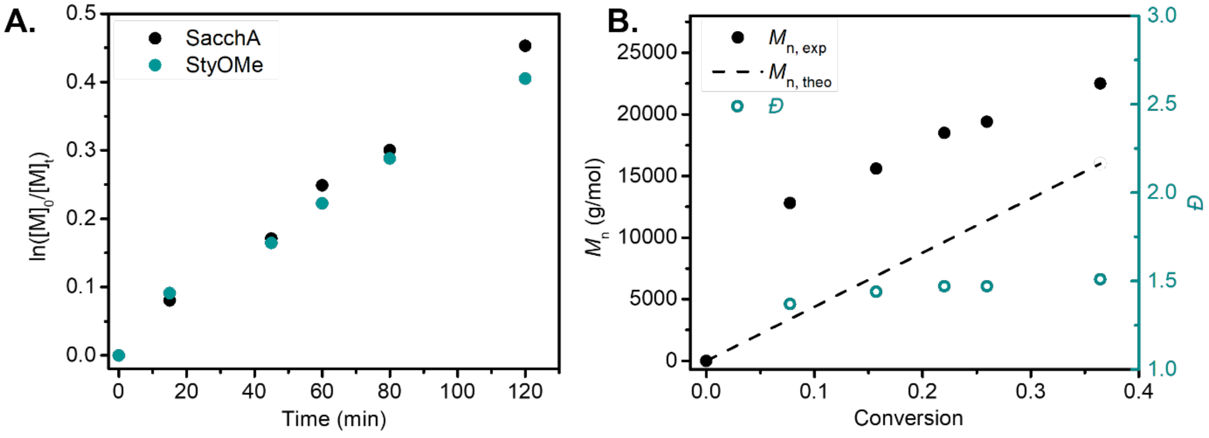

Figure S10. *A) Pseudo-first-order kinetic plot of StyOMe and SacchA during RAFT copolymerization to synthesize P(StyOMe-alt-SacchA). B) Evolution of number-average molecular weight and dispersity with monomer conversion.*

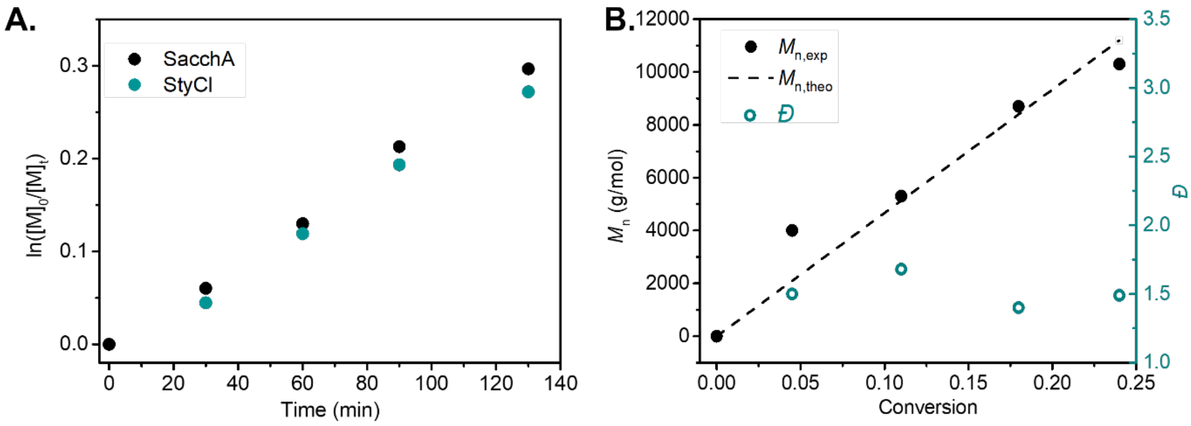

Figure S11. *Pseudo-first-order kinetic plot of StyCl and SacchA during RAFT copolymerization to synthesize P(StyCl-alt-SacchA). B) Evolution of number-average molecular weight and dispersity with monomer conversion.*

3.2 SEC Analysis

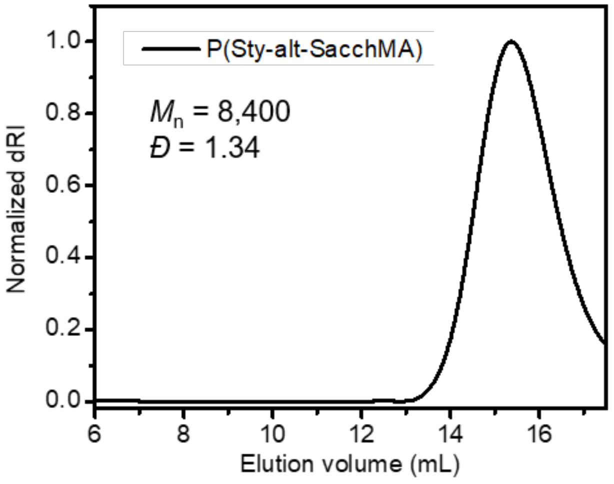

Figure S12. *SEC chromatogram of P(Sty-alt-SacchMA).*

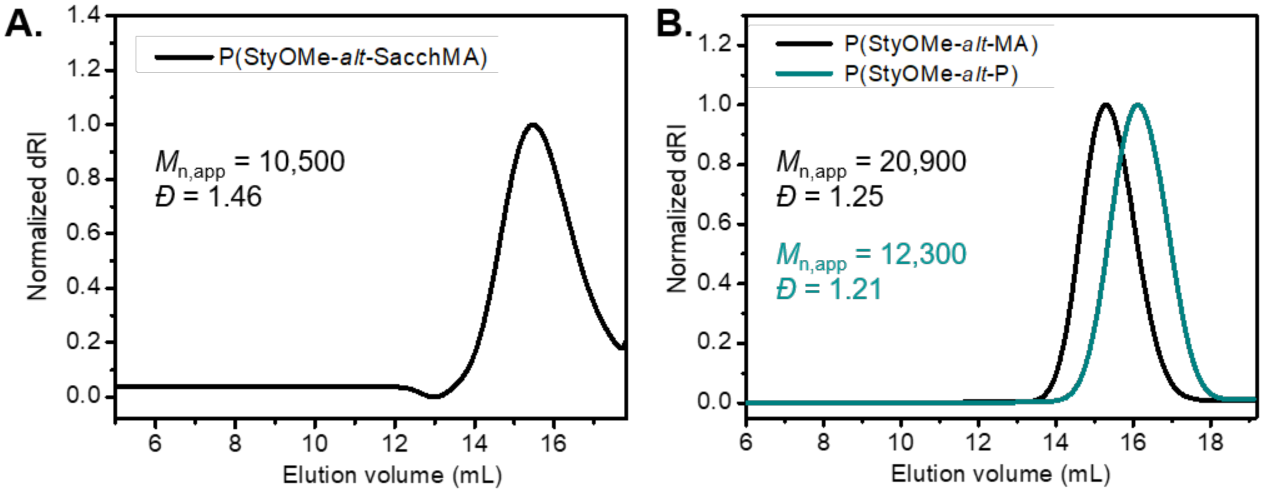

Figure S13. *A) SEC chromatogram of P(StyOMe-alt-SacchMA). B) SEC chromatograms of isolated P(StyOMe-alt-MA) after hydrolysis and P(StyOMe-alt-P) after decarboxylation.*

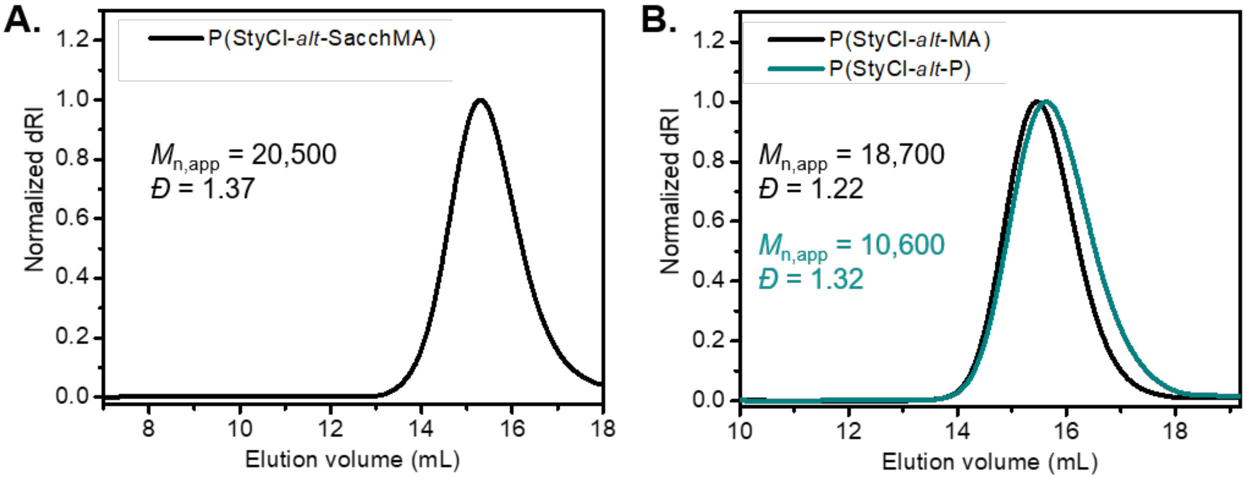

Figure S14. *A) SEC chromatogram of P(StyCl-alt-SacchMA). B) SEC chromatograms of isolated P(StyCl-alt-MA) after hydrolysis and P(StyCl-alt-P) after decarboxylation.*

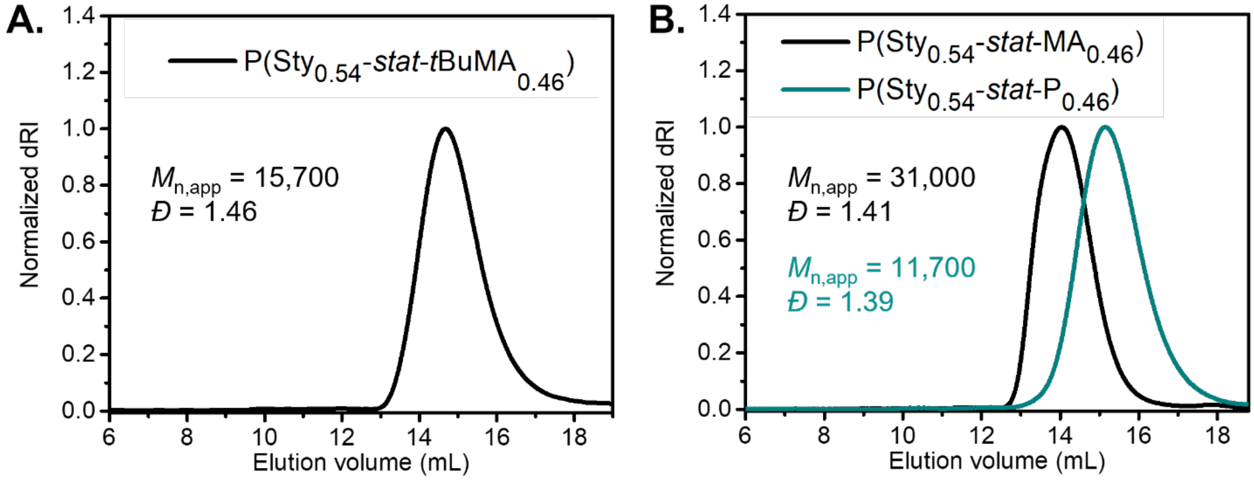

Figure S15. *SEC chromatogram of P(Sty$_{0.54}$-stat-tBuMA$_{0.46}$). B) SEC chromatograms of isolated P(Sty$_{0.54}$-stat-MA$_{0.46}$) after hydrolysis and P(Sty$_{0.54}$-stat-P$_{0.46}$) after decarboxylation.*

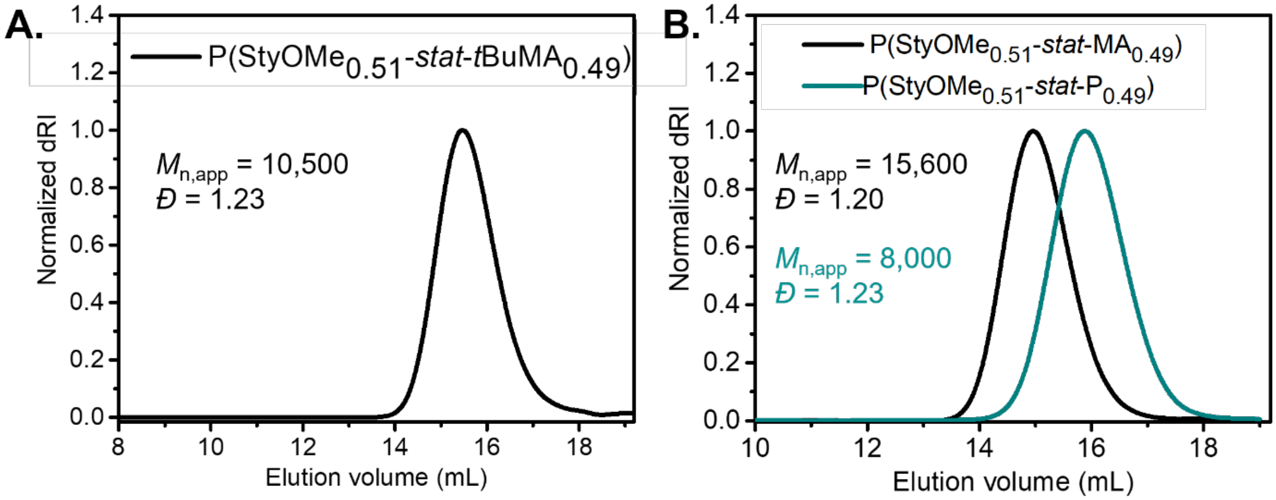

Figure S16. *SEC chromatogram of P(StyOMe$_{0.51}$-stat- tBuMA$_{0.49}$). B) SEC chromatograms of isolated P(StyOMe$_{0.51}$-stat-MA$_{0.49}$) after hydrolysis and P(StyOMe$_{0.51}$-stat-P$_{0.49}$) after decarboxylation.*

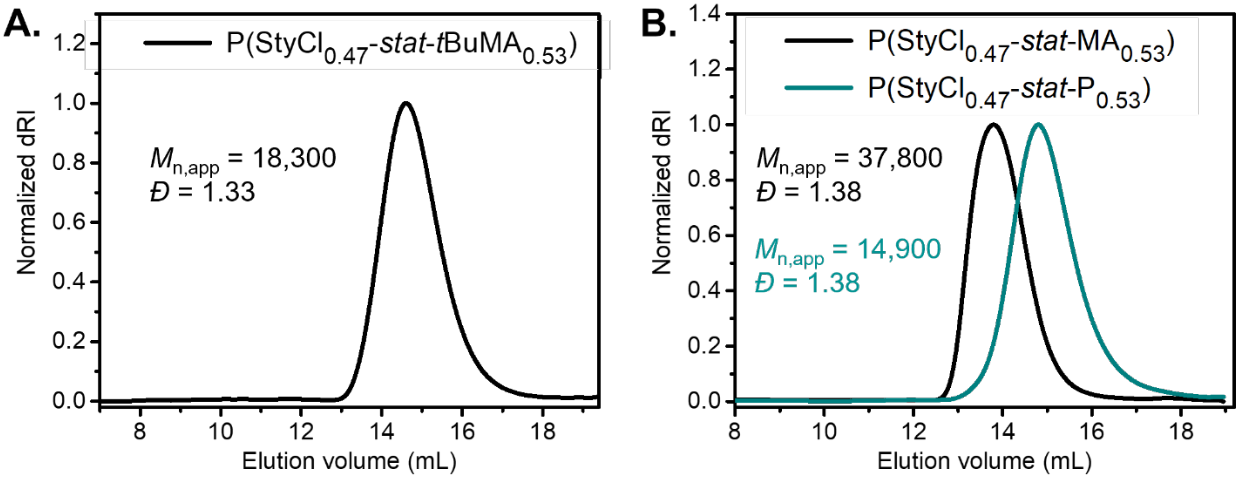

Figure S17. *A) SEC chromatogram of P(StyCl$_{0.47}$-stat-tBuMA$_{0.53}$). B) SEC chromatograms of isolated P(StyCl$_{0.47}$-stat-MA$_{0.53}$) after hydrolysis and P(StyCl$_{0.47}$-stat-P$_{0.53}$) after decarboxylation.*

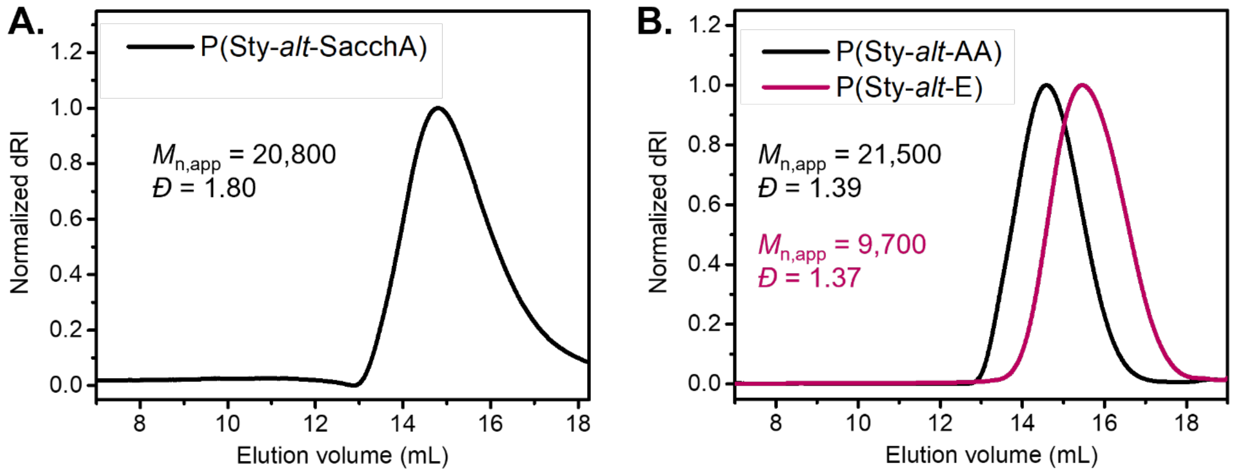

Figure S18. *A) SEC chromatogram of P(Sty-alt-SacchA). B) SEC chromatograms of isolated P(Sty-alt-AA) after hydrolysis and P(Sty-alt-E) after decarboxylation.*

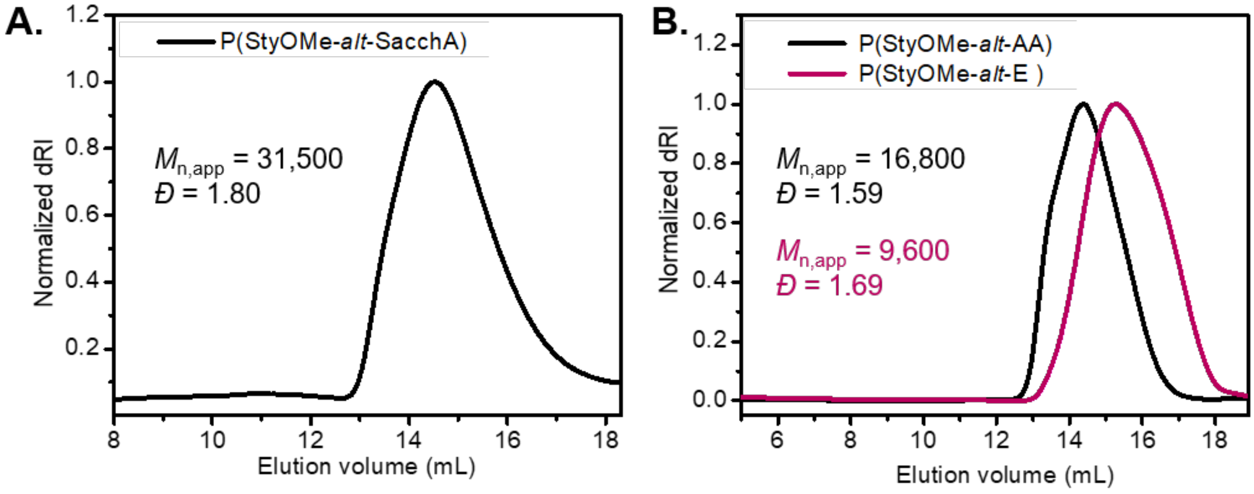

Figure S19. *A) SEC chromatogram of P(StyOMe-alt-SacchA). B) SEC chromatograms of isolated P(StyOMe-alt-AA) after hydrolysis and P(StyOMe-alt-E) after decarboxylation.*

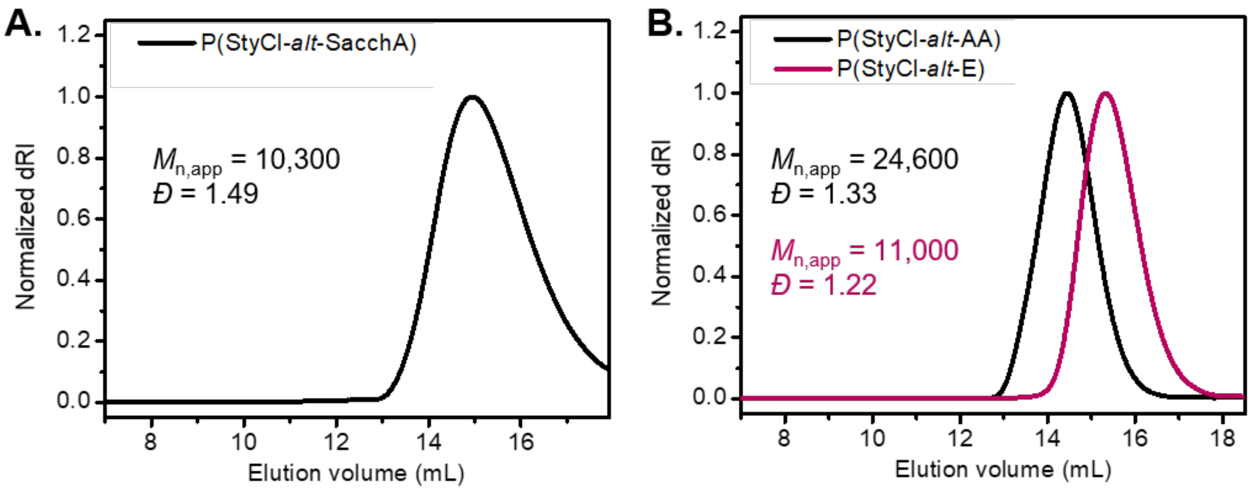

Figure S20. *A) SEC chromatogram of P(StyCl-alt-SacchA). B) SEC chromatograms of isolated P(StyCl-alt-AA) after hydrolysis and P(StyCl-alt-E) after decarboxylation.*

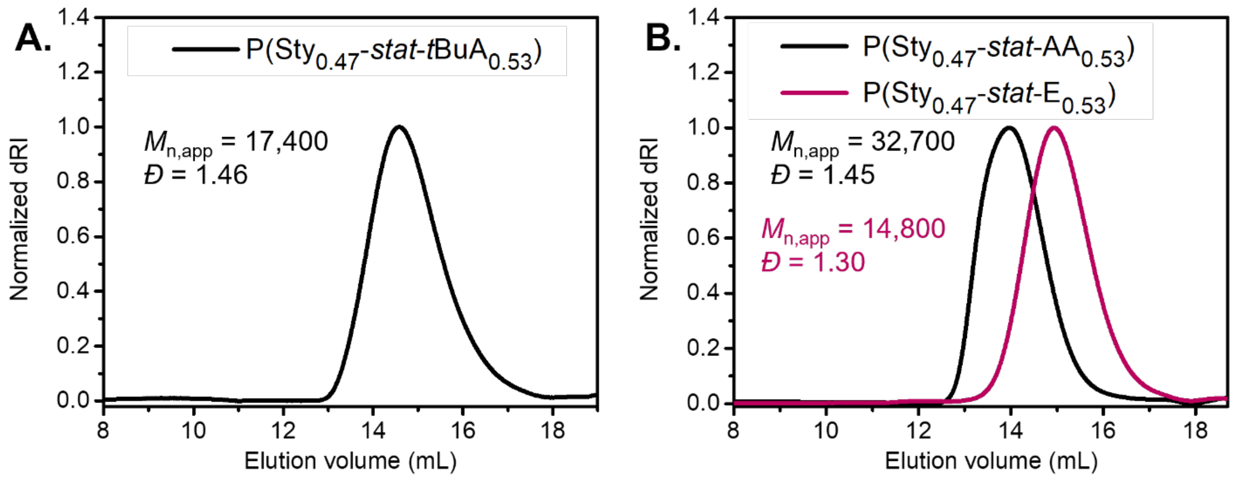

Figure S21. *A) SEC chromatogram of P(Sty$_{0.47}$-stat-tBuA$_{0.53}$). B) SEC chromatograms of isolated P(Sty$_{0.47}$-stat-AA$_{0.53}$) after hydrolysis and P(Sty$_{0.47}$-stat-E$_{0.53}$) after decarboxylation.*

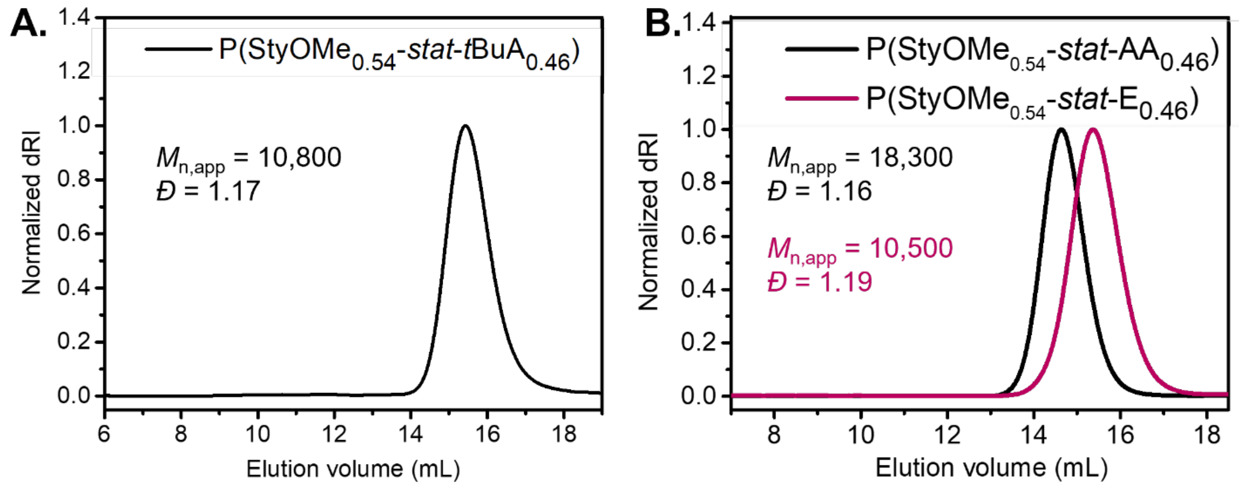

Figure S22. *A) SEC chromatogram of P(StyOMe$_{0.54}$-stat-tBuA$_{0.46}$). B) SEC chromatograms of isolated P(StyOMe$_{0.54}$-stat-AA$_{0.46}$) after hydrolysis and P(StyOMe$_{0.54}$-stat-E$_{0.46}$) after decarboxylation.*

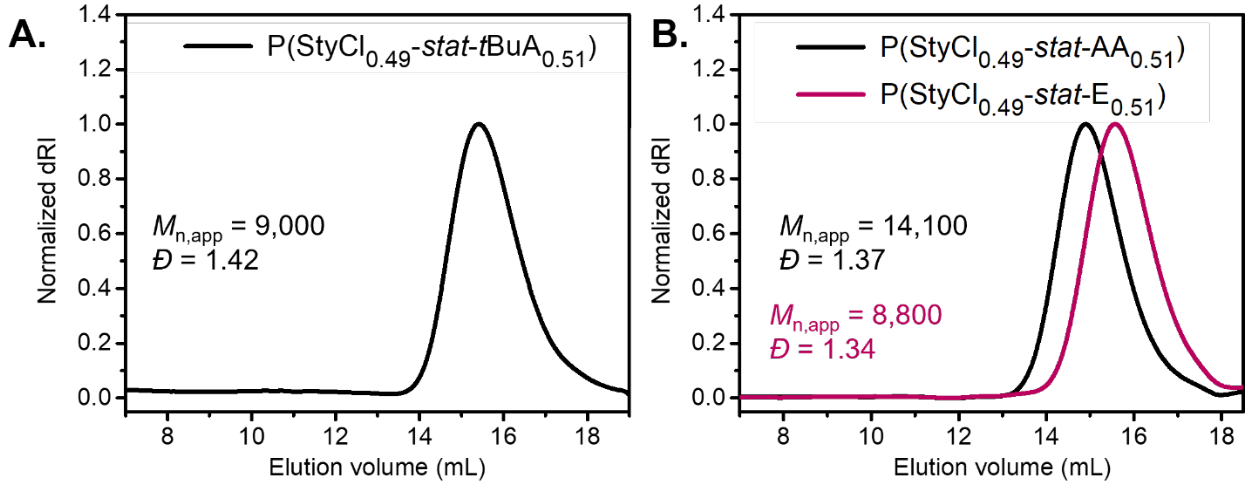

Figure S23. *A) SEC chromatogram of P(StyCl$_{0.49}$-stat-tBuA$_{0.51}$). B) SEC chromatograms of isolated P(StyCl$_{0.49}$-stat-AA$_{0.51}$) after hydrolysis and P(StyCl$_{0.49}$-stat-E$_{0.51}$) after decarboxylation.*

3.3 NMR characterizations

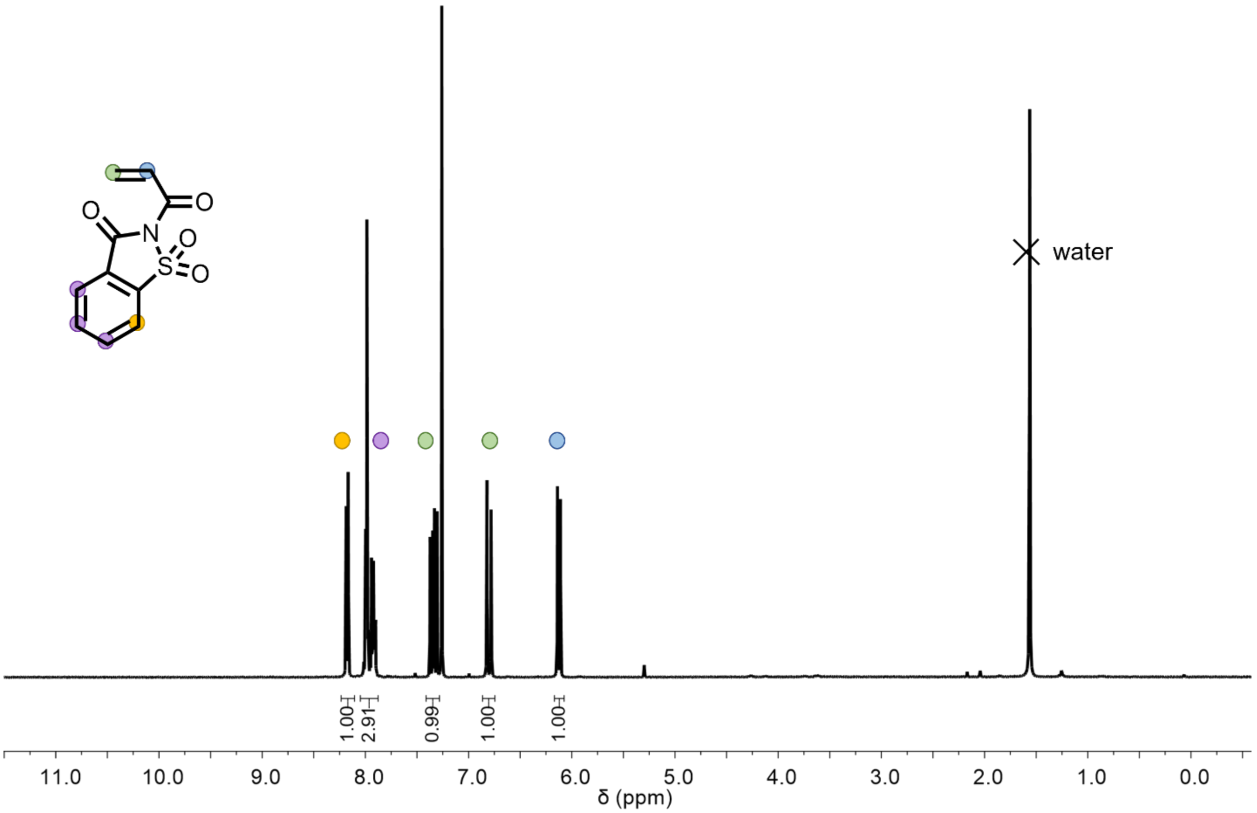

Figure S24. *^{1}H NMR spectrum of the saccharin acrylamide monomer in $CDCl_3$.*

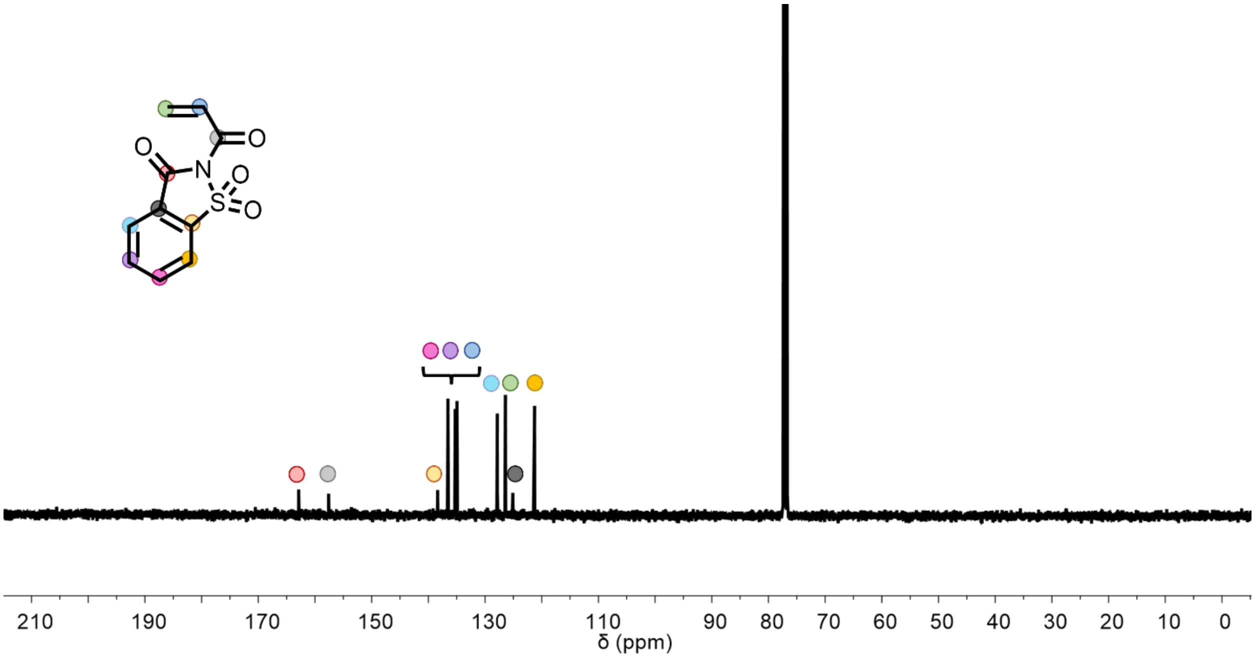

Figure S25. *^{13}C NMR spectrum of the saccharin acrylamide monomer in $CDCl_3$.*

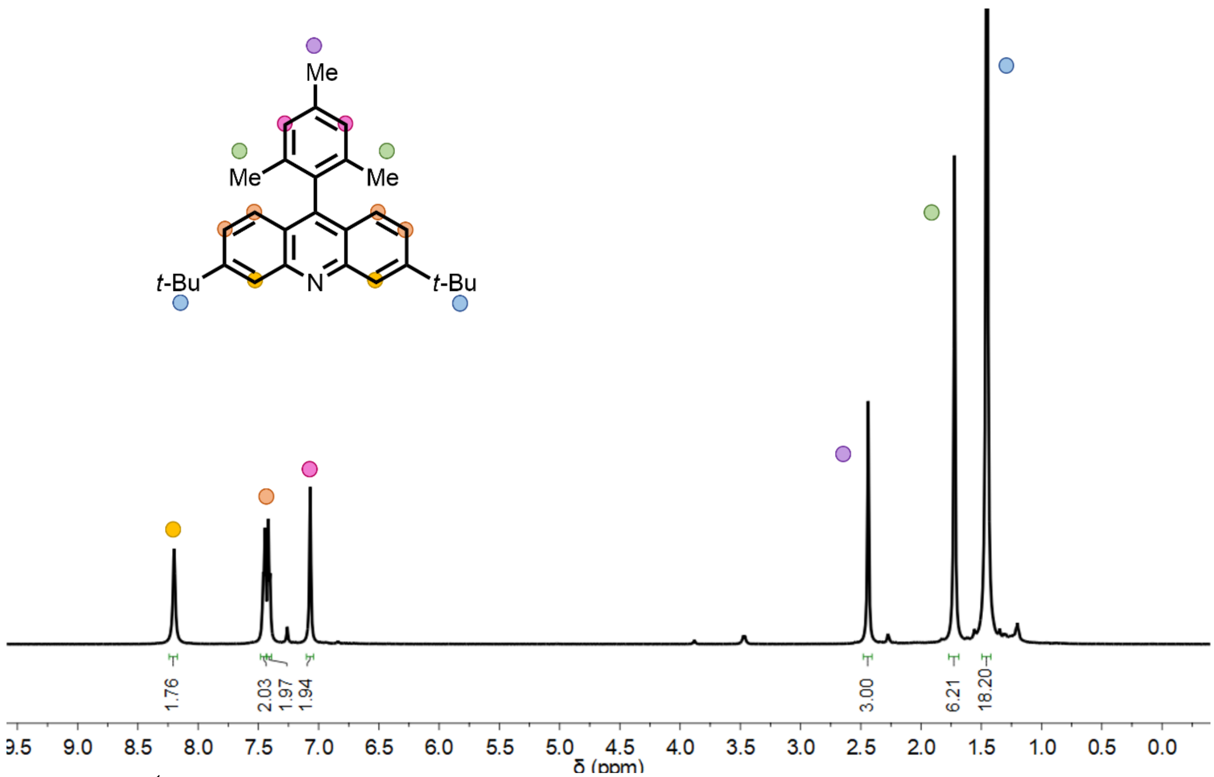

Figure S26. *^{1}H NMR spectrum of the photocatalyst A2 in $CDCl_3$.*

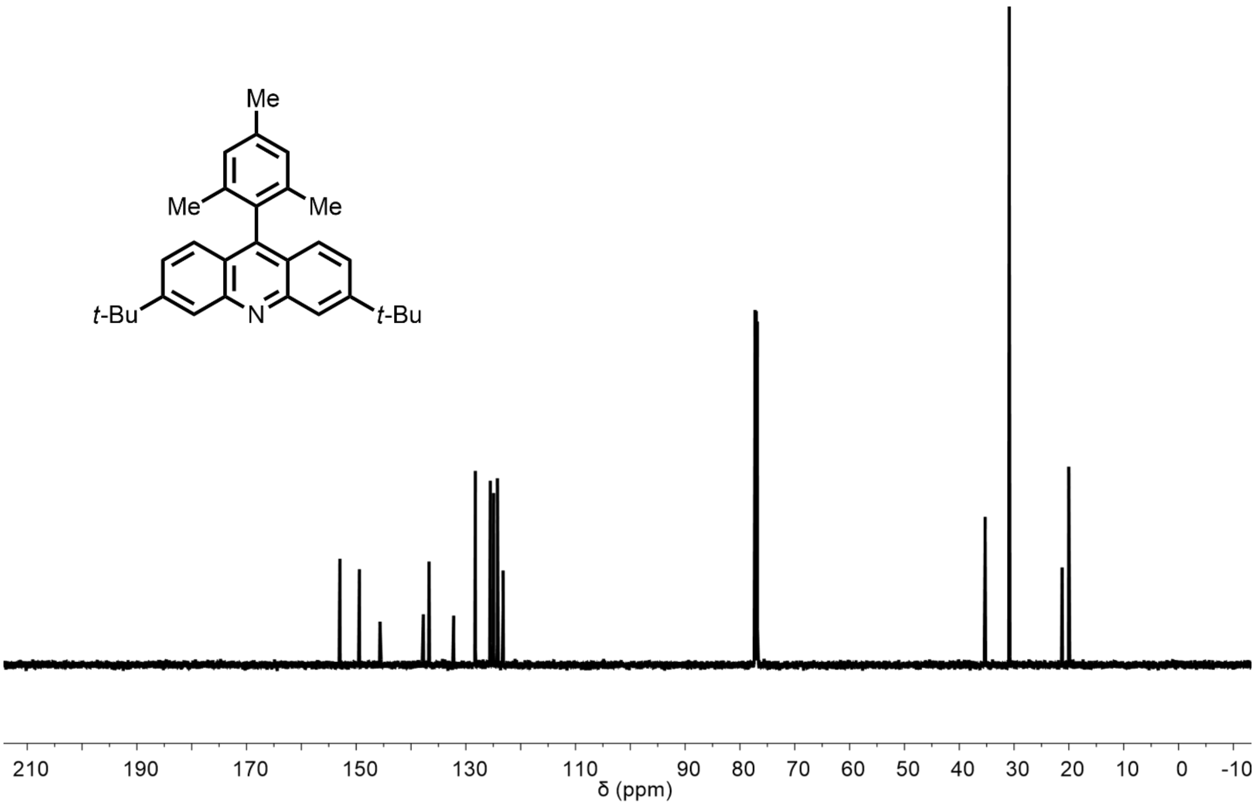

Figure S27. *^{13}C NMR spectrum of the photocatalyst A2 in $CDCl_3$.*

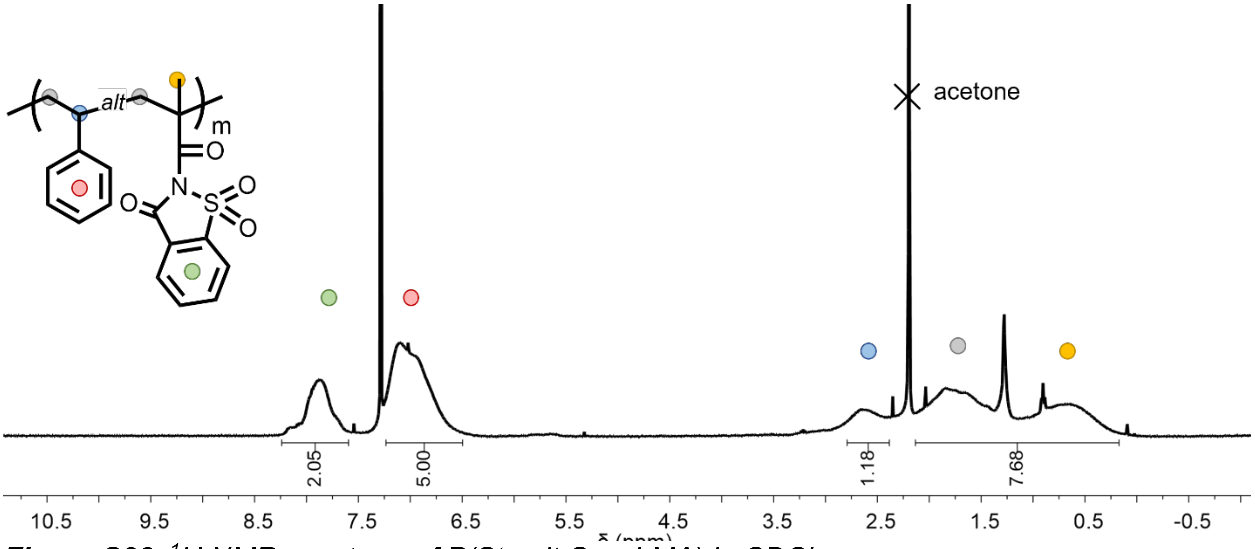

Figure S28. *^{1}H NMR spectrum of P(Sty-alt-SacchMA) in $CDCl_3$.*

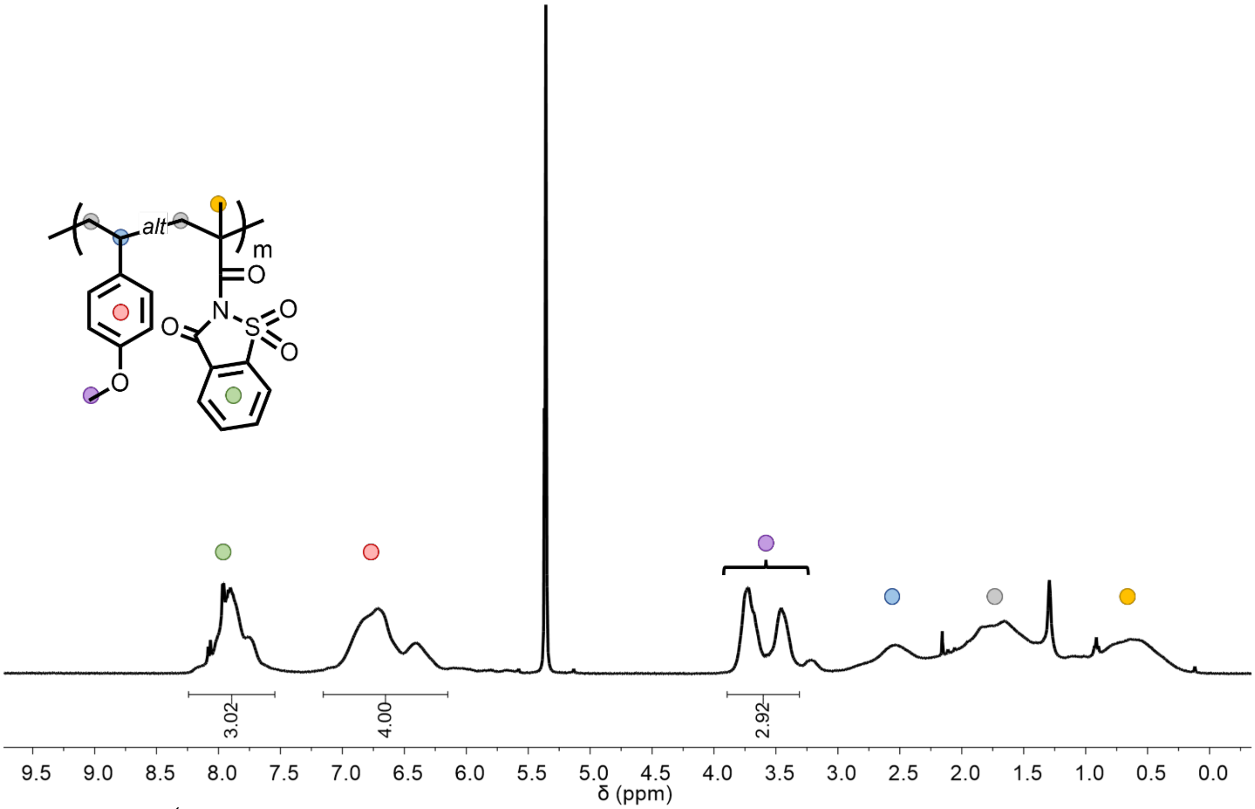

Figure S29. *^{1}H NMR spectrum of P(StyOMe-alt-SacchMA) in CD_2Cl_2.*

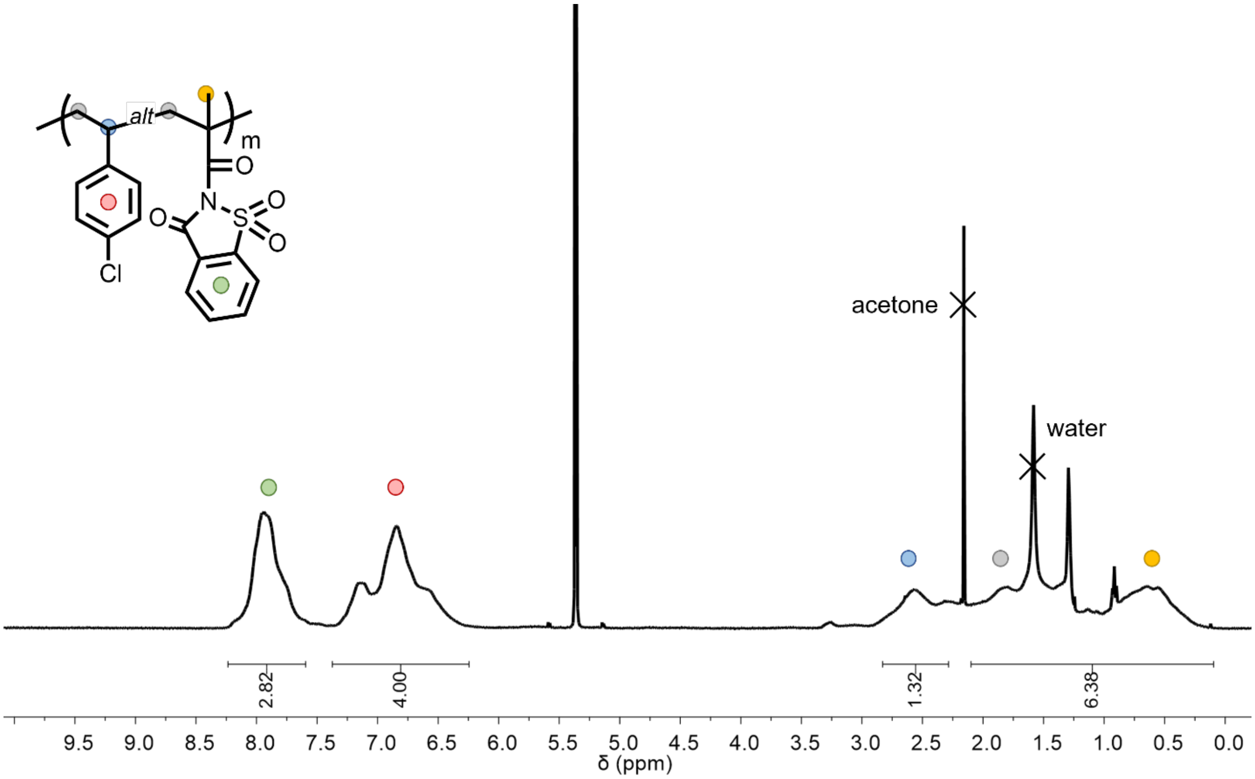

Figure S30. *^{1}H NMR spectrum of P(StyCl-alt-SacchMA) in CD_2Cl_2.*

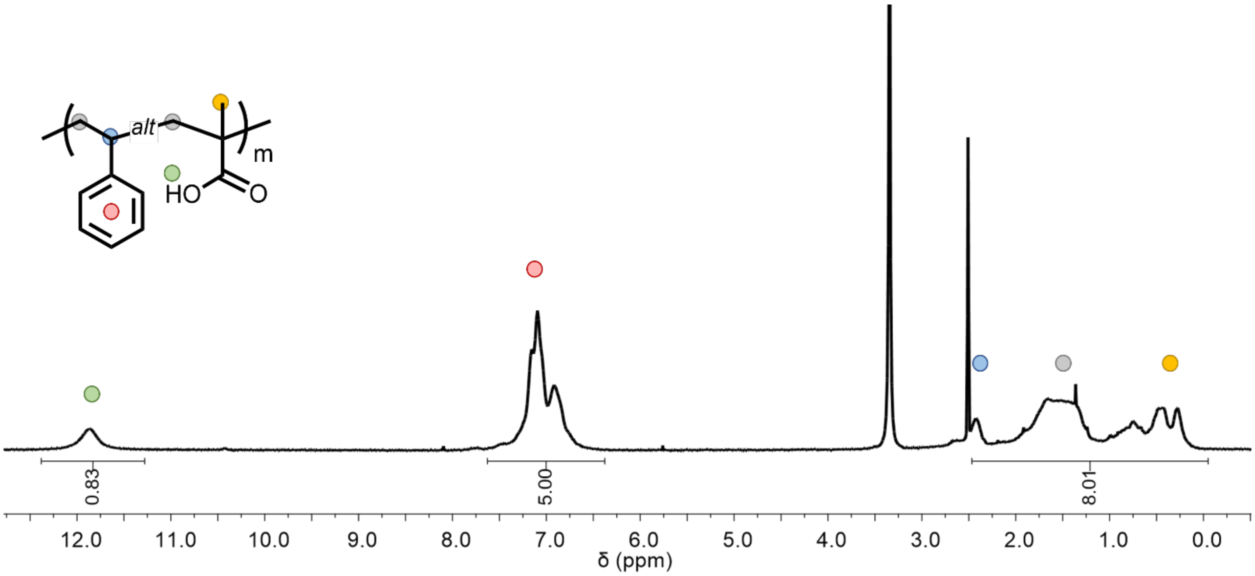

Figure S31. *^{1}H NMR spectrum of P(Sty-alt-MA) in DMSO-d_6.*

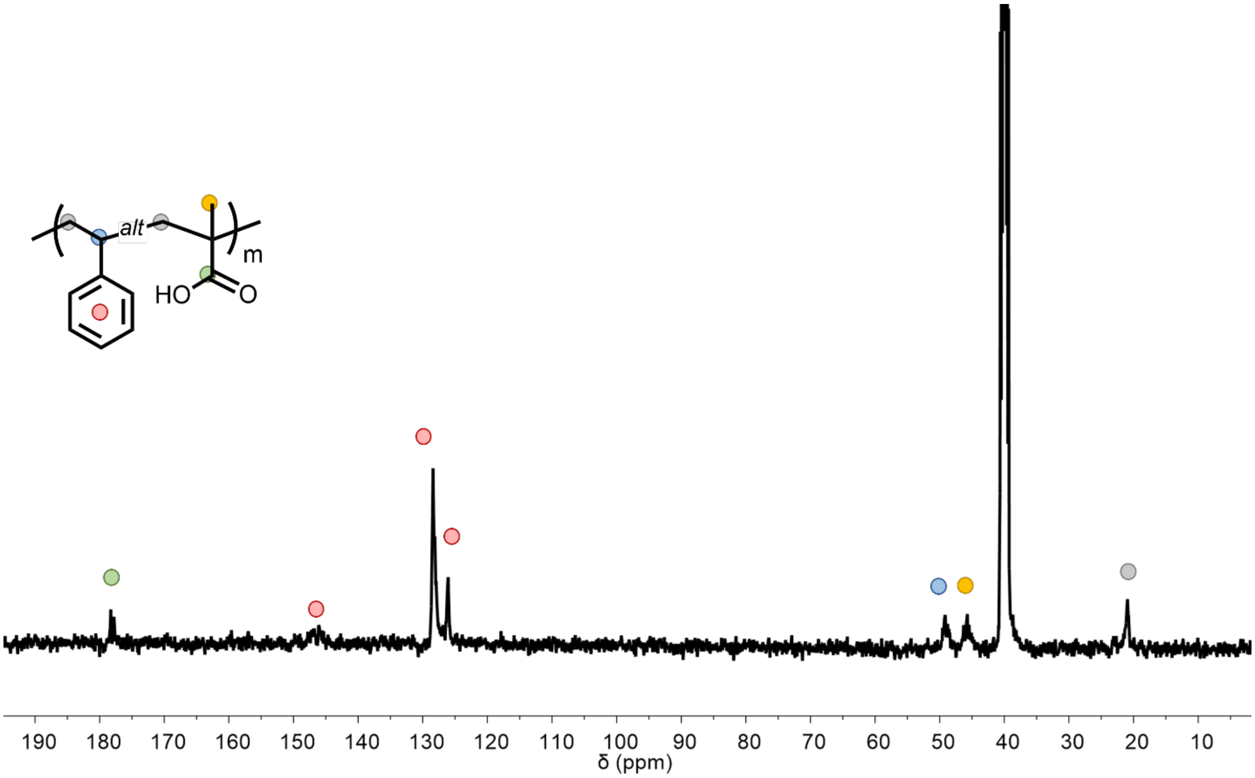

Figure S32. *^{13}C NMR spectrum of P(Sty-alt-MA) in DMSO-d_6.*

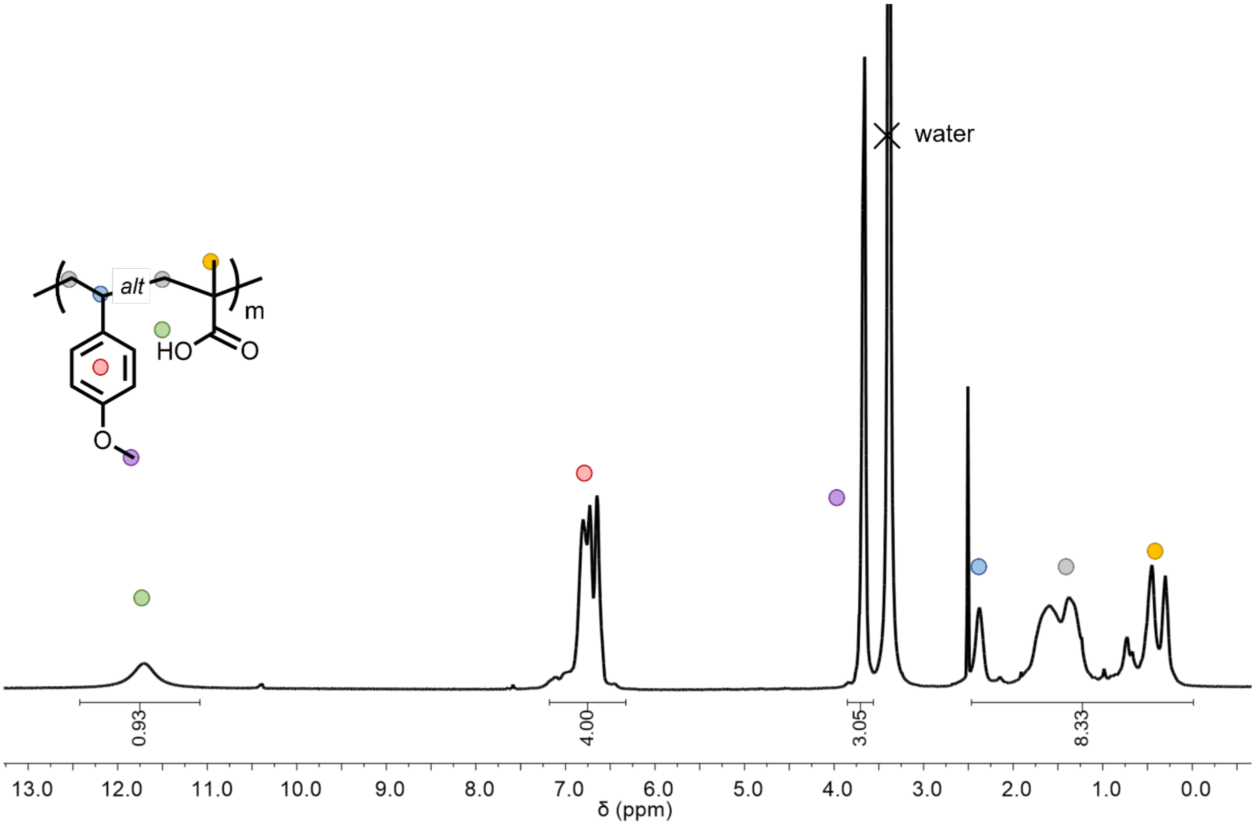

Figure S33. *^{1}H NMR spectrum of P(StyOMe-alt-MA) in DMSO-d_6.*

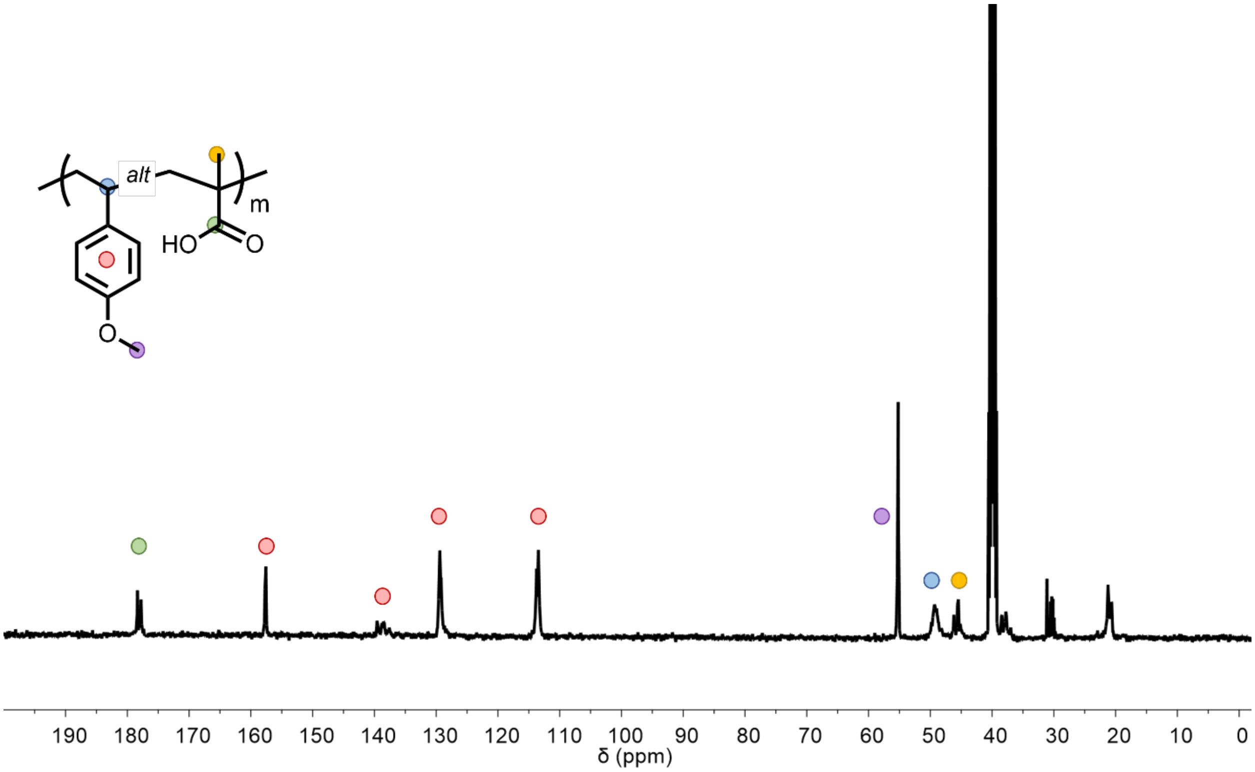

Figure S34. *^{13}C NMR spectrum of P(StyOMe-alt-MA) in DMSO-d$_6$.*

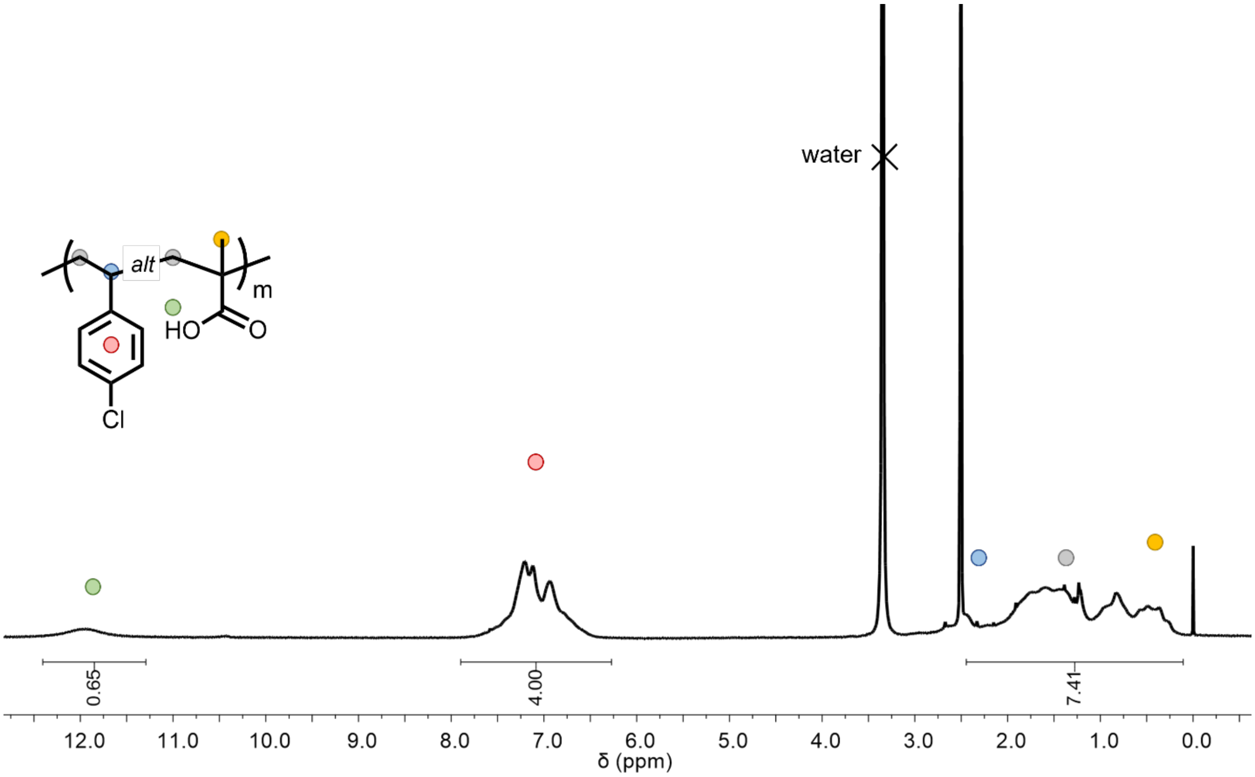

Figure S35. *^{1}H NMR spectrum of P(StyCl-alt-MA) in DMSO-d$_6$.*

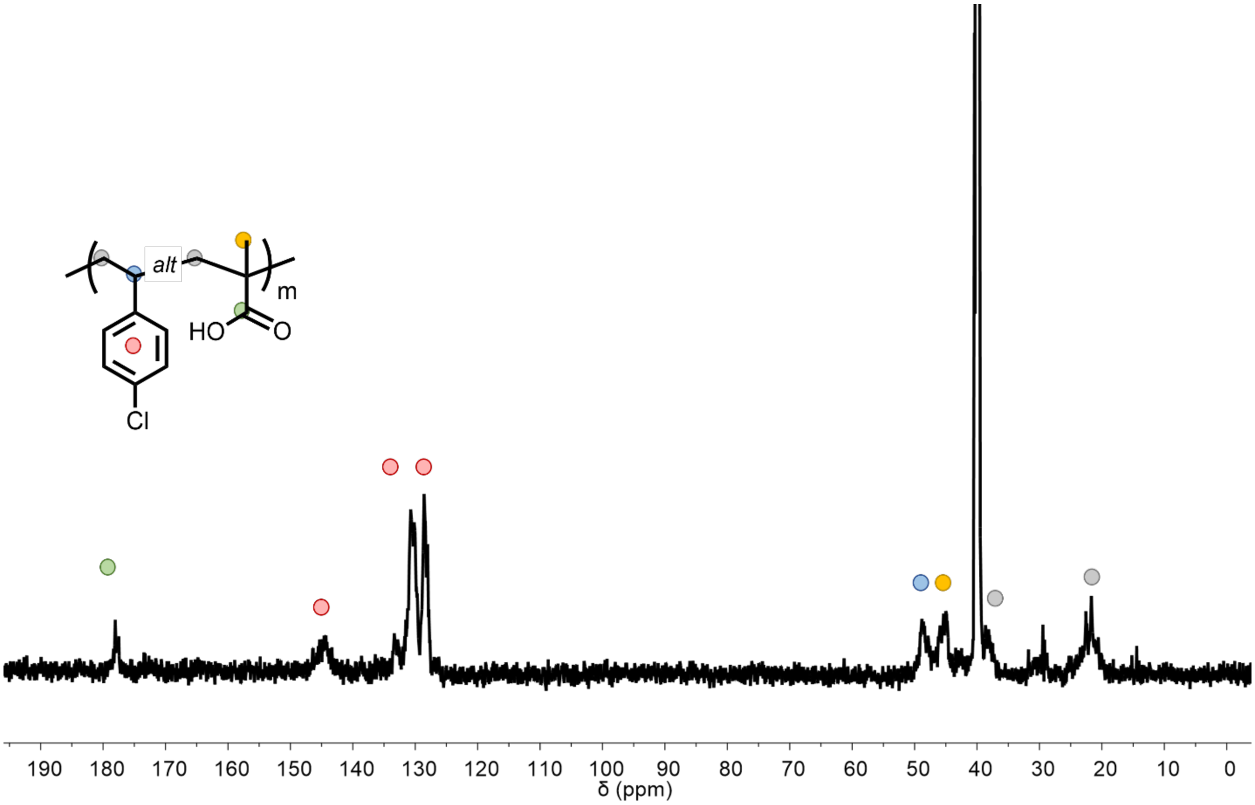

Figure S36. *^{13}C NMR spectrum of P(StyCl-alt-MA) in DMSO-d$_6$.*

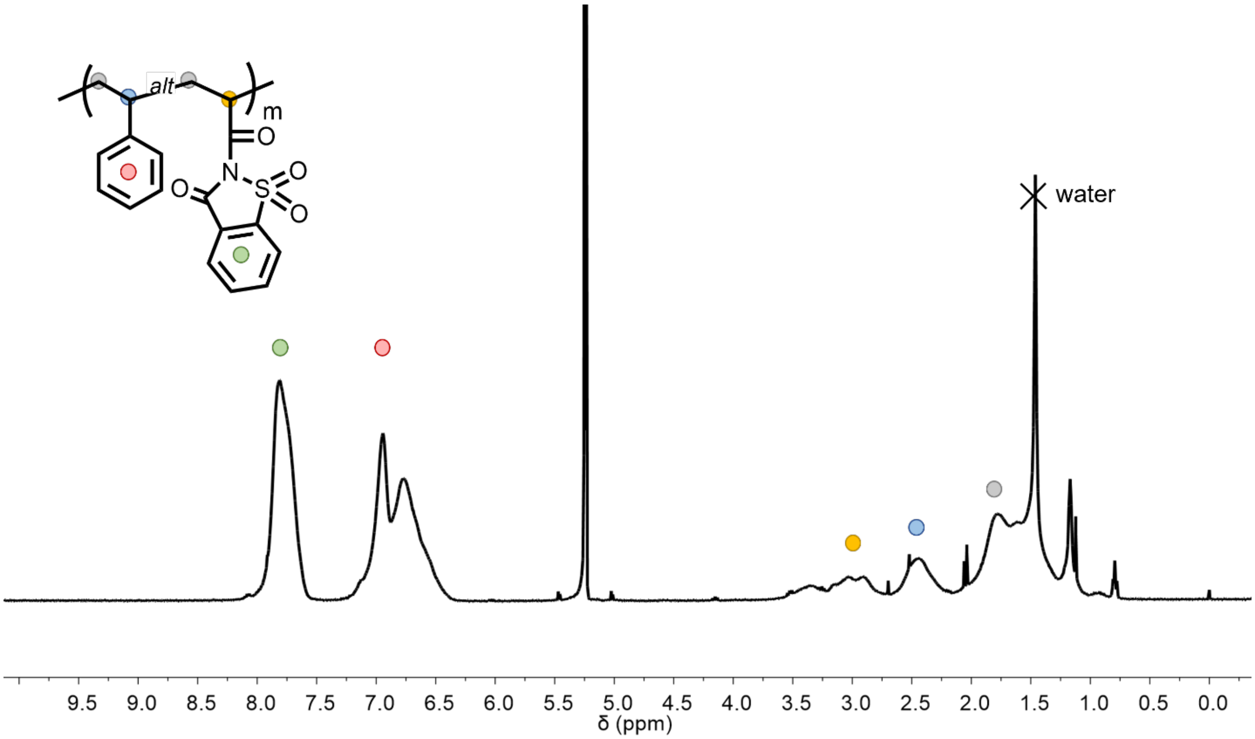

Figure S37. *^{1}H NMR spectrum of P(Sty-alt-SacchA) in CD$_2$Cl$_2$.*

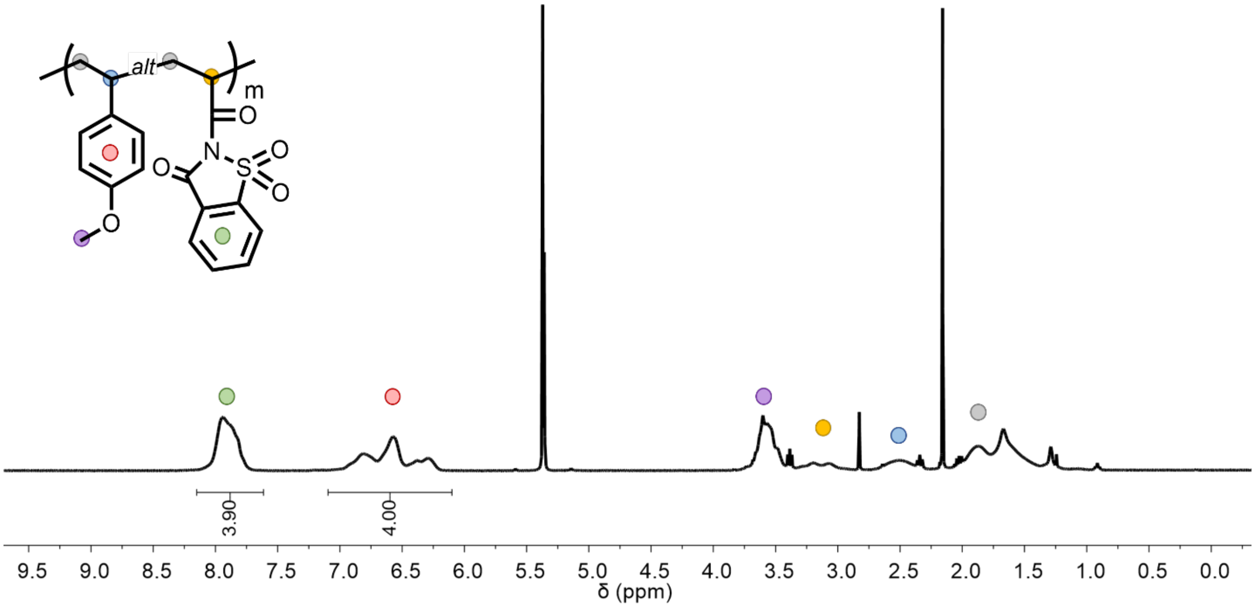

Figure S38. *^{1}H NMR spectrum of P(StyOMe-alt-SacchA) in CD_2Cl_2.*

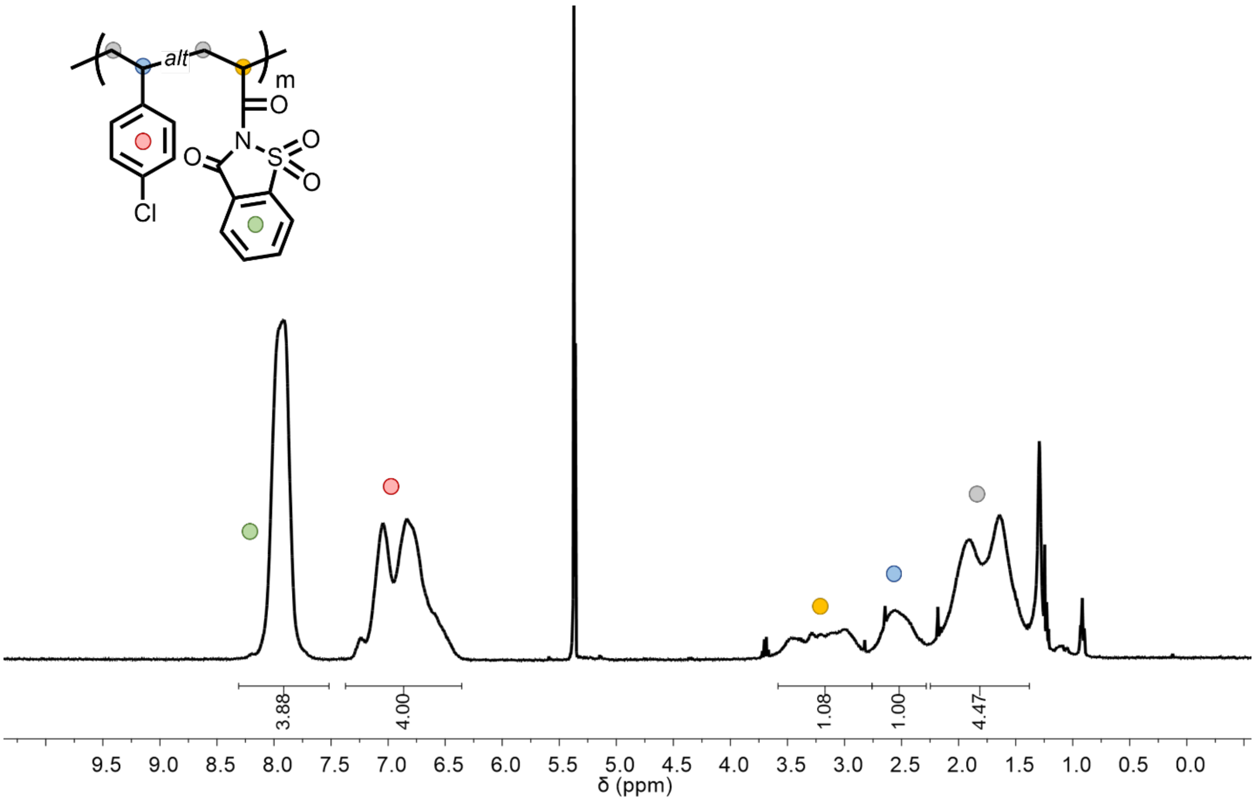

Figure S39. *^{1}H NMR spectrum of P(StyCl-alt-SacchA) in CD_2Cl_2.*

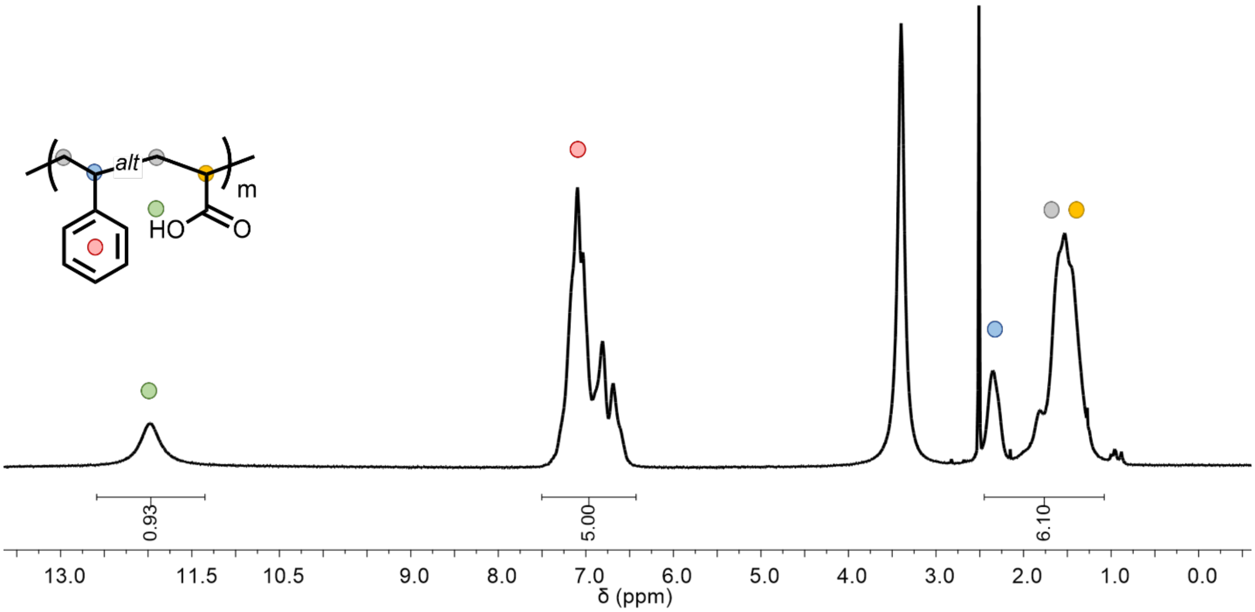

Figure S40. *^{1}H NMR spectrum of P(Sty-alt-AA) in DMSO-d$_6$.*

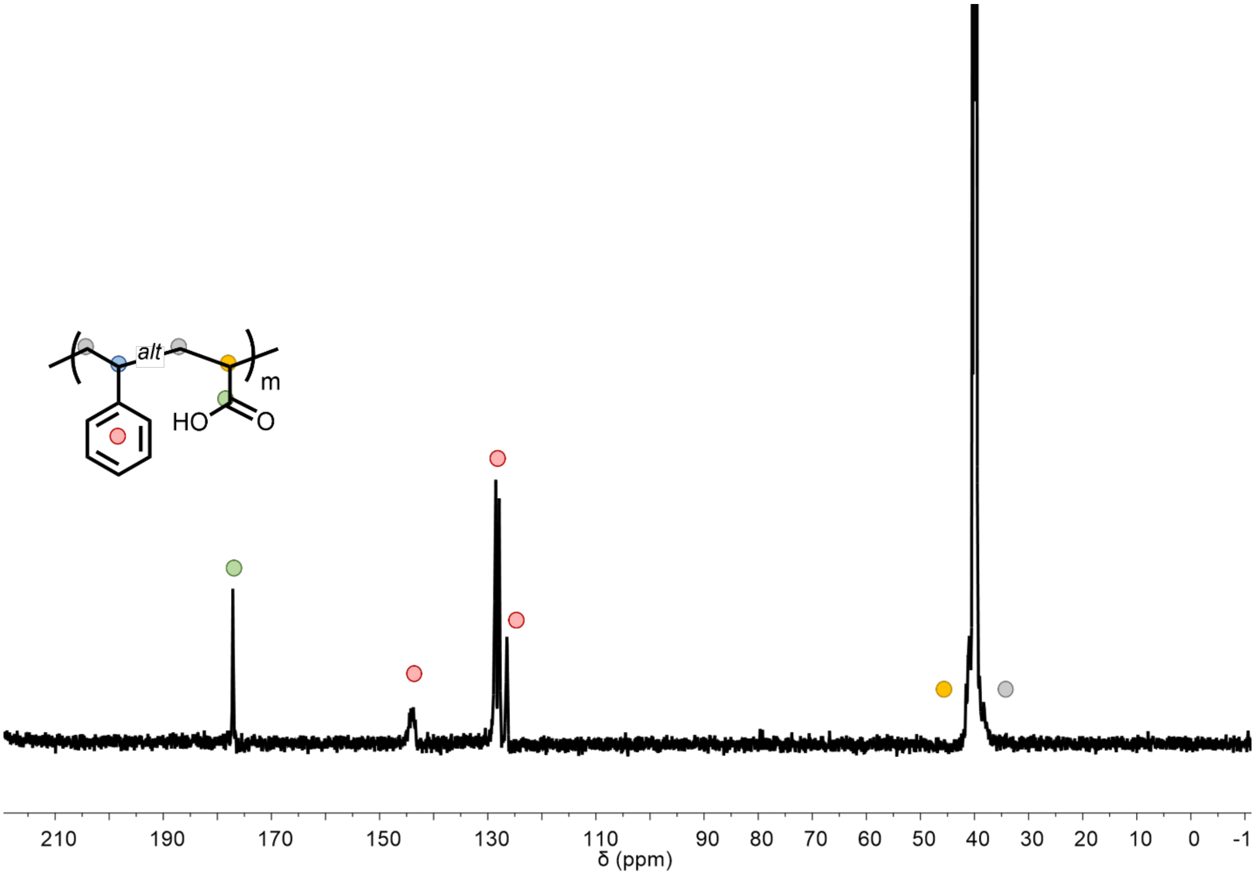

Figure S41. *^{13}C NMR spectrum of P(Sty-alt-AA) in DMSO-d$_6$.*

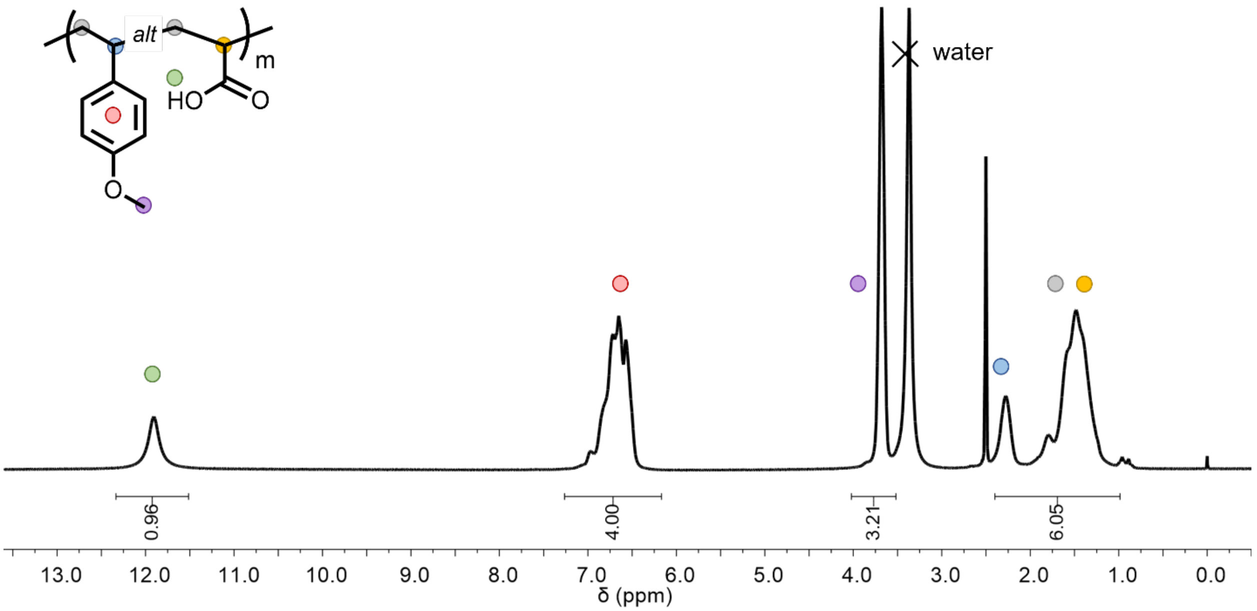

Figure S42. *^{1}H NMR spectrum of P(StyOMe-alt-AA) in DMSO-d_6.*

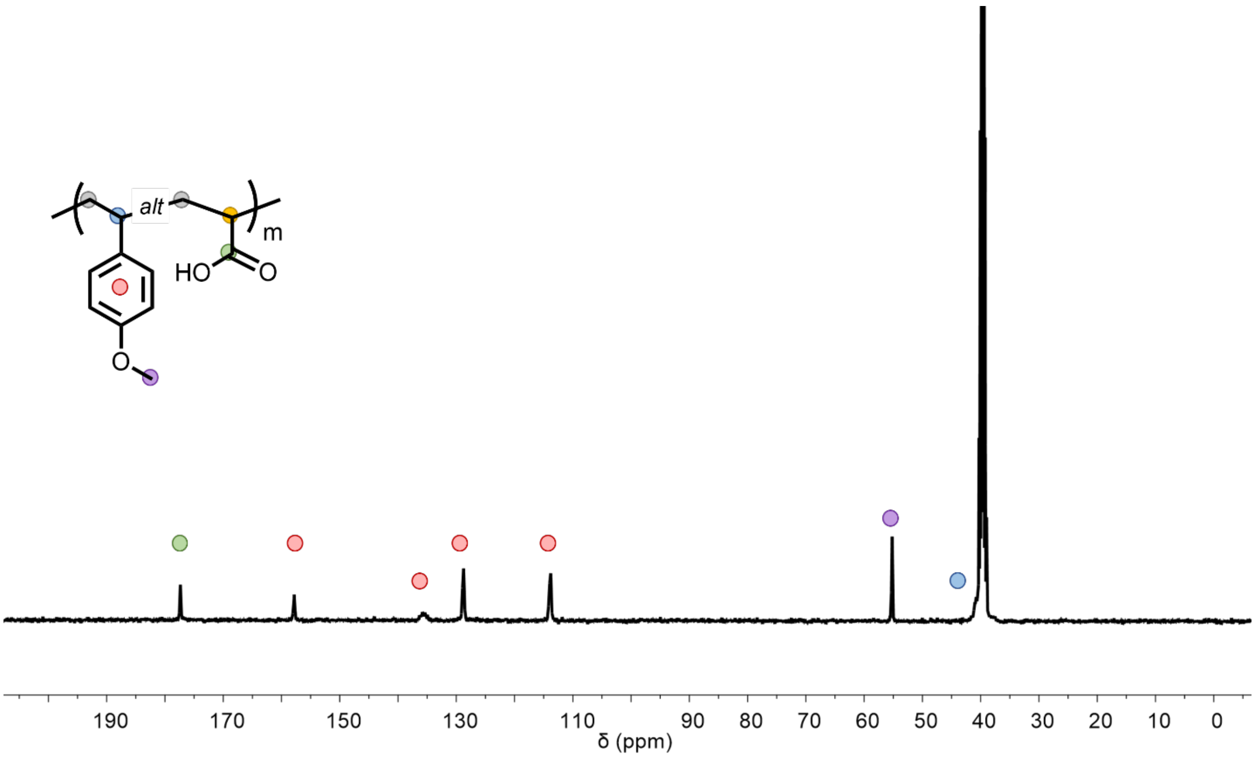

Figure S43. *^{13}C NMR spectrum of P(StyOMe-alt-AA) in DMSO-d_6.*

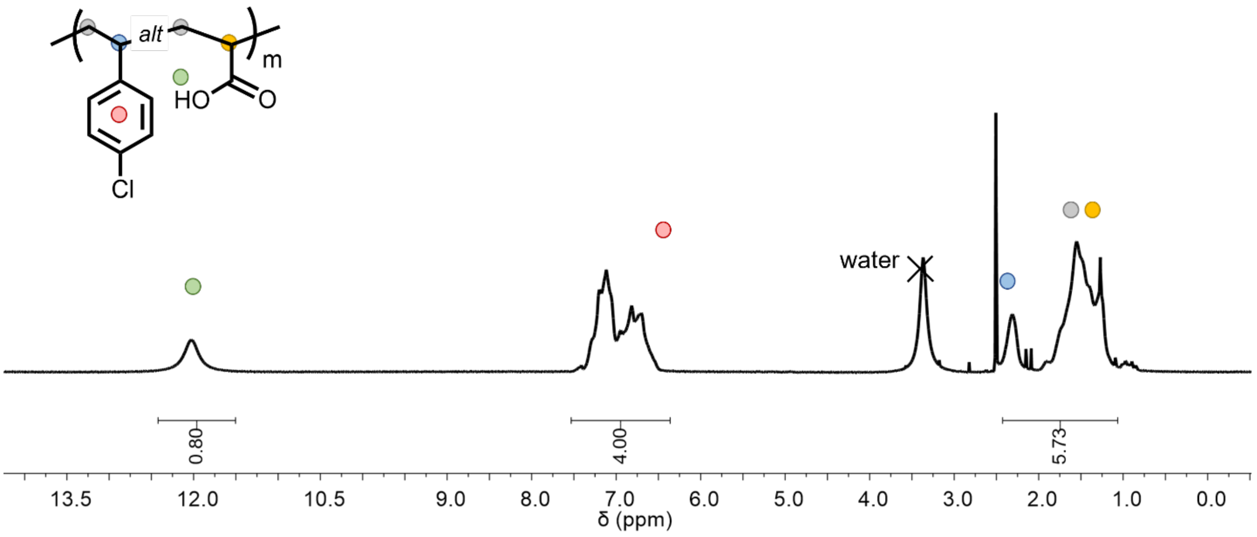

Figure S44. *^{1}H NMR spectrum of P(StyCl-alt-AA) in DMSO-d_6.*

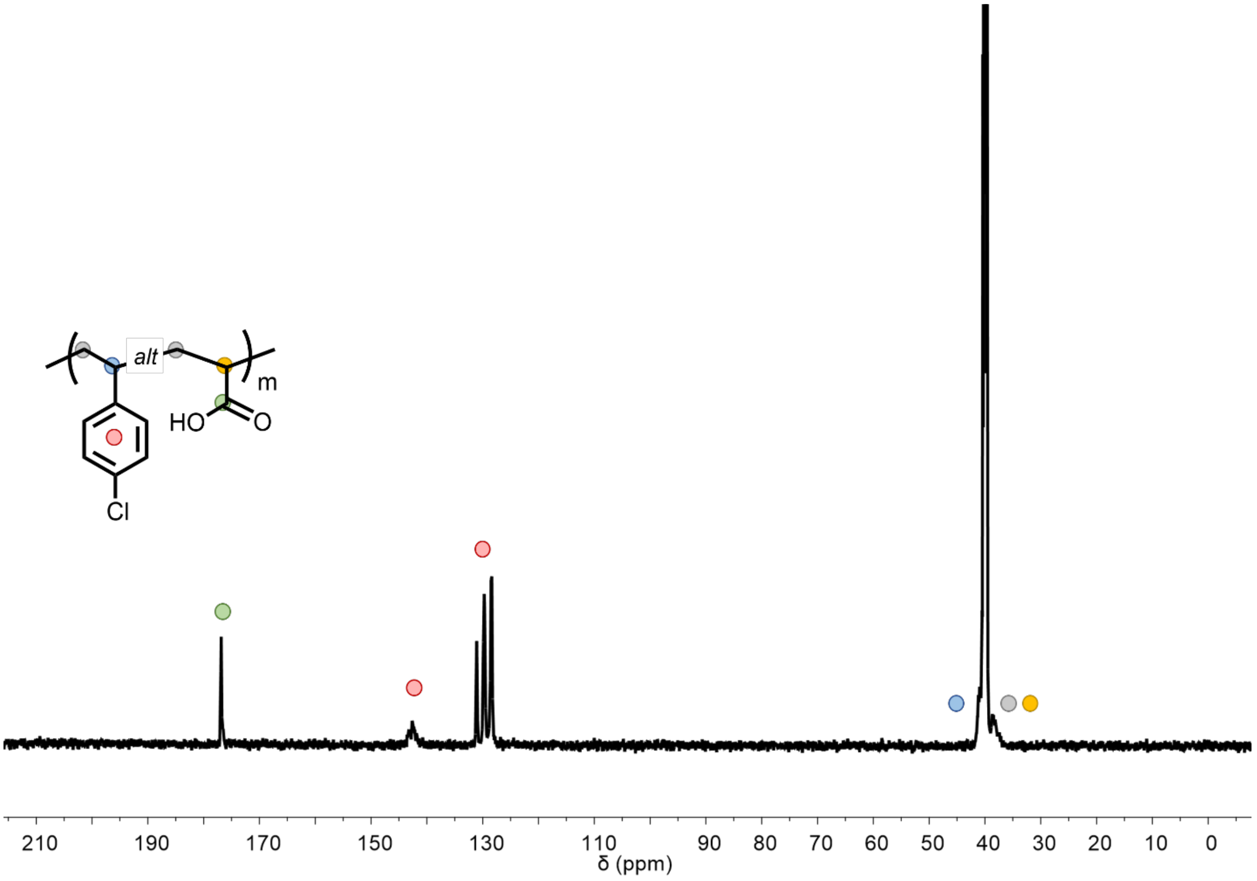

Figure S45. *^{13}C NMR spectrum of P(StyCl-alt-AA) in DMSO-d_6.*

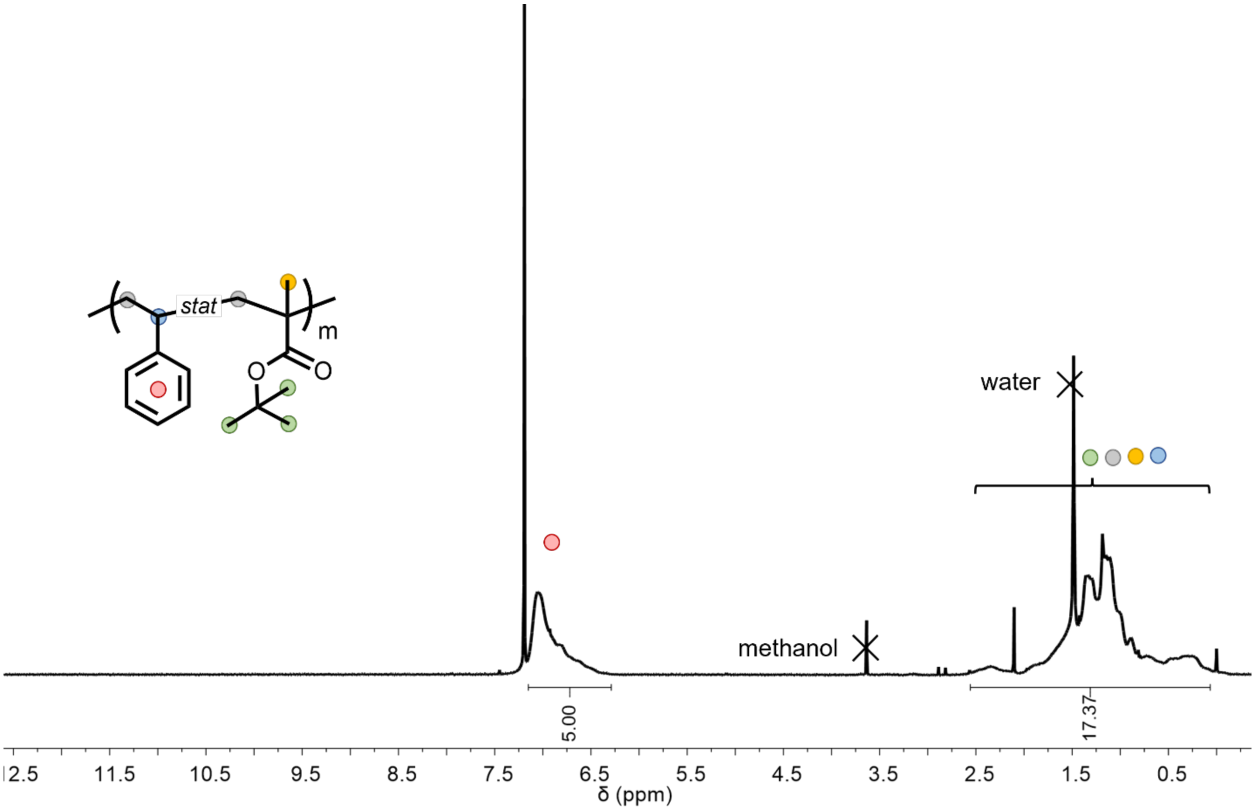

Figure S46. *^{1}H NMR spectrum of P(Sty$_{0.54}$-stat-tertButMA$_{0.46}$) in CDCl$_3$.*

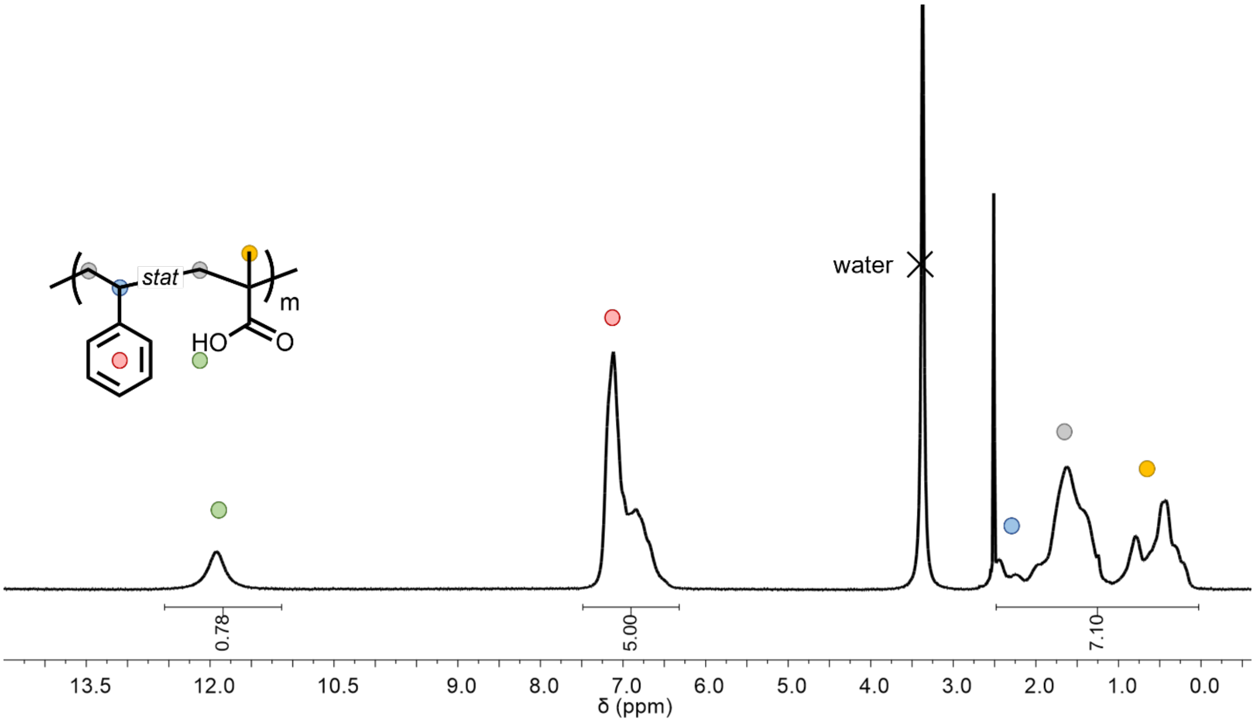

Figure S47. *^{1}H NMR spectrum of P(Sty$_{0.54}$-stat-MA$_{0.46}$) in DMSO-d$_6$.*

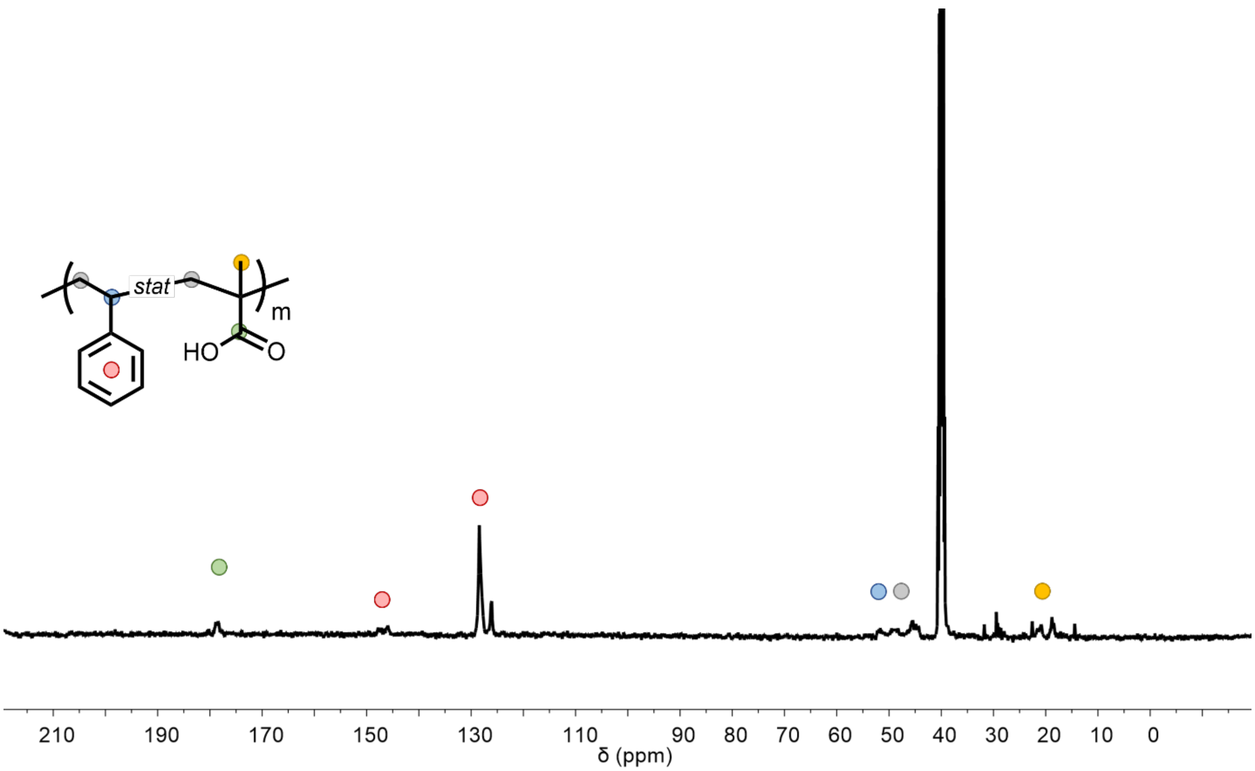

Figure S48. *^{13}C NMR spectrum of P(Sty$_{0.54}$-stat-MA$_{0.46}$) in DMSO-d$_6$.*

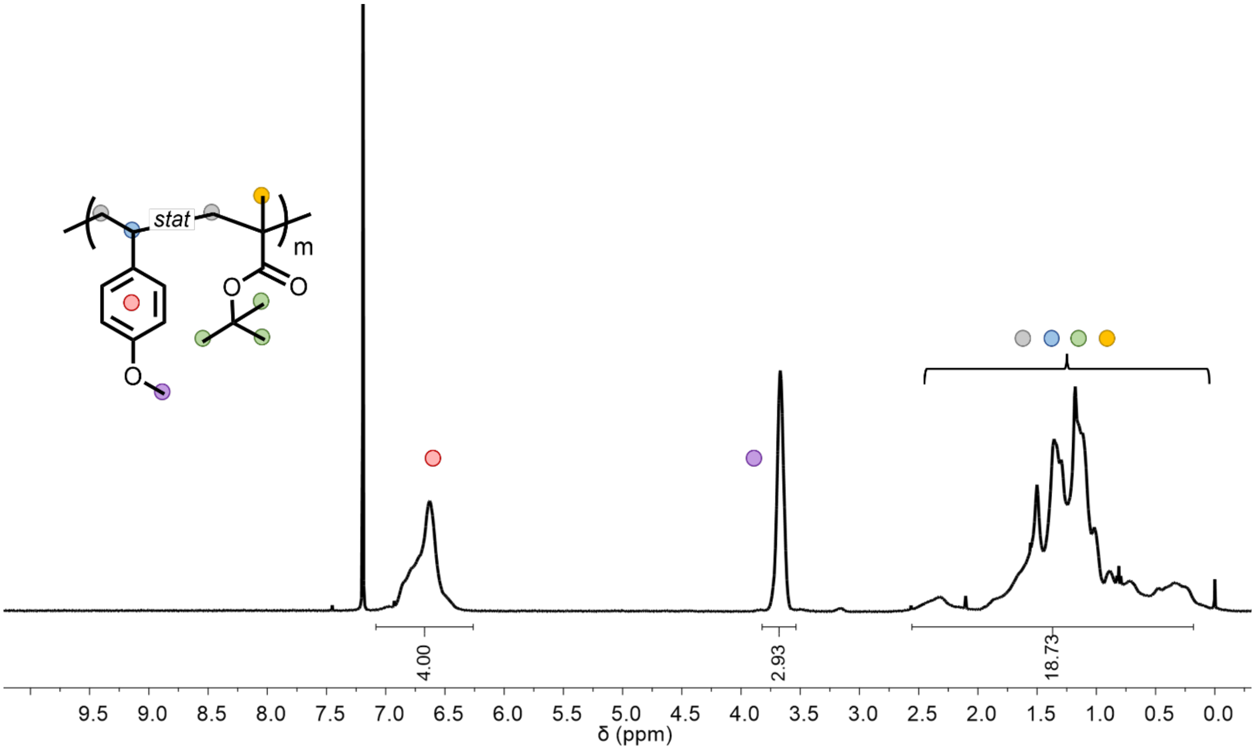

Figure S49. *^{1}H NMR spectrum of P(StyOMe$_{0.51}$-stat-tertButMA$_{0.49}$) in CDCl$_3$.*

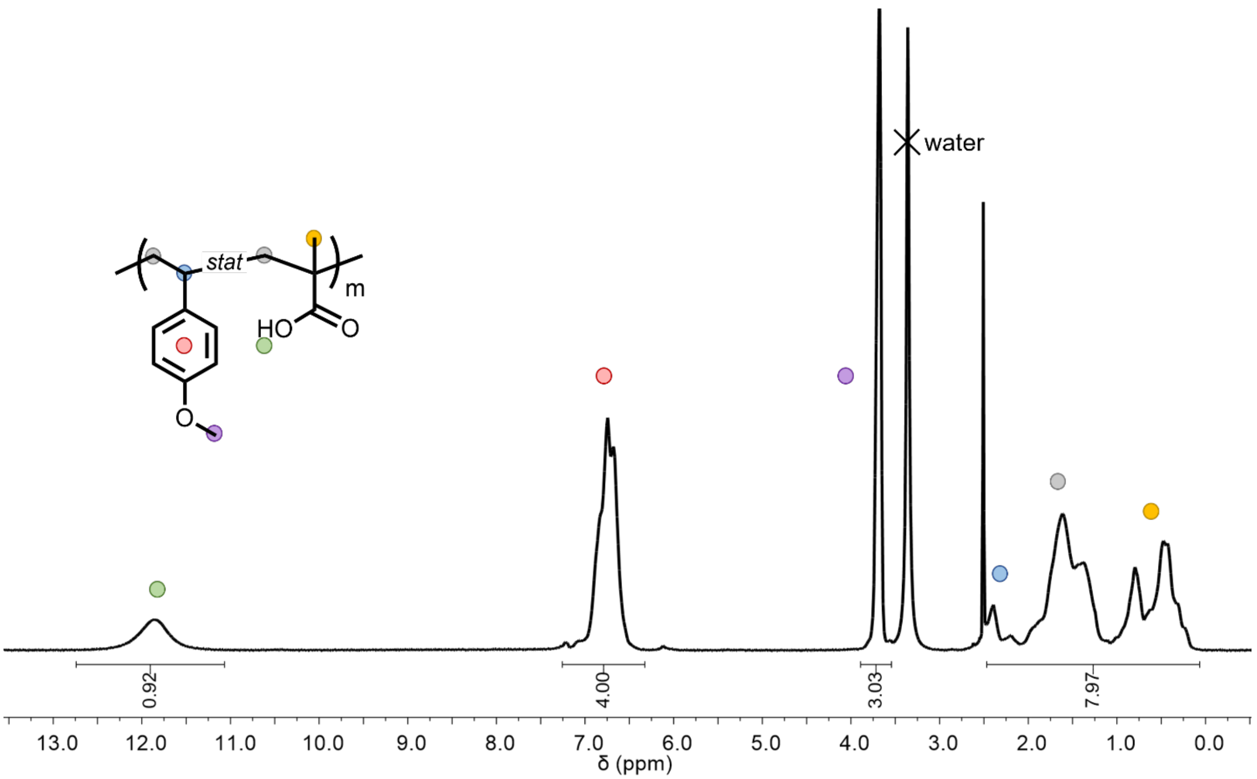

Figure S50. *^{1}H NMR spectrum of P(StyOMe$_{0.51}$-stat-MA$_{0.49}$) in DMSO-d$_6$.*

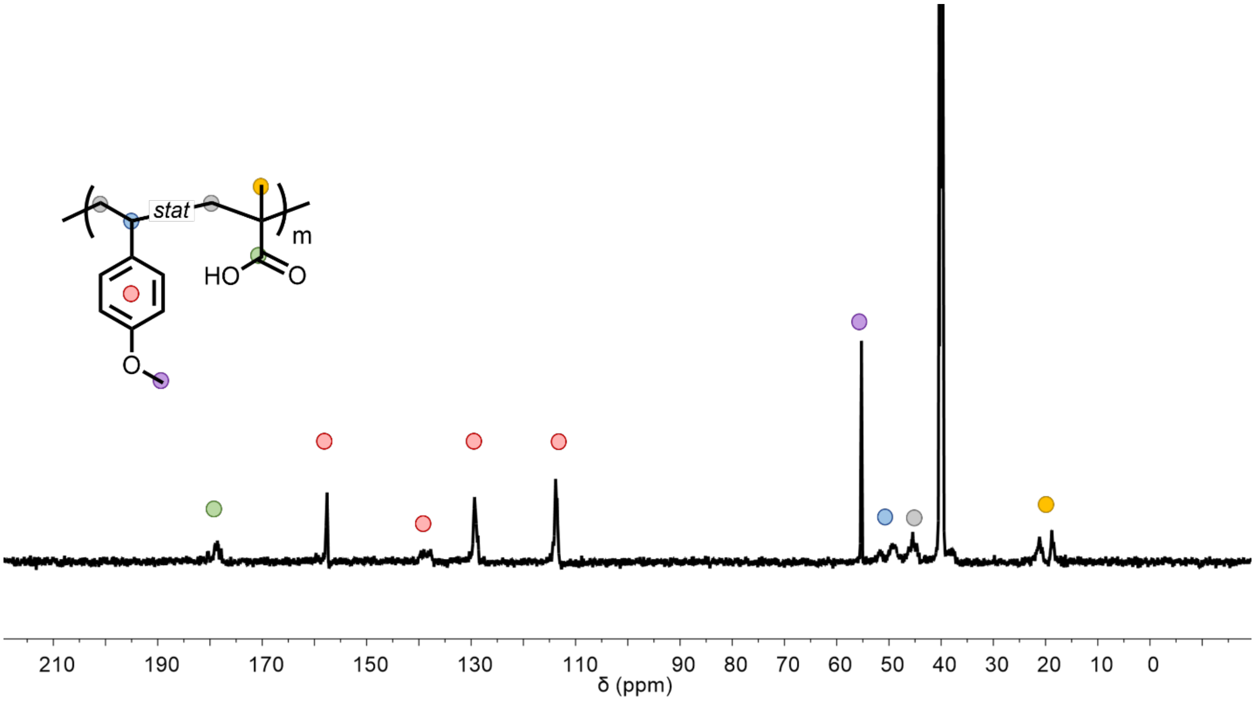

Figure S51. *^{13}C NMR spectrum of P(StyOMe$_{0.51}$-stat-MA$_{0.49}$) in DMSO-d$_6$.*

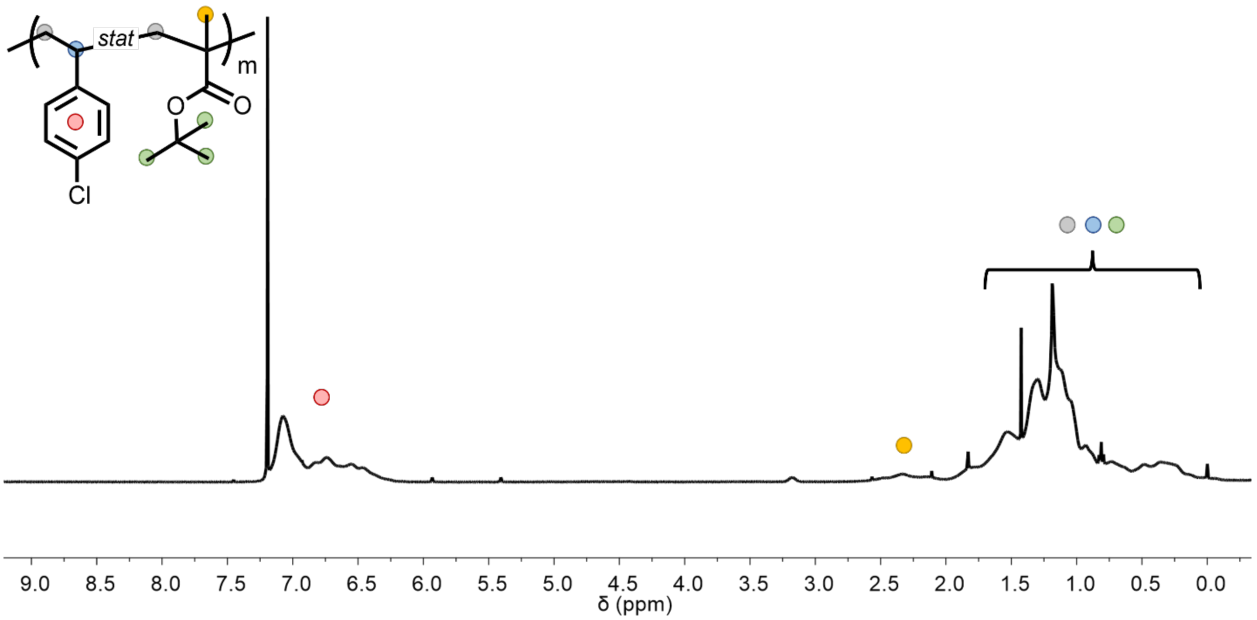

Figure S52. *^{1}H NMR spectrum of P(StyCl$_{0.47}$-stat-tertBuMA$_{0.53}$) in CDCl$_3$.*

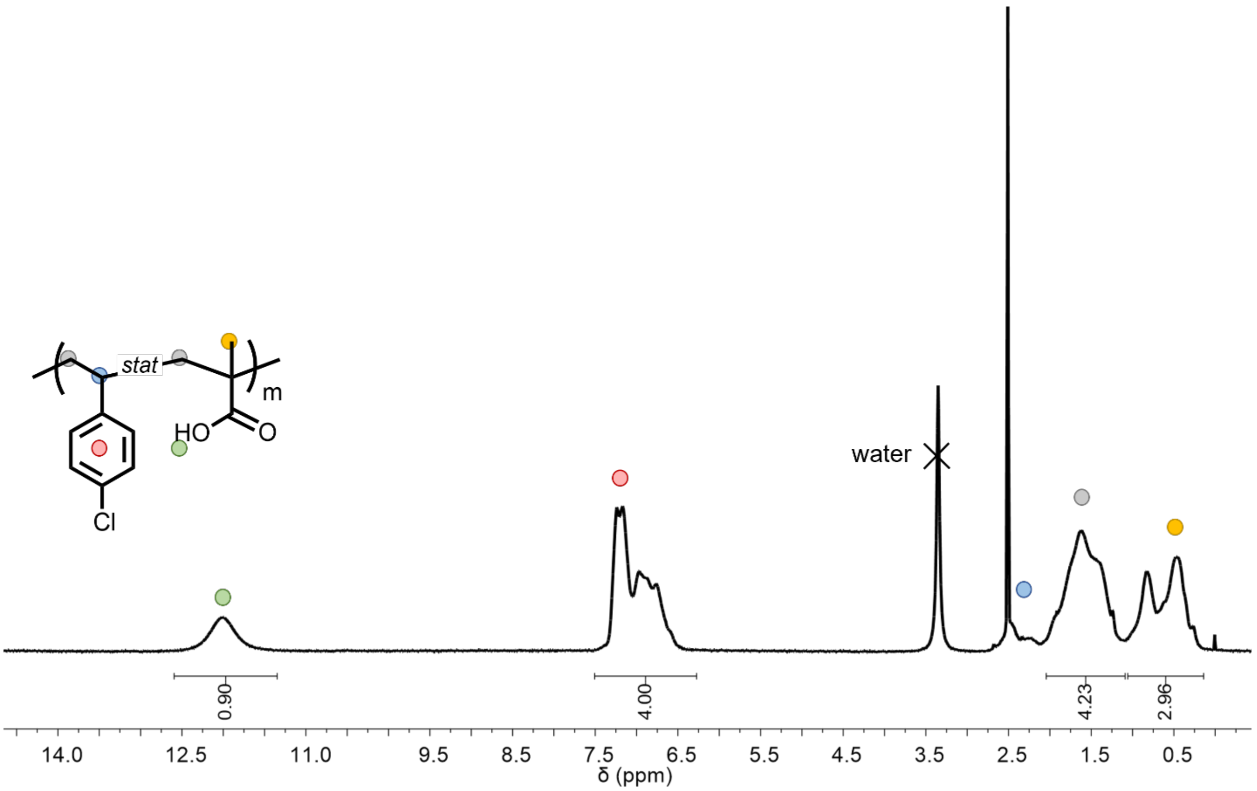

Figure S53. *^{1}H NMR spectrum of P(StyCl$_{0.47}$-stat-MA$_{0.53}$) in DMSO-d$_6$.*

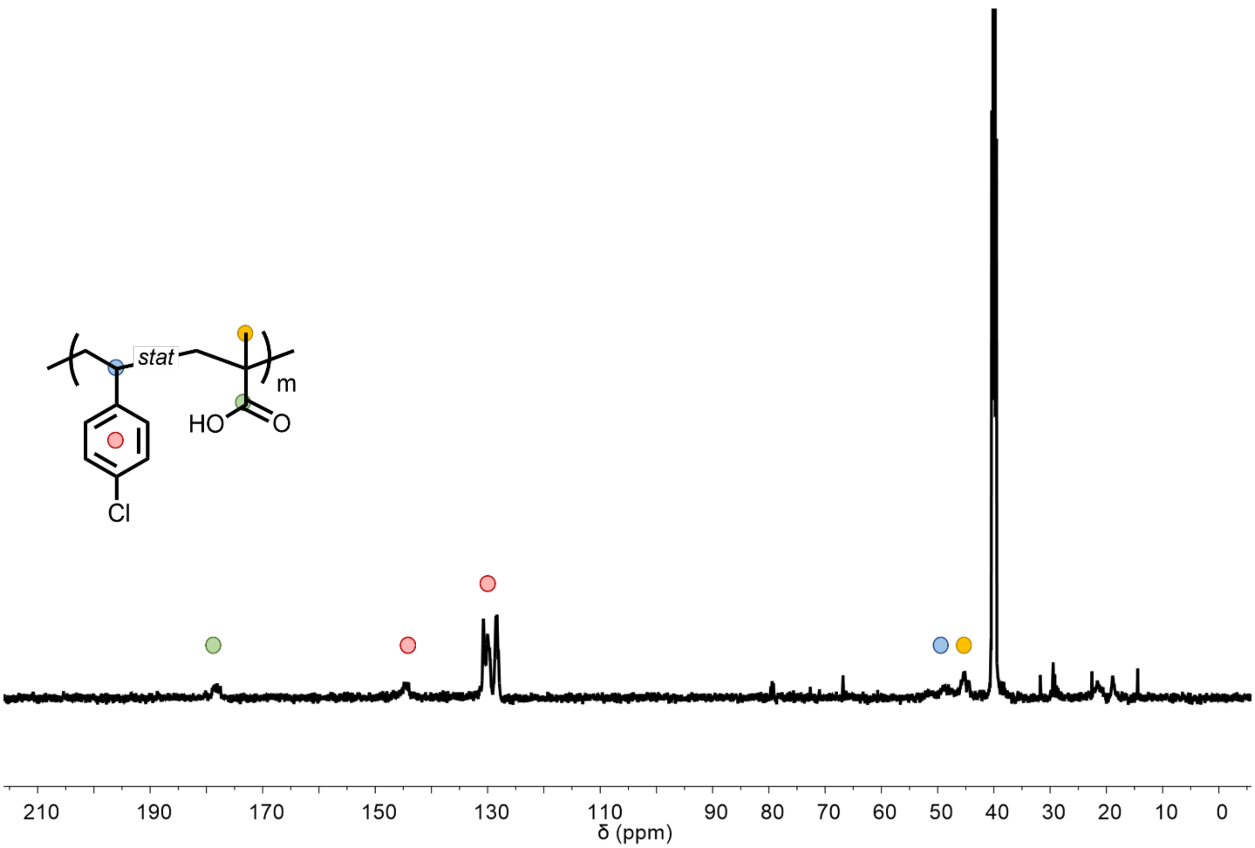

Figure S54. *^{13}C NMR spectrum of P(StyCl$_{0.47}$-stat-MA$_{0.53}$) in DMSO-d$_6$.*

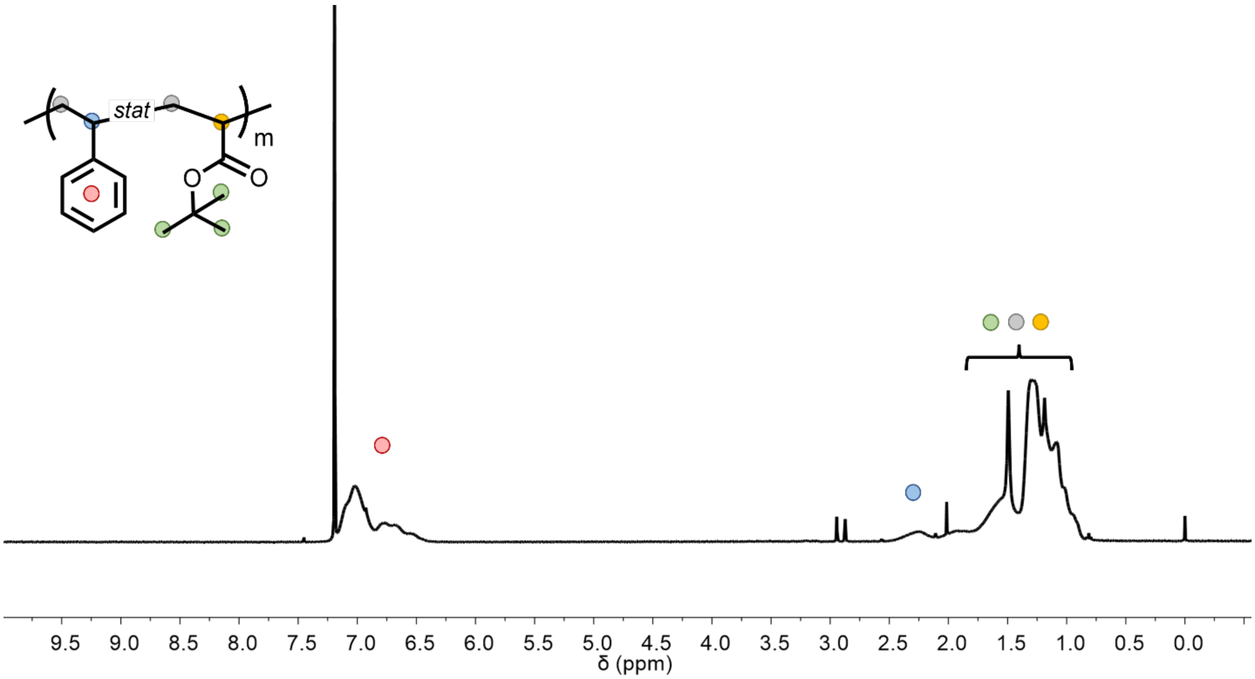

Figure S55. *^{1}H NMR spectrum of P(Sty$_{0.47}$-stat-tertBuA$_{0.53}$) in CDCl$_3$.*

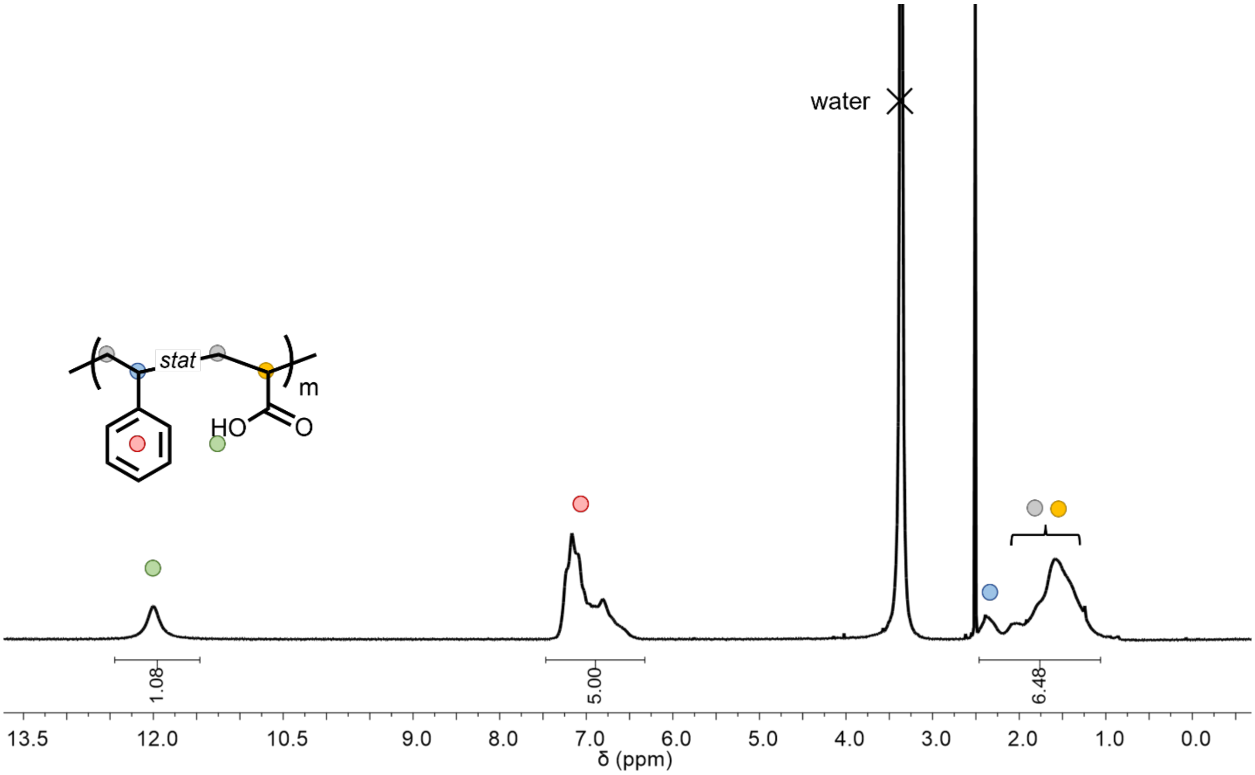

Figure S56. *^{1}H NMR spectrum of P(Sty$_{0.47}$-stat-AA$_{0.53}$) in DMSO-d$_6$.*

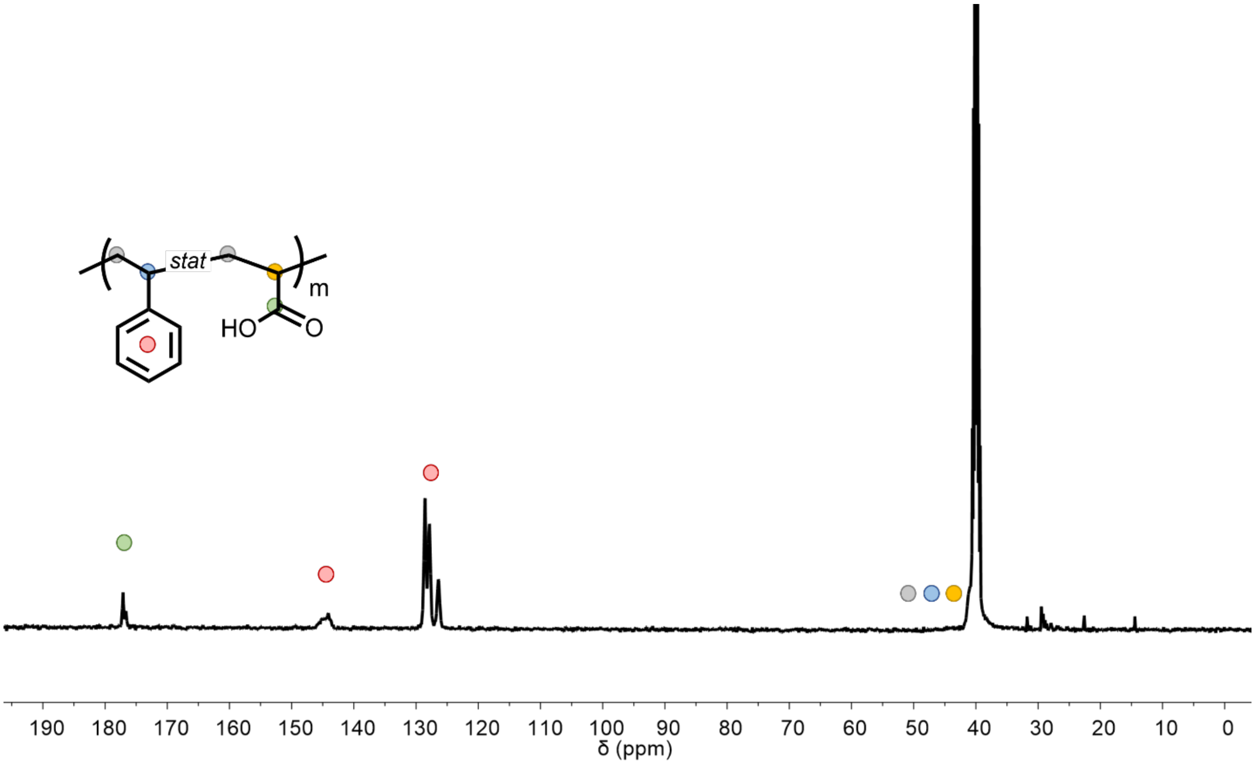

Figure S57. *^{13}C NMR spectrum of P(Sty$_{0.47}$-stat-AA$_{0.53}$) in DMSO-d$_6$.*

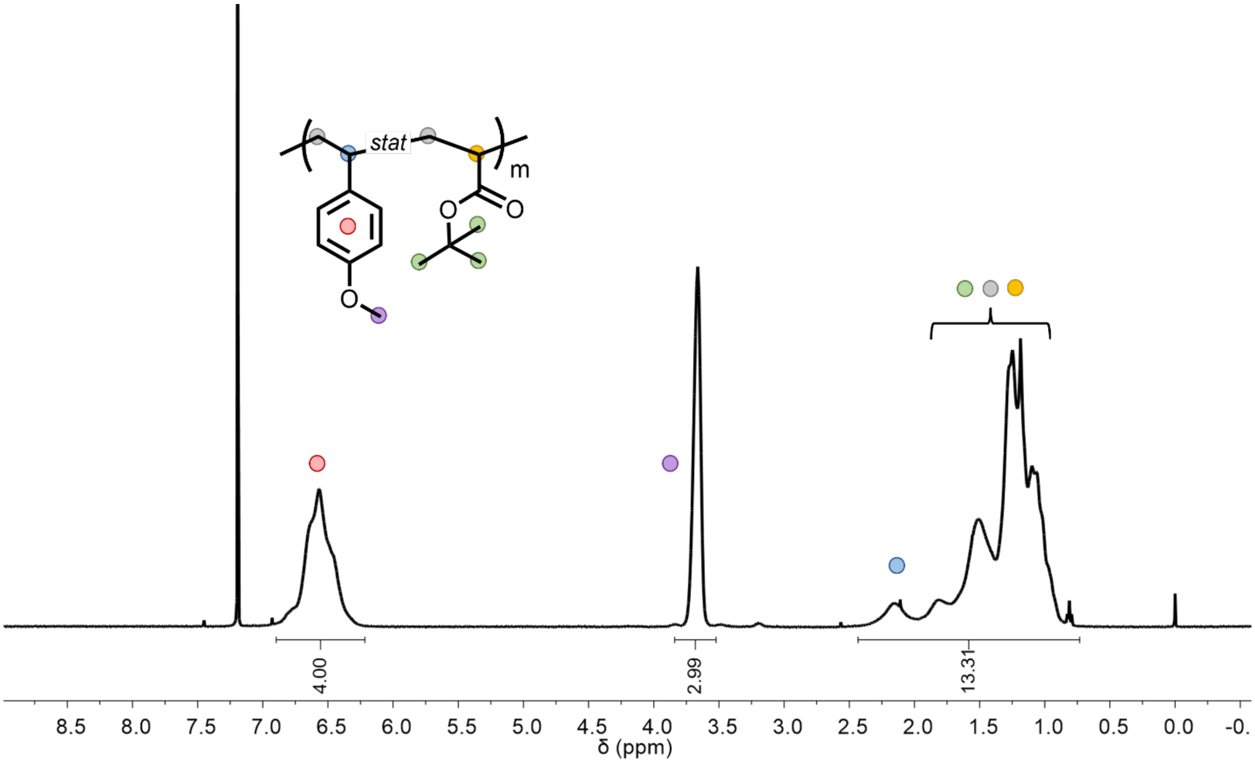

Figure S58. *^{1}H NMR spectrum of P(Sty$_{0.54}$-stat-tertButA$_{0.46}$) in $CDCl_3$.*

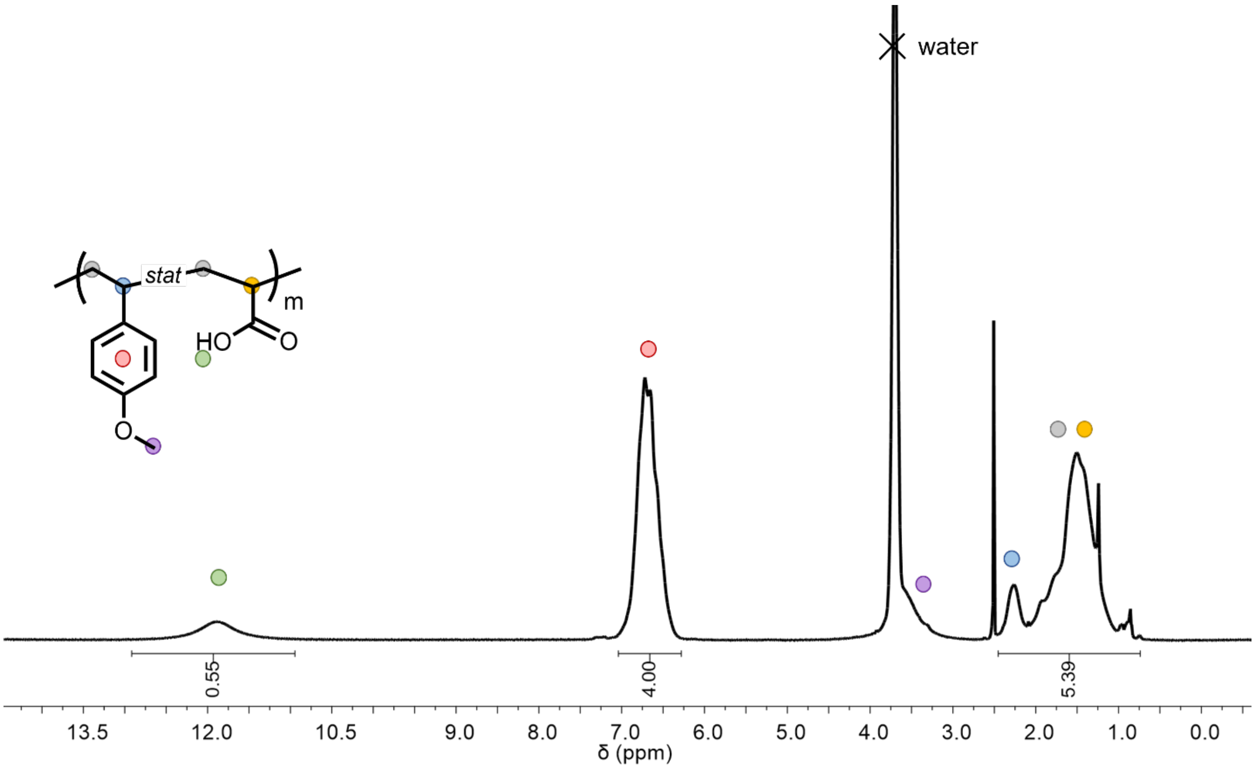

Figure S59. *^{1}H NMR spectrum of P(StyOMe$_{0.54}$-stat-AA$_{0.46}$) in DMSO-d_6.*

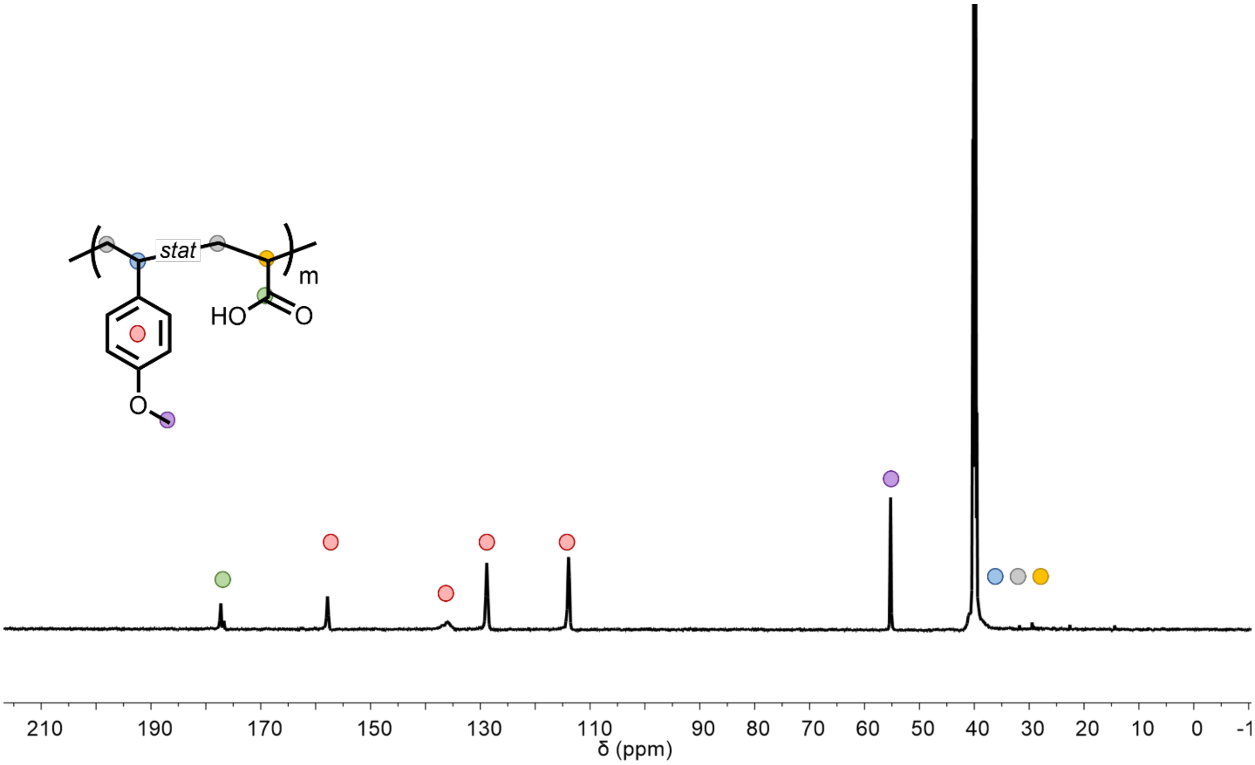

Figure S60. *^{13}C NMR spectrum of P(StyOMe$_{0.54}$-stat-AA$_{0.46}$) in DMSO-d$_6$.*

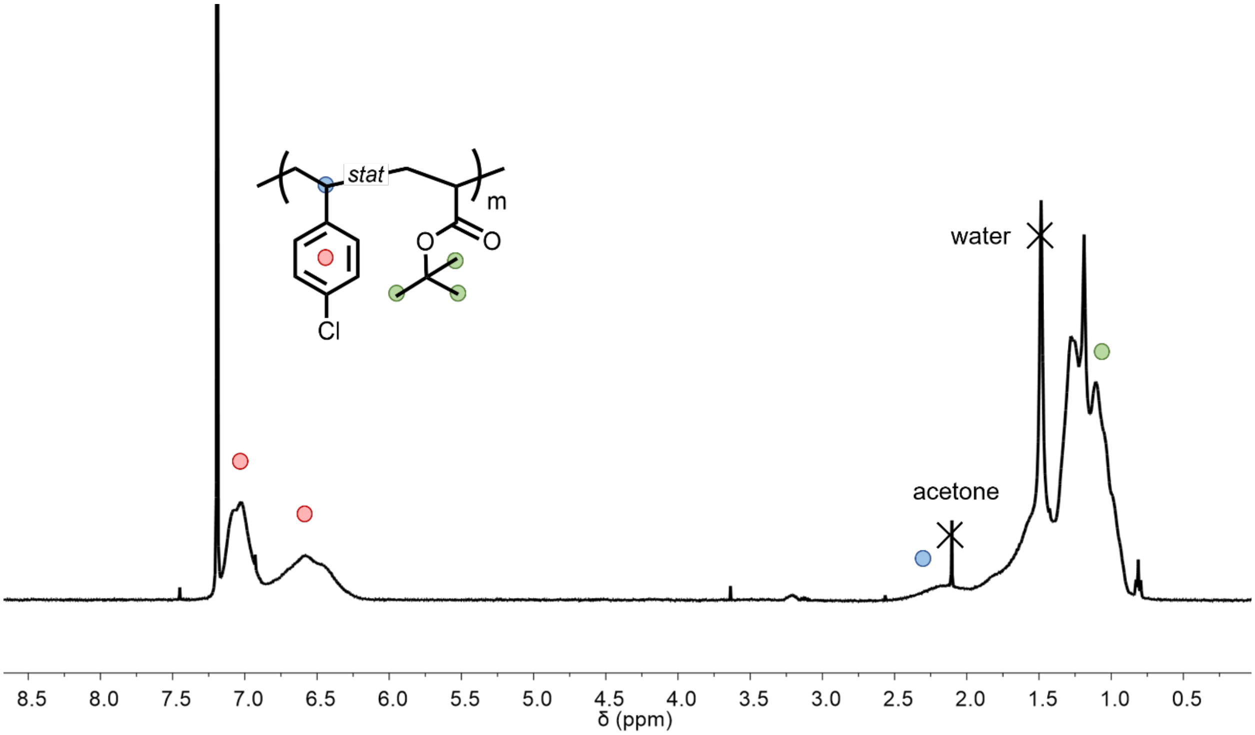

Figure S61. *^{1}H NMR spectrum of P(StyCl$_{0.49}$-stat-tertButA$_{0.51}$) in CDCl$_3$.*

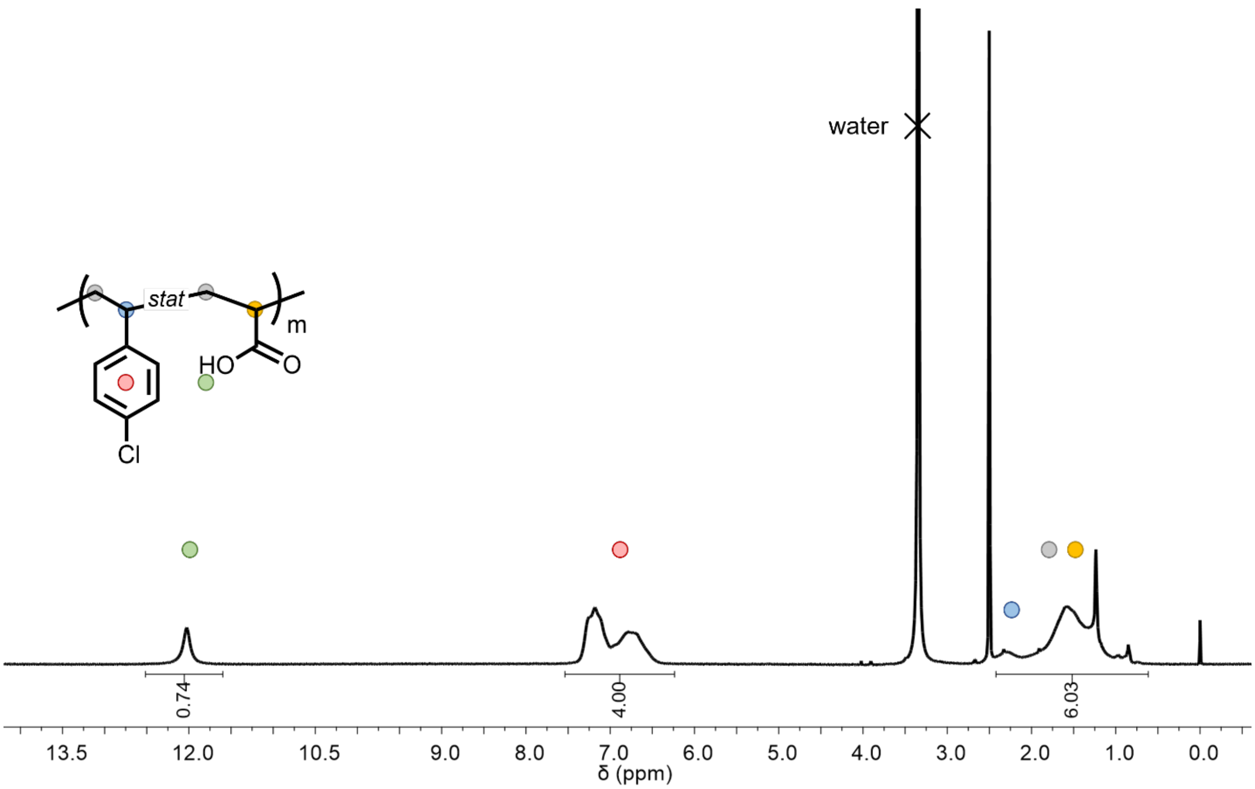

Figure S62. *^{1}H NMR spectrum of P(StyCl$_{0.49}$-stat-AA$_{0.51}$) in DMSO-d$_6$.*

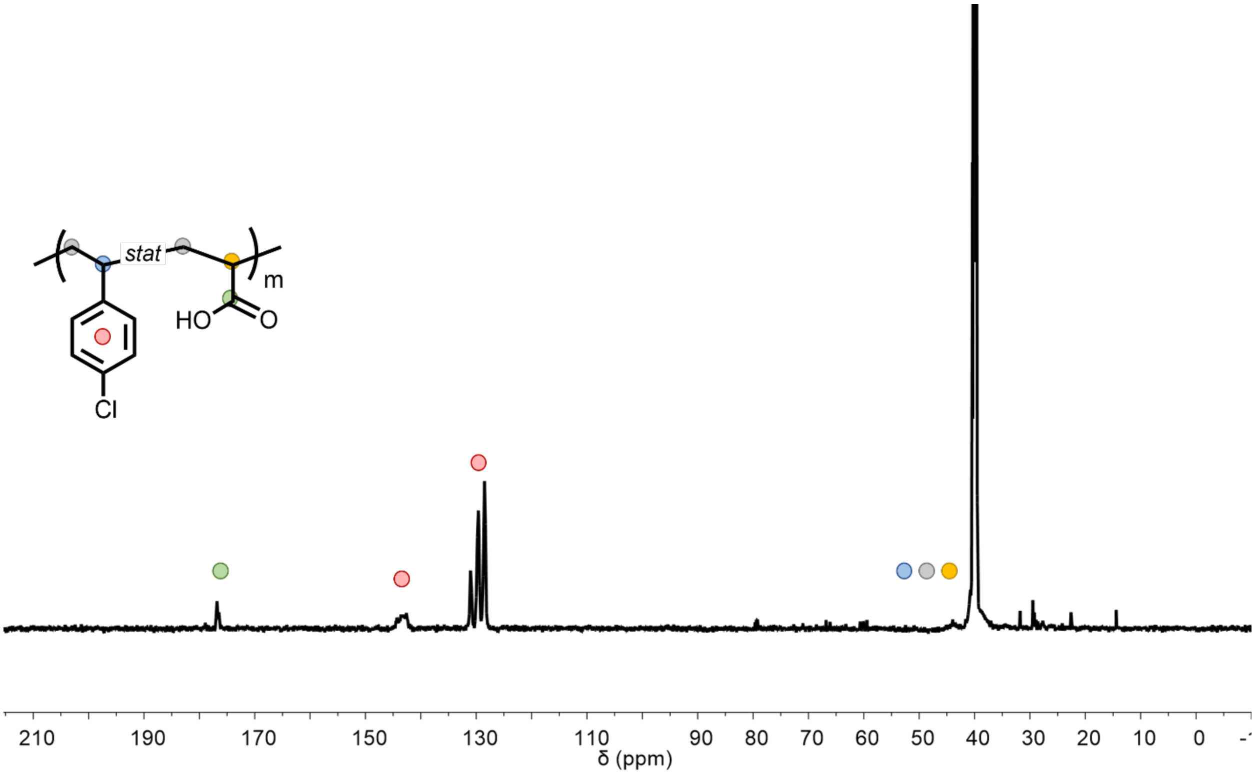

Figure S63. *^{13}C NMR spectrum of P(StyCl$_{0.49}$-stat-AA$_{0.51}$) in DMSO-d$_6$.*

Decarboxylated copolymers

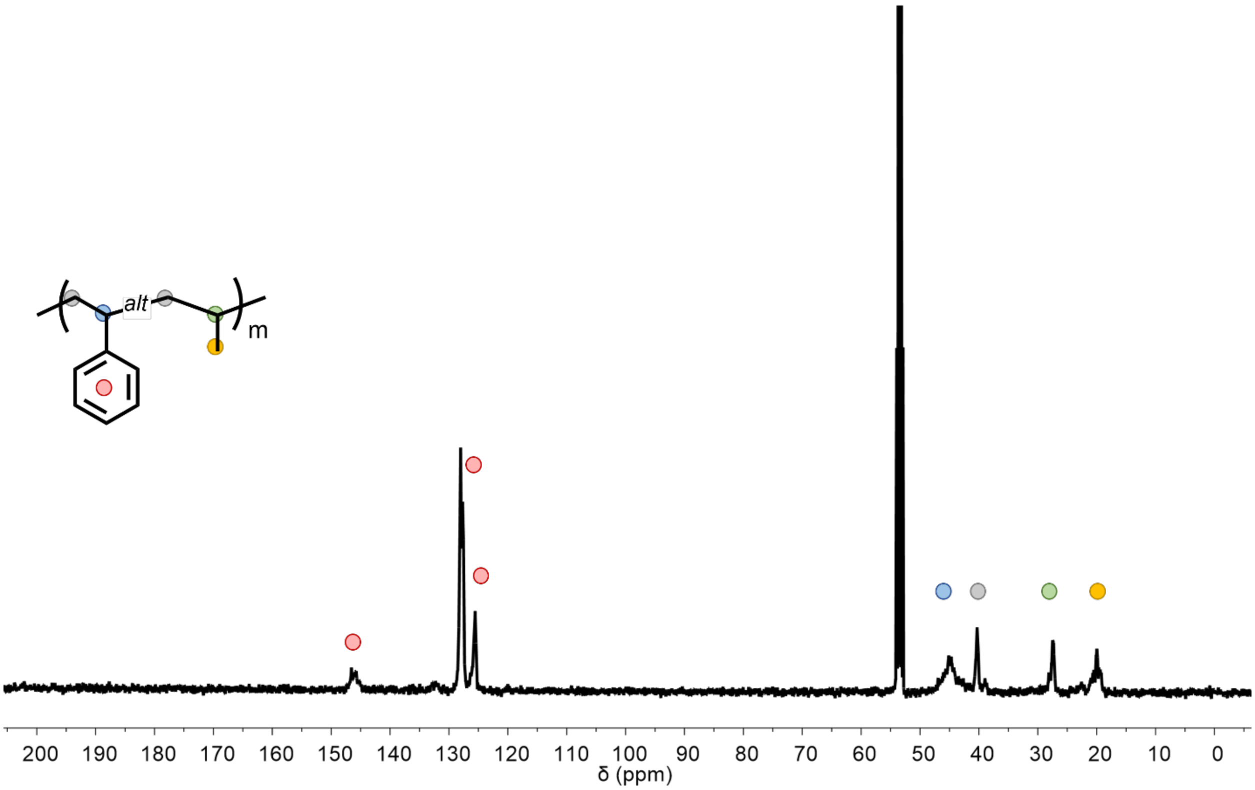

Figure S64. *^{13}C NMR spectrum of P(Sty-alt-P) in CD_2Cl_2.*

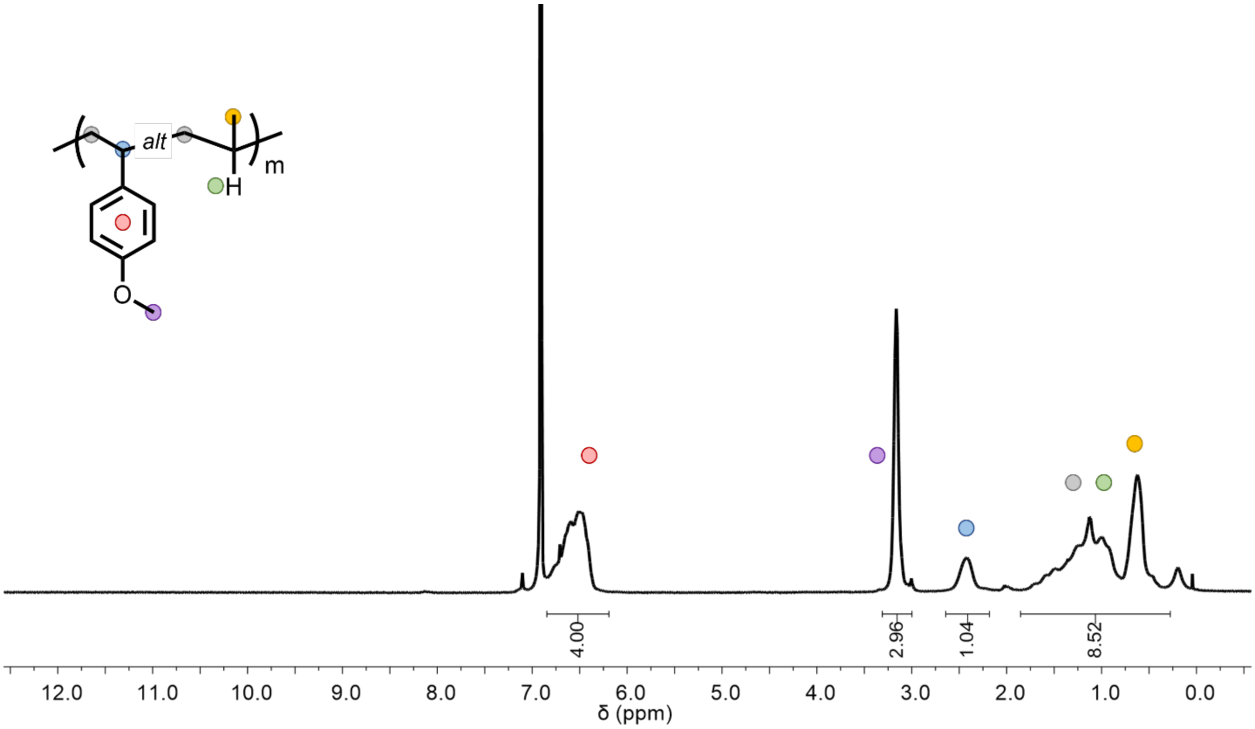

Figure S65. *^{1}H NMR spectrum of P(StyOMe-alt-P) in benzene-d_6.*

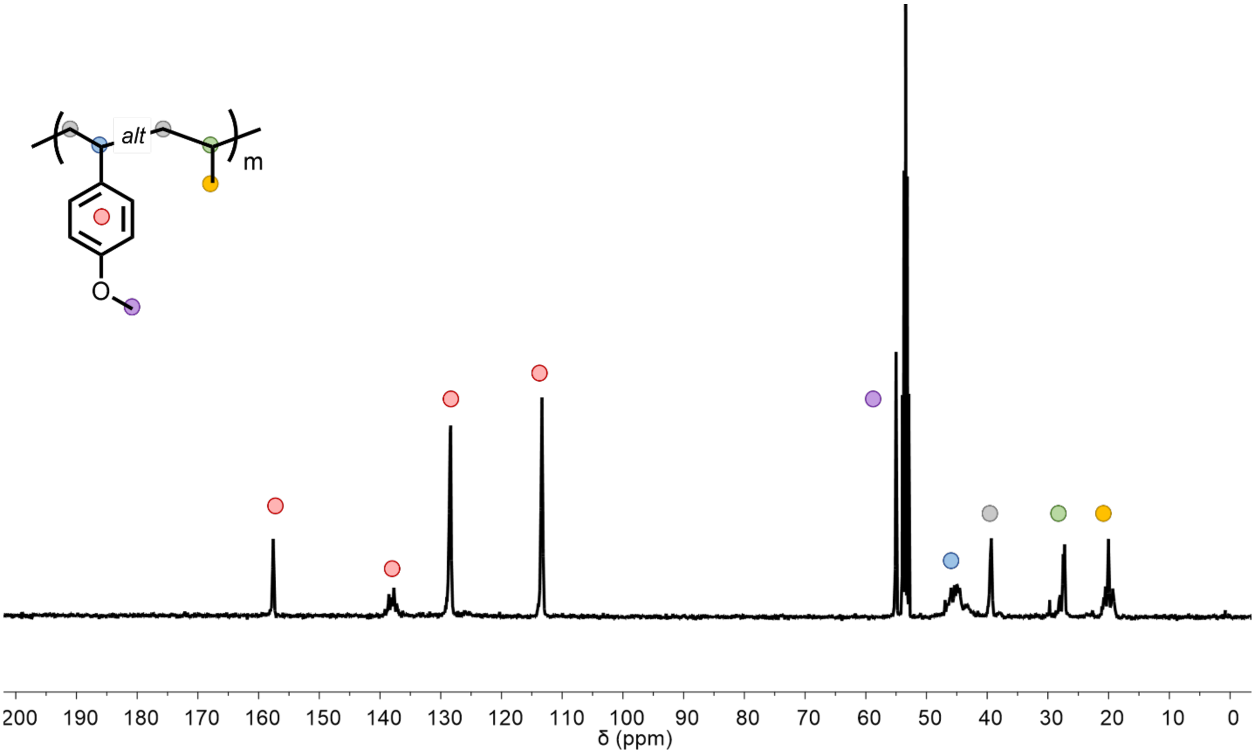

Figure S66. *^{13}C NMR spectrum of P(StyOMe-alt-P) in CD_2Cl_2.*

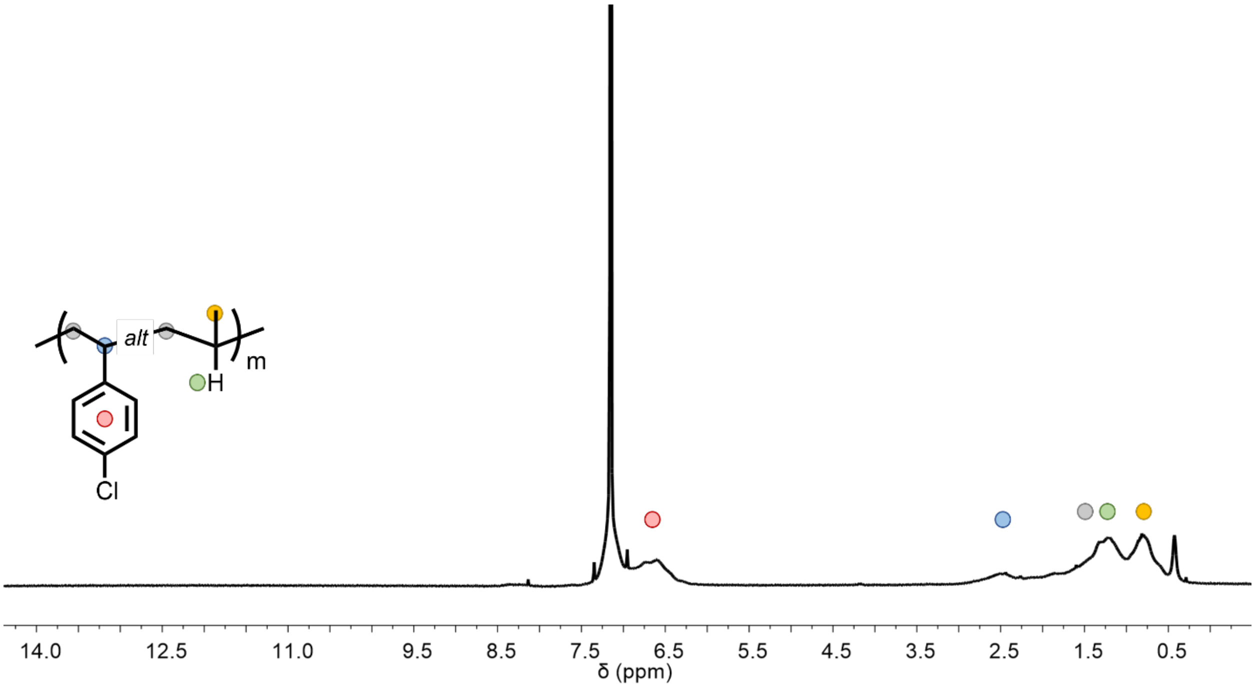

Figure S67. *^{1}H NMR spectrum of P(StyCl-alt-P) in benzene-d_6.*

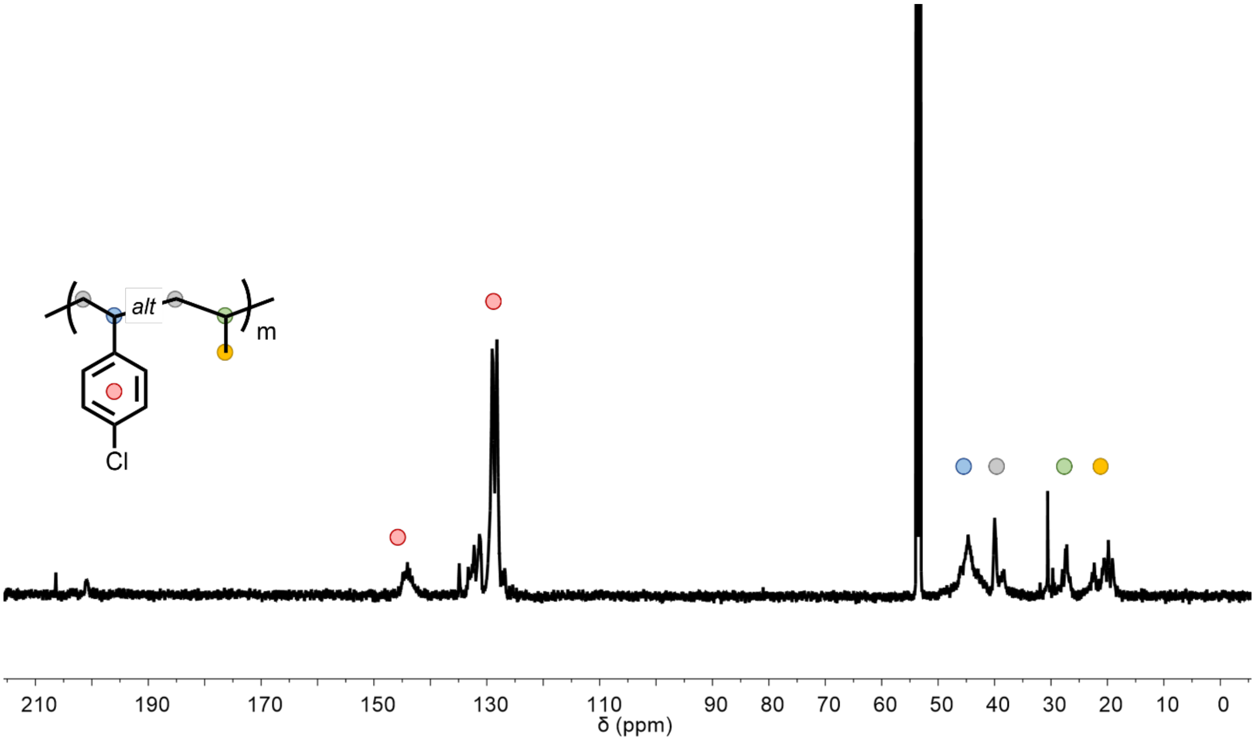

Figure S68. *^{13}C NMR spectrum of P(StyCl-alt-P) in CD_2Cl_2*

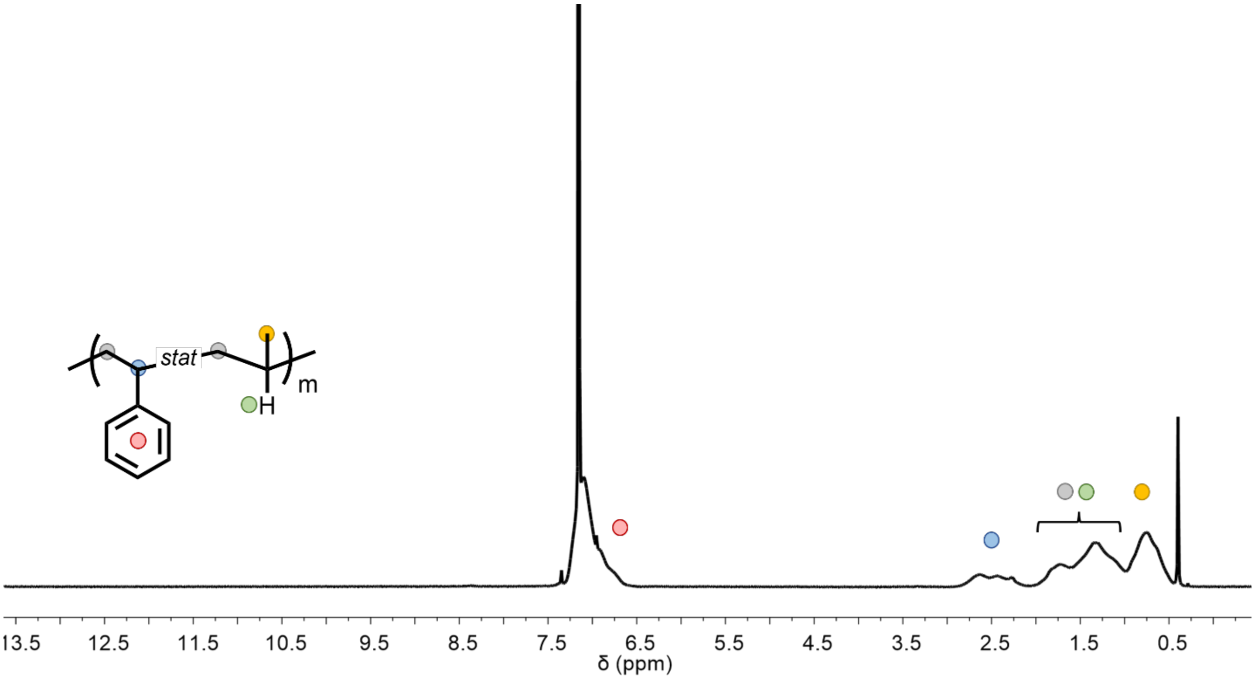

Figure S69. *^{1}H NMR spectrum of P($Sty_{0.54}$-stat-$P_{0.46}$) in benzene-d_6.*

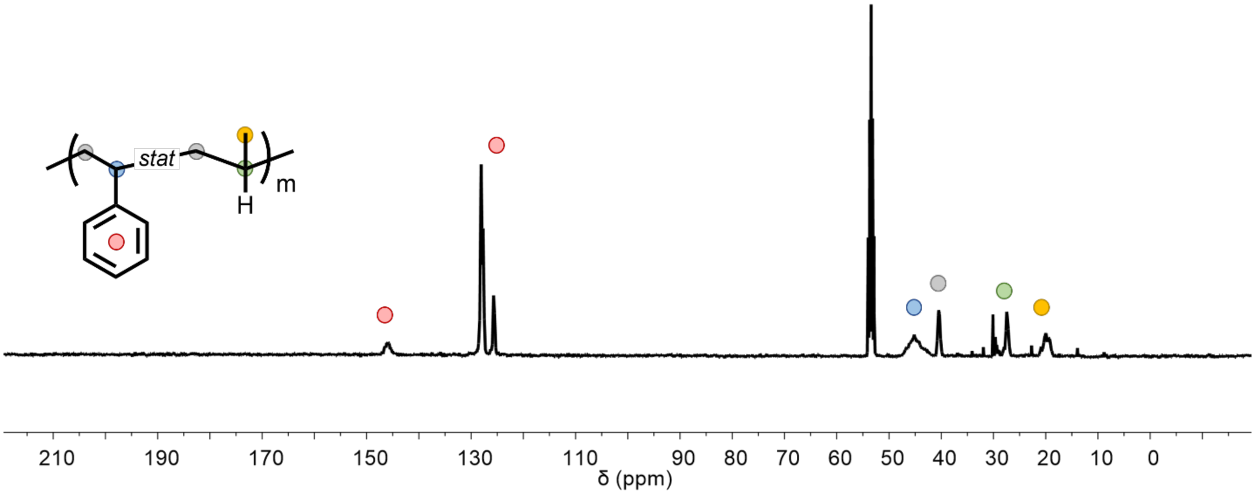

Figure S70. *^{13}C NMR spectrum of P(Sty$_{0.54}$-stat-P$_{0.46}$) in CD_2Cl_2.*

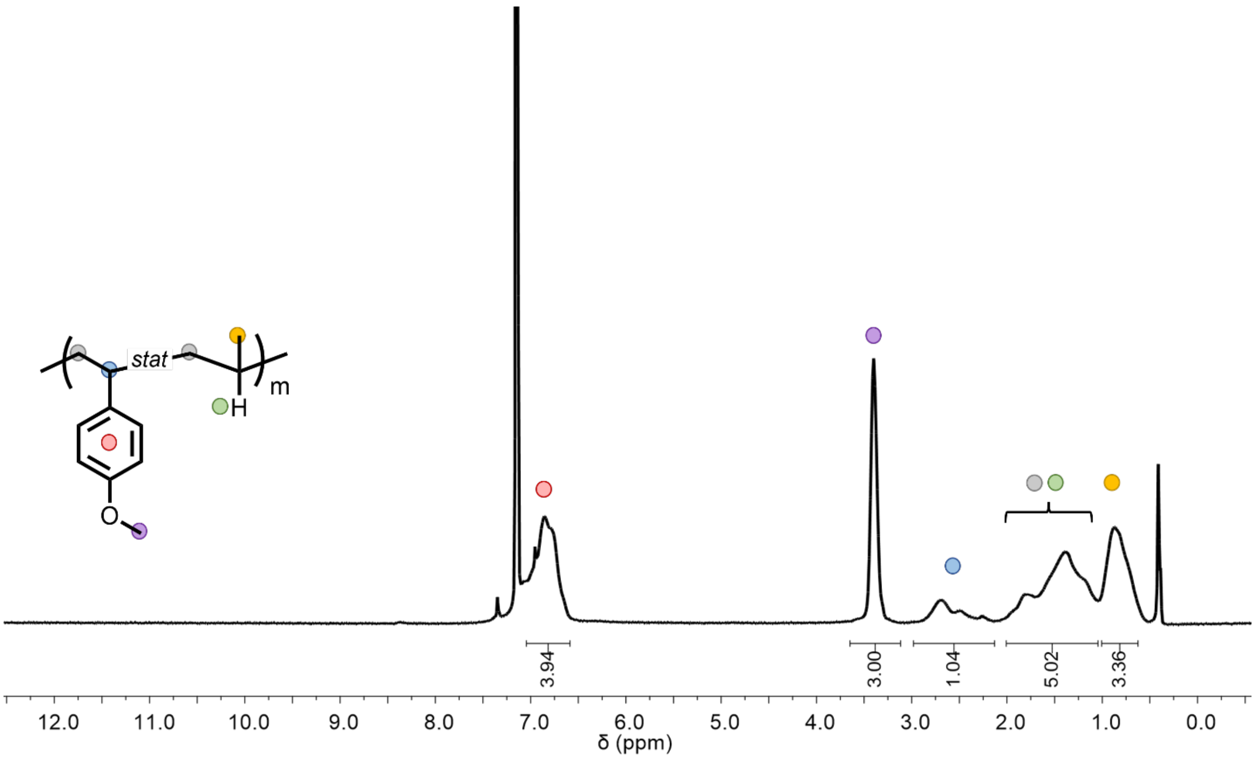

Figure S71. *^{1}H NMR spectrum of P(StyOMe$_{0.51}$-stat-P$_{0.49}$) in benzene-d_6.*

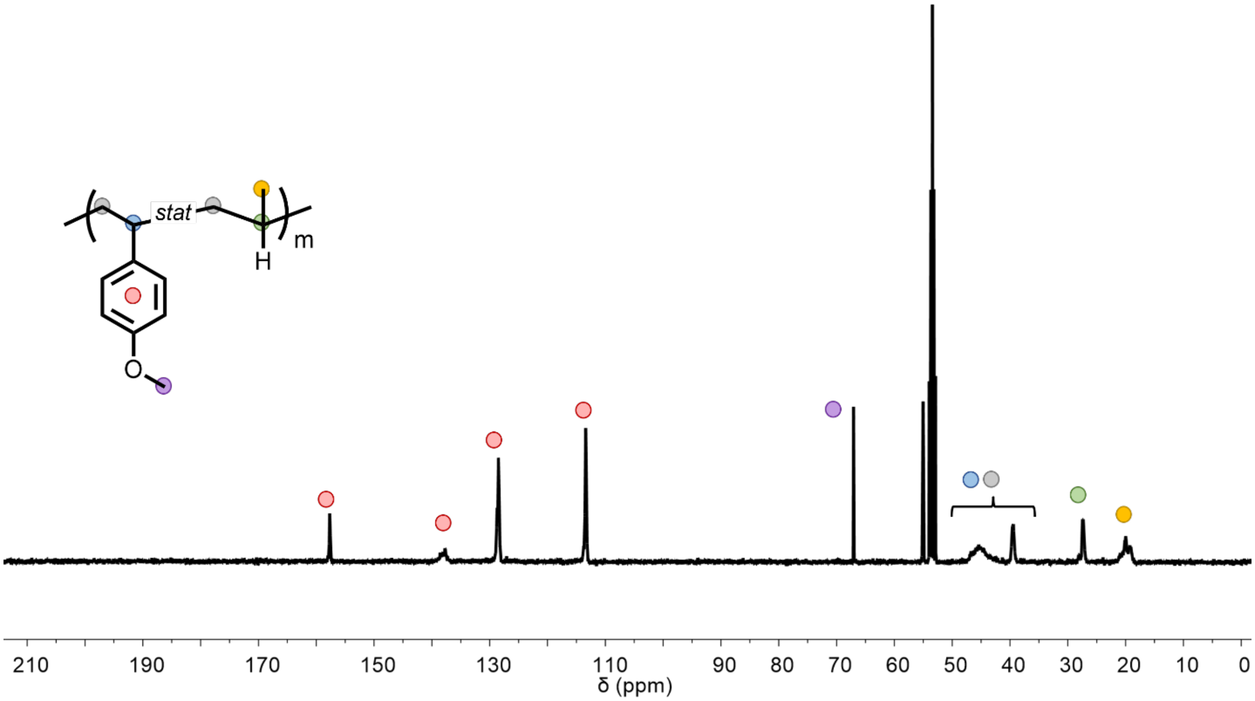

Figure S72. *^{13}C NMR spectrum of P(StyOMe$_{0.51}$-stat-P$_{0.49}$) in CD_2Cl_2.*

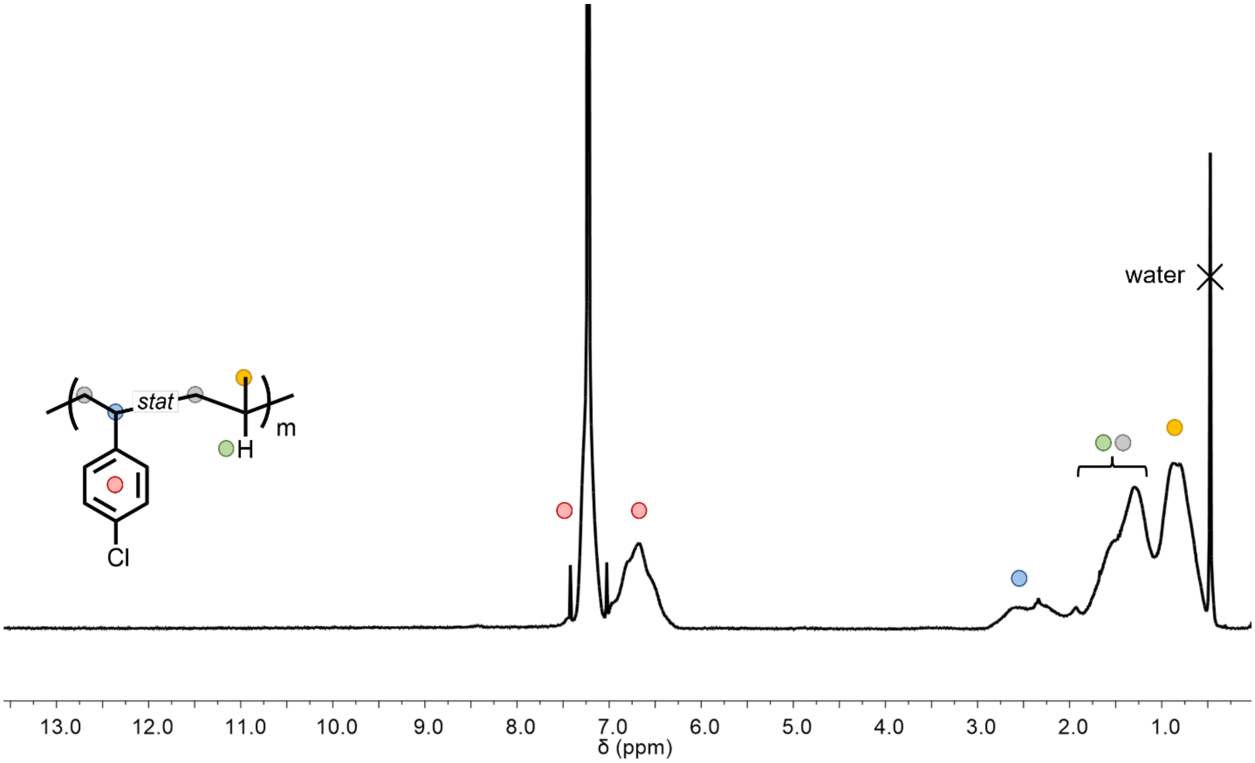

Figure S73. *^{1}H NMR spectrum of P(StyCl$_{0.47}$-stat-P$_{0.53}$) in benzene-d_6.*

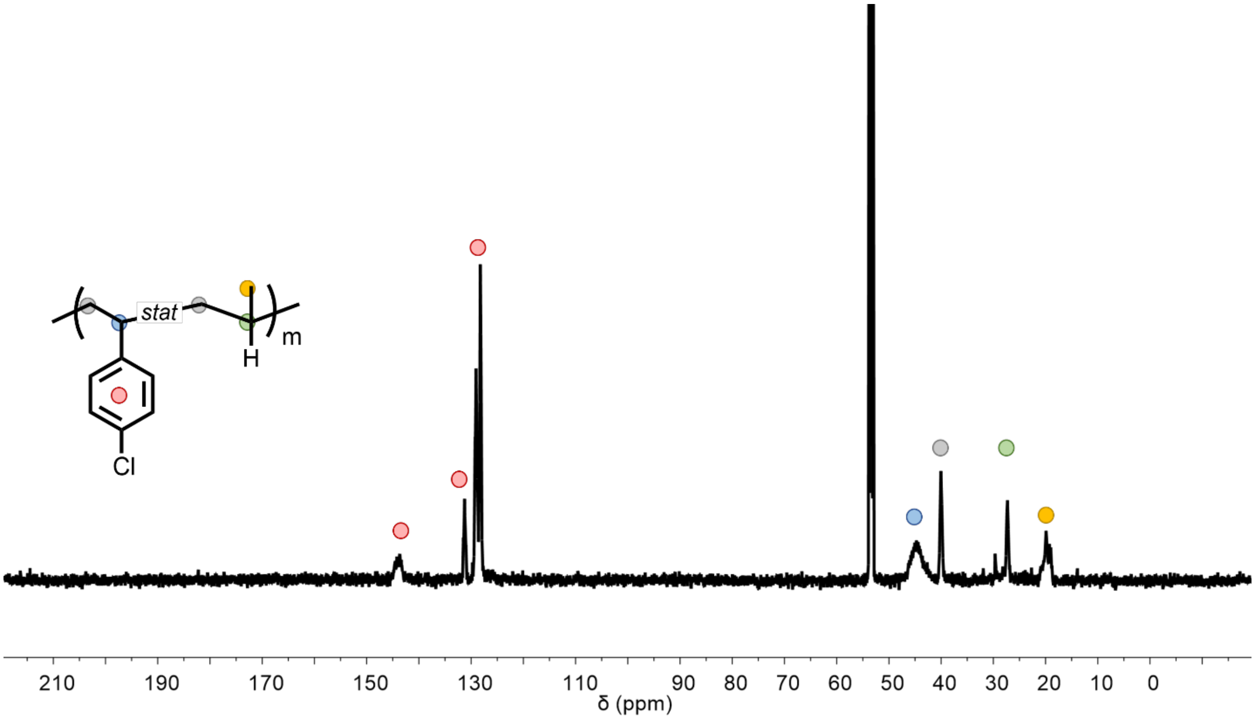

Figure S74. *^{13}C NMR spectrum of P(StyCl$_{0.47}$-stat-P$_{0.53}$) in CD$_2$Cl$_2$.*

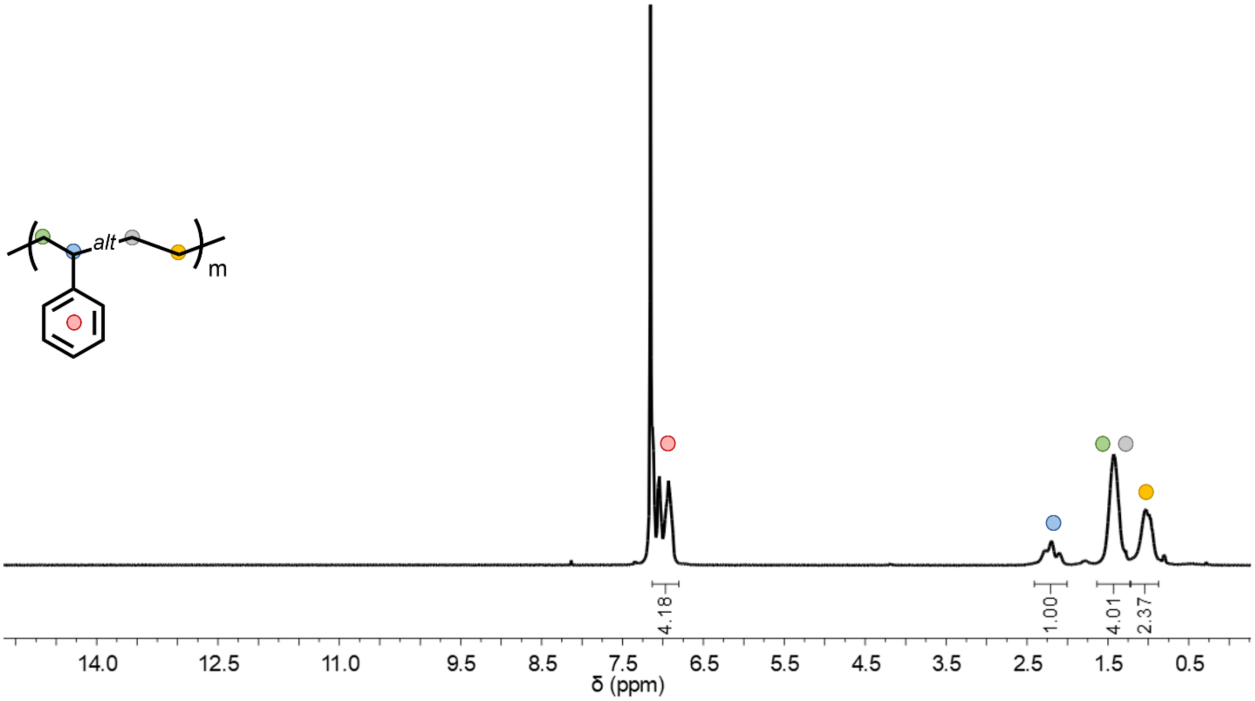

Figure S75. *^{1}H NMR spectrum of P(Sty-alt-E) in benzene-d$_6$.*

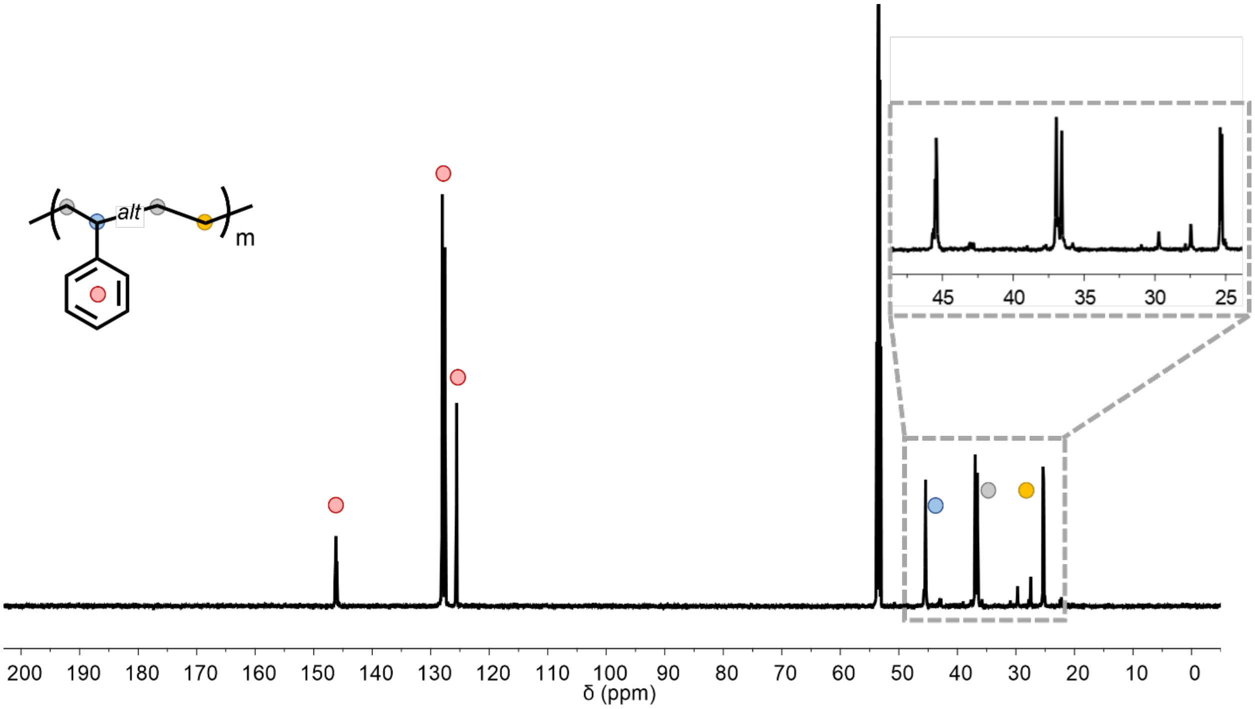

Figure S76. *^{13}C NMR spectrum of P(Sty-alt-E) in CD_2Cl_2.*

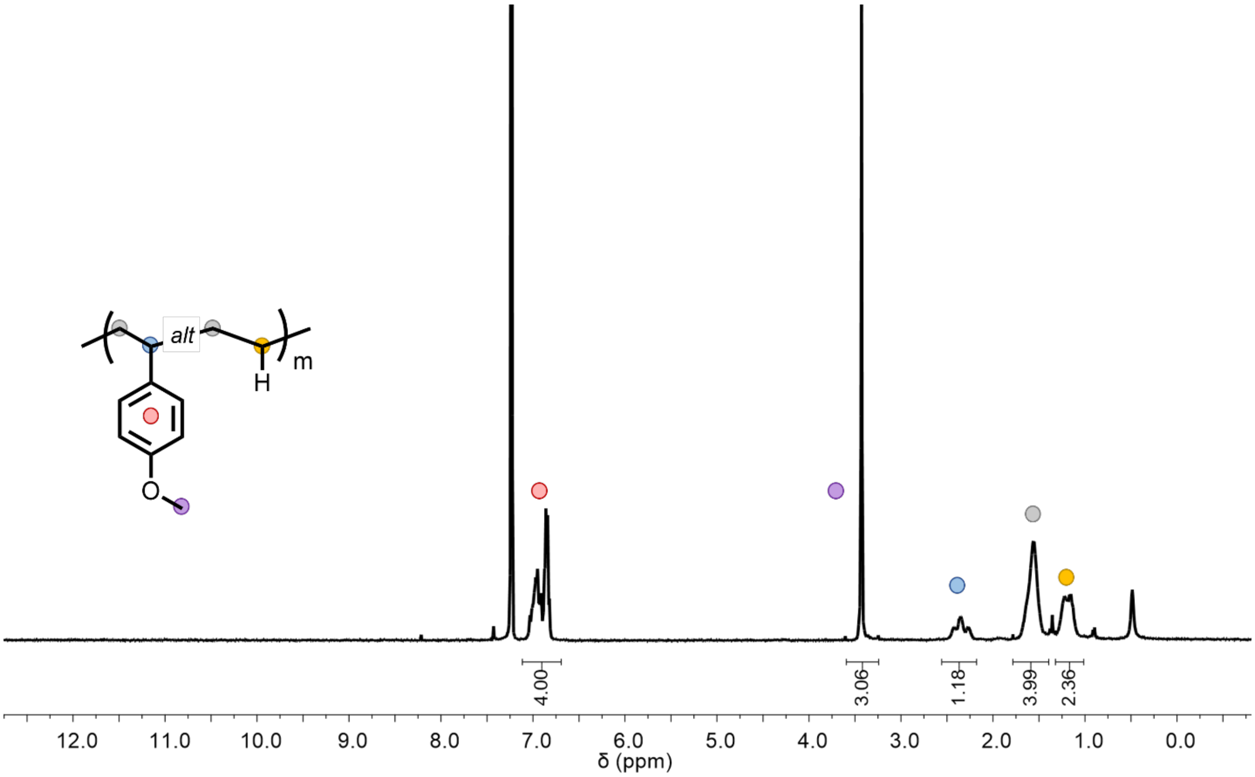

Figure S77. *^{1}H NMR spectrum of P(StyOMe-alt-E) in benzene-d_6.*

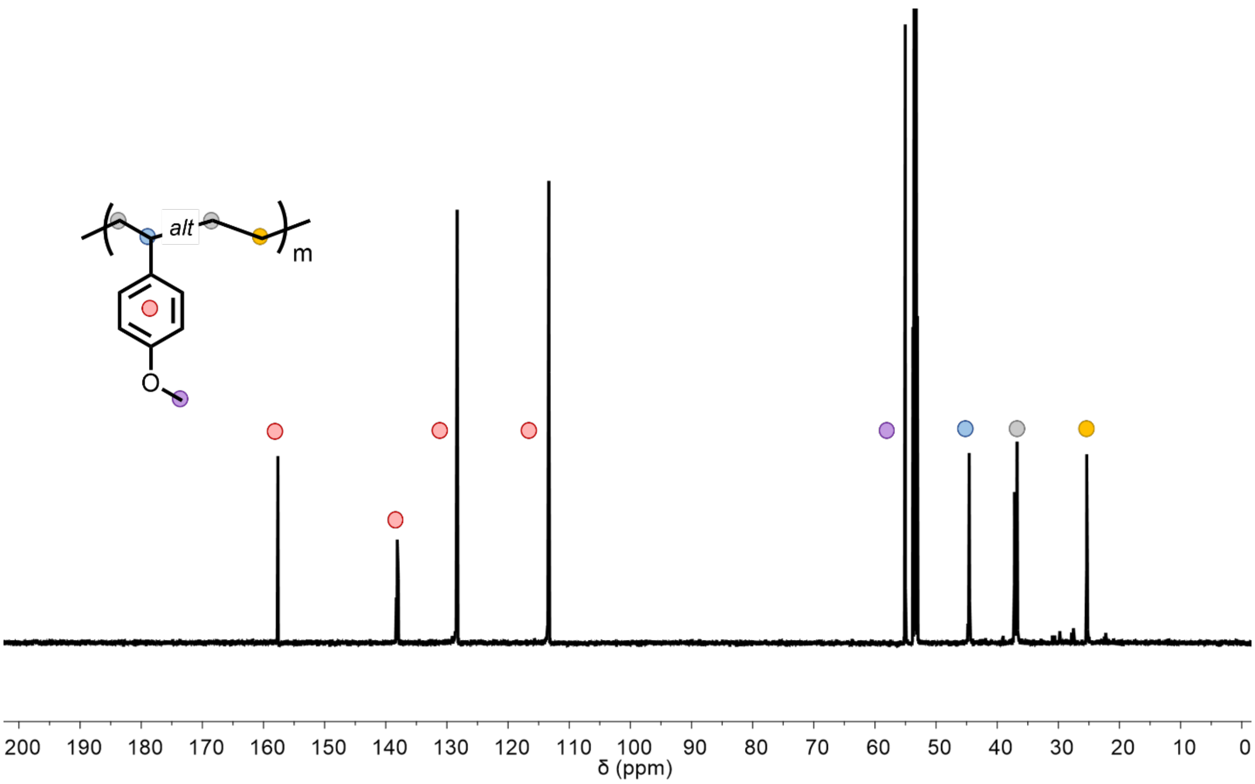

Figure S78. *^{13}C NMR spectrum of P(StyOMe-alt-E) in CD_2Cl_2.*

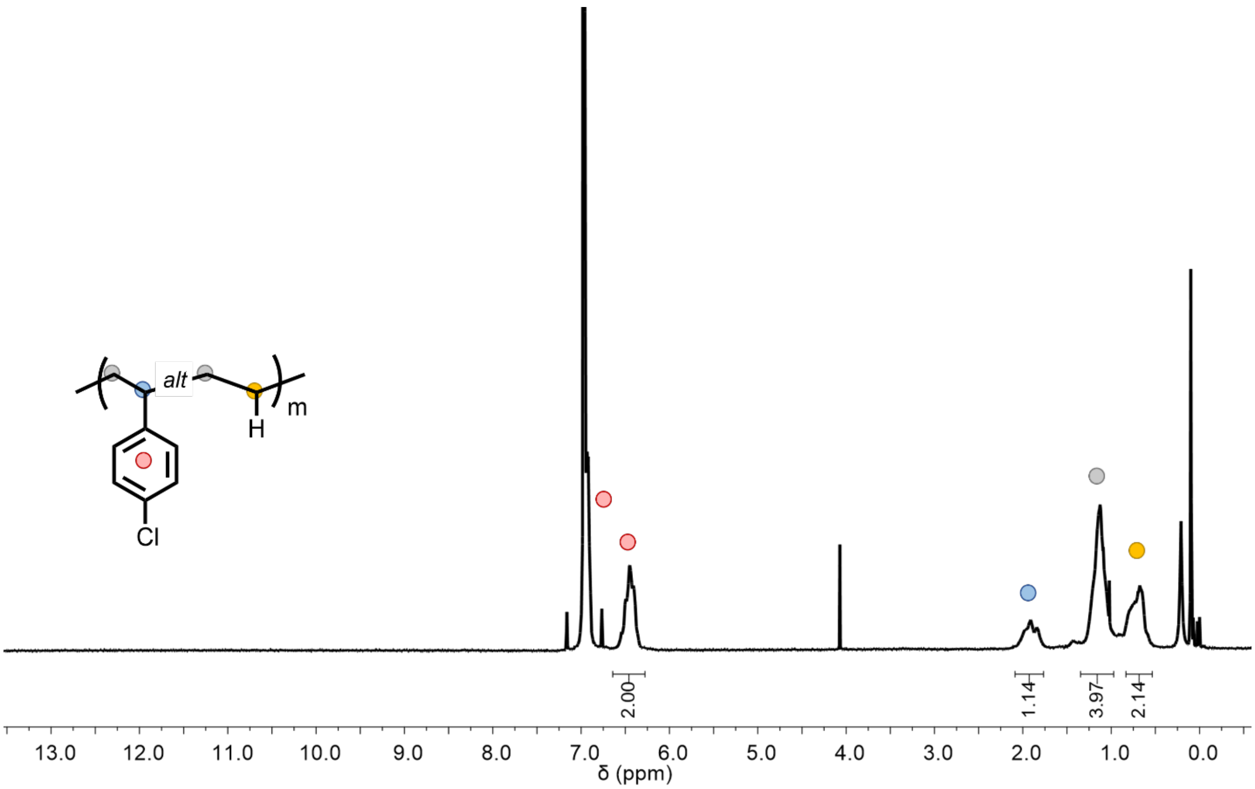

Figure S79. *^{1}H NMR spectrum of P(StyCl-alt-E) in benzene-d_6.*

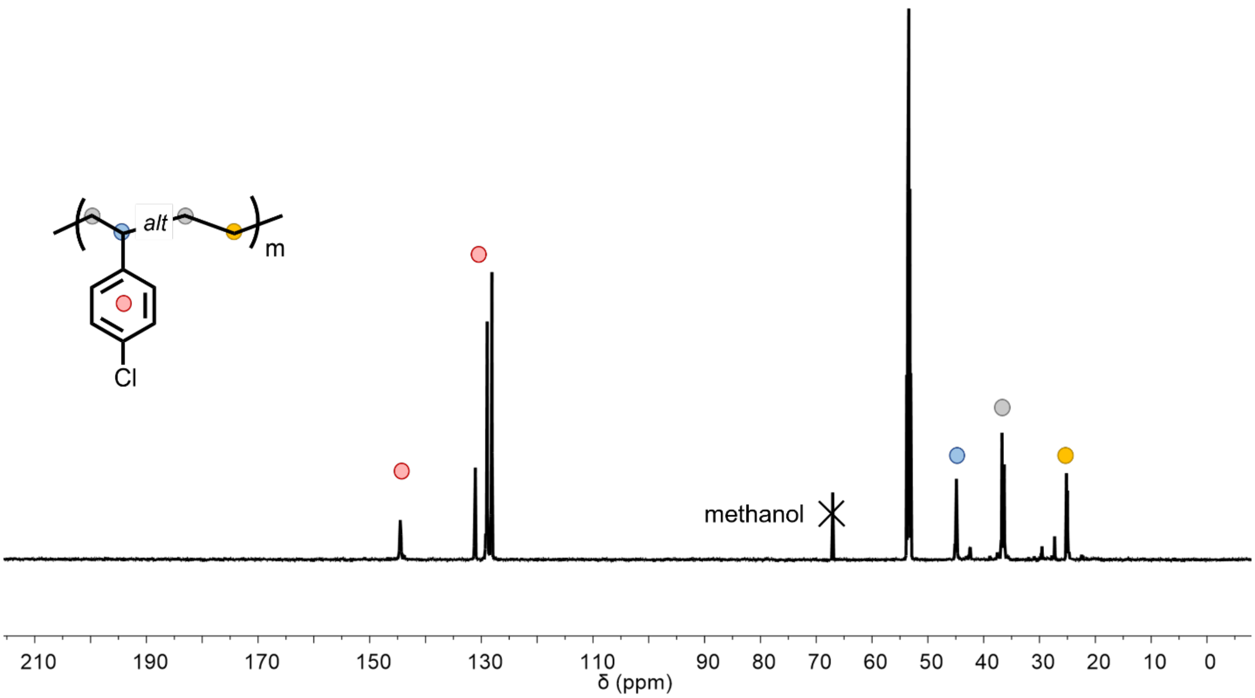

Figure S80. *^{13}C NMR spectrum of P(StyCl-alt-E) in CD_2Cl_2.*

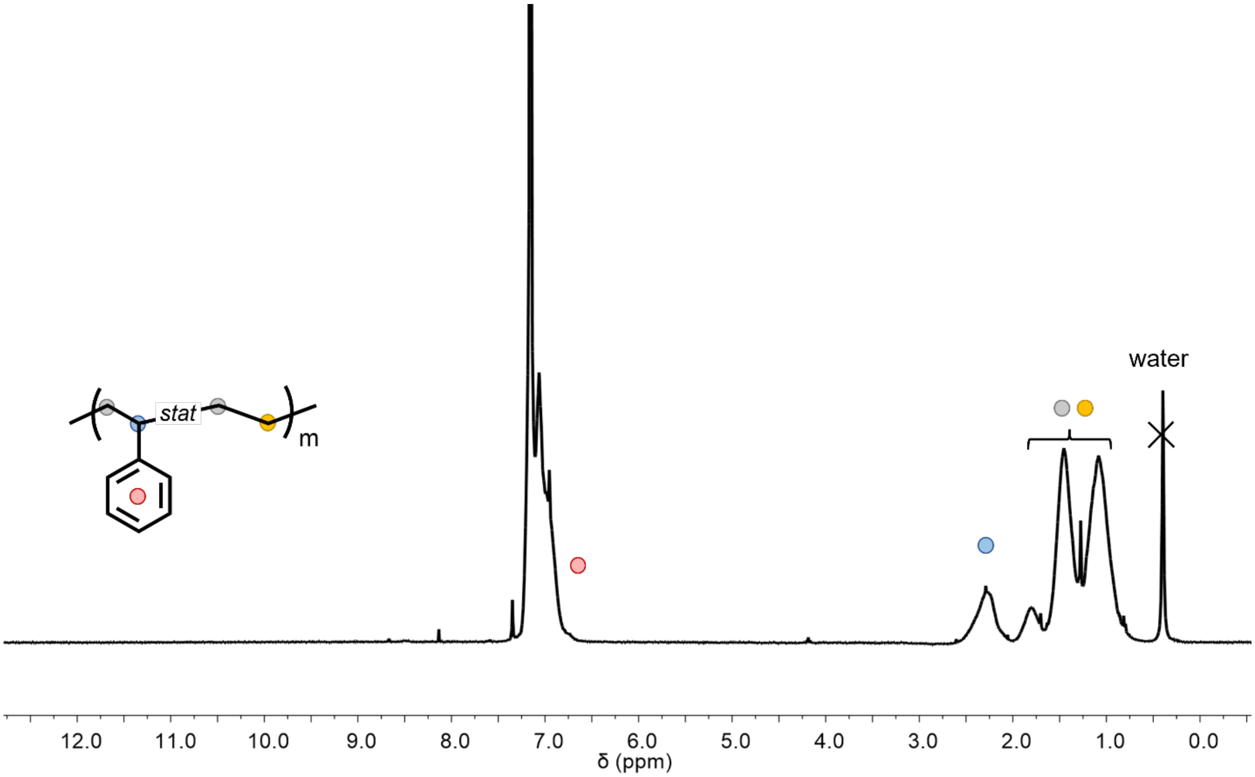

Figure S81. *^{1}H NMR spectrum of P($Sty_{0.47}$-stat-$E_{0.53}$) in benzene-d_6.*

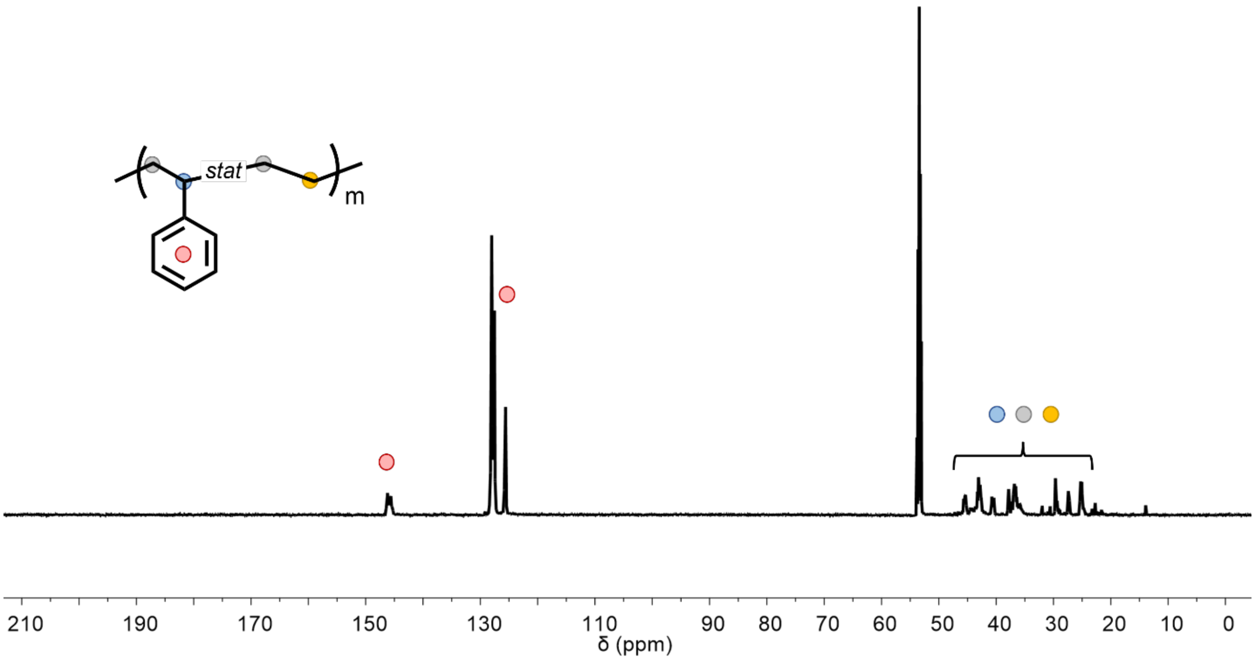

Figure S82. *^{13}C NMR spectrum of P(Sty$_{0.47}$-stat-E$_{0.53}$) in CD_2Cl_2.*

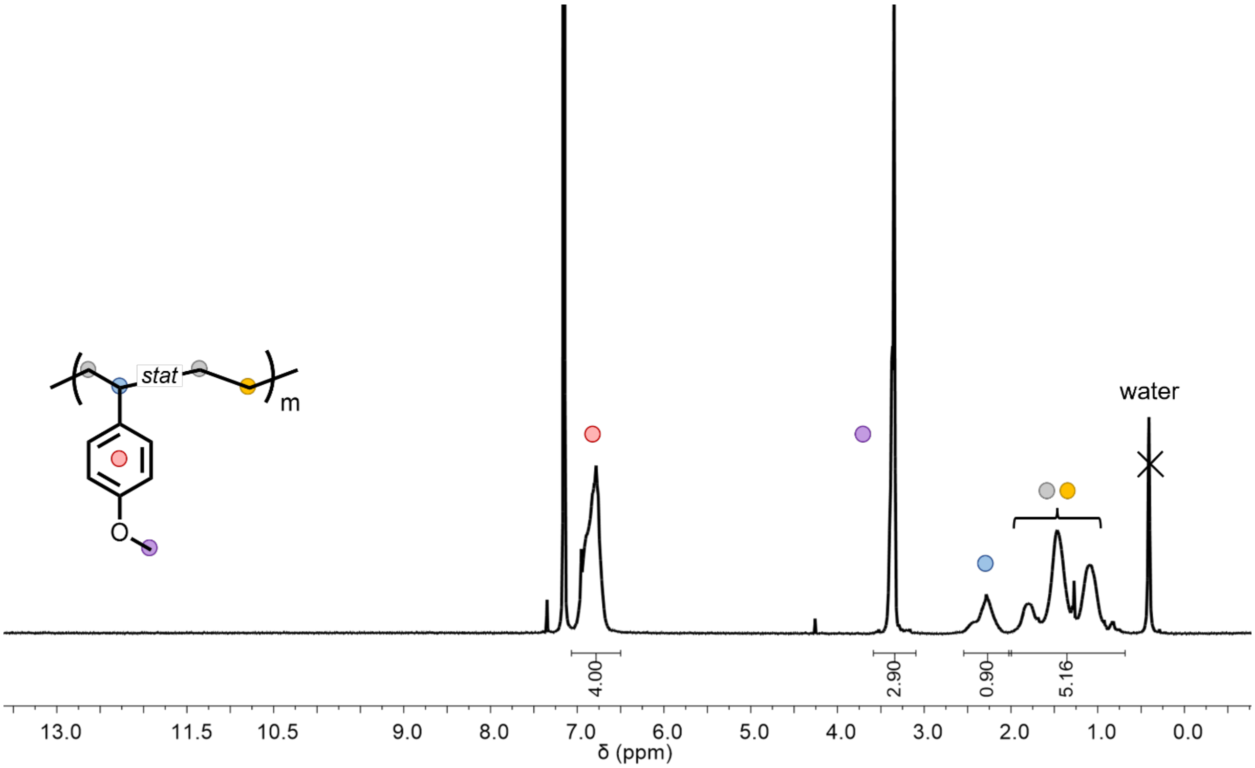

Figure S83. *^{1}H NMR spectrum of P(StyOMe$_{0.54}$-stat-E$_{0.46}$) in benzene-d_6.*

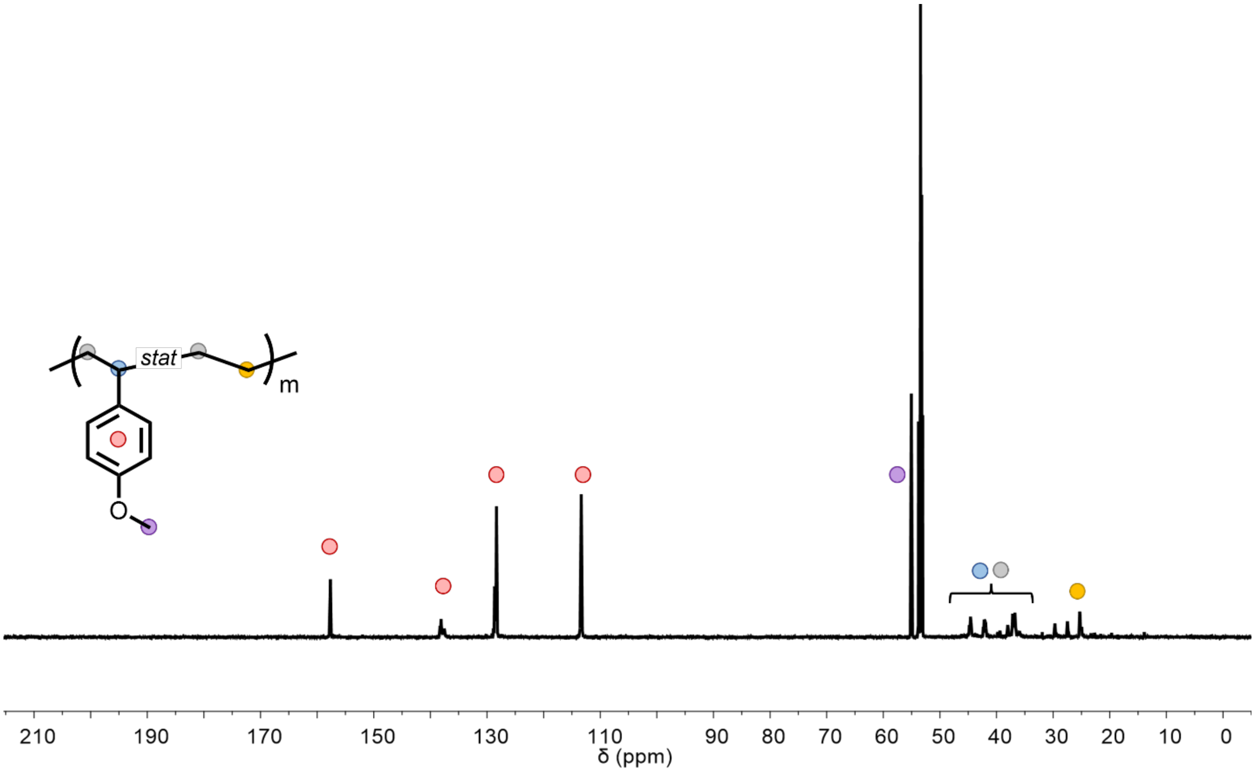

Figure S84. *^{13}C NMR spectrum of P(StyOMe$_{0.54}$-stat-E$_{0.46}$) in CD_2Cl_2.*

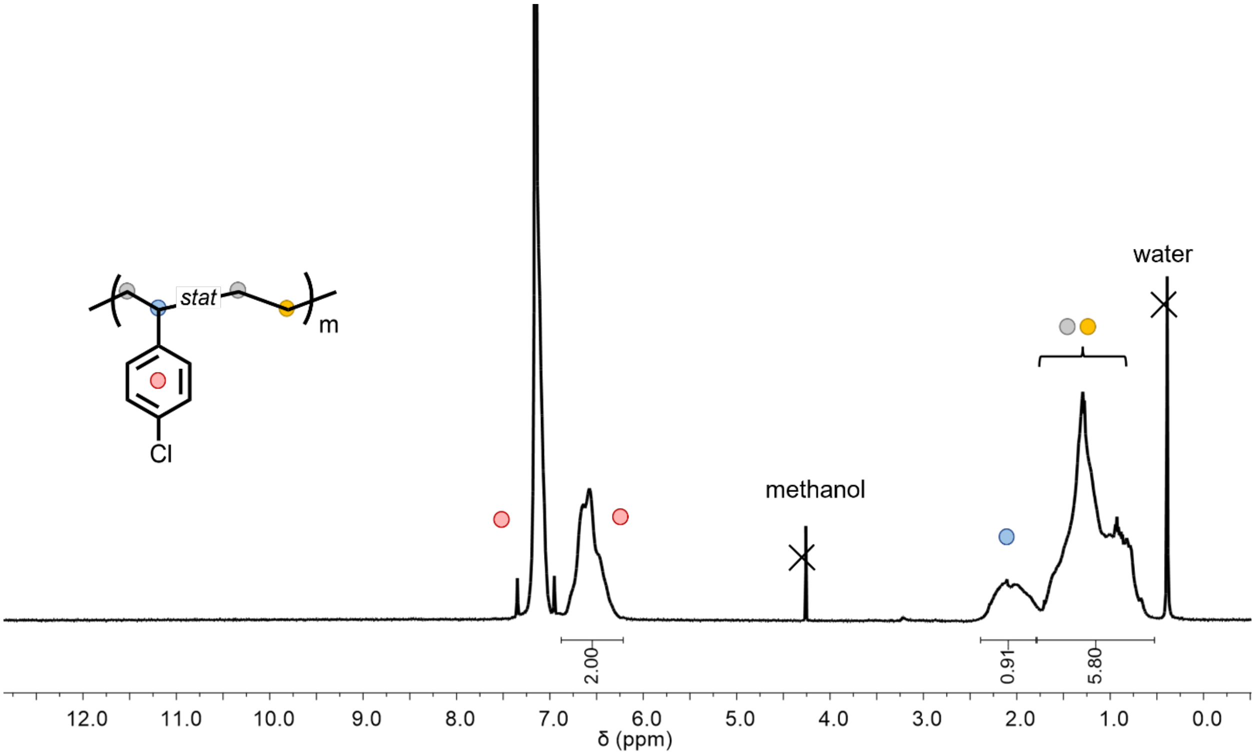

Figure S85. *^{1}H NMR spectrum of P(StyCl$_{0.49}$-stat-E$_{0.51}$) in benzene-d$_6$.*

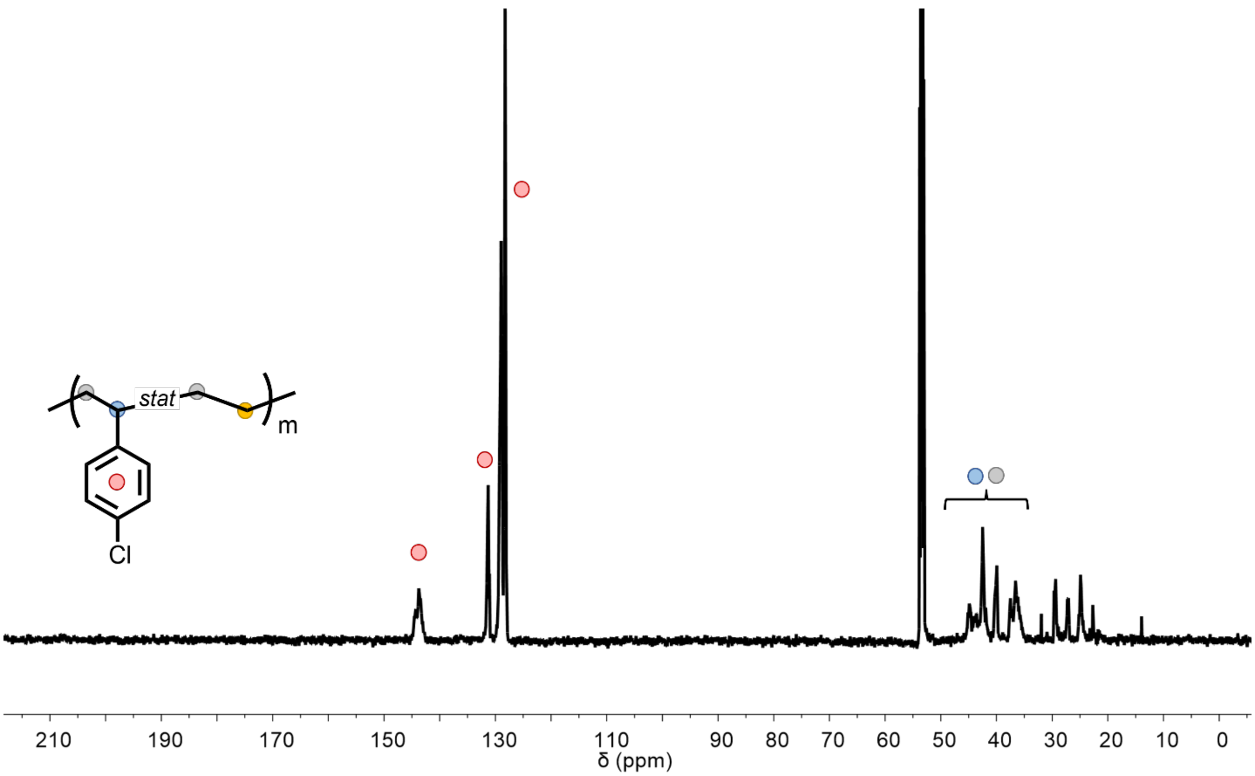

Figure S86. *^{13}C NMR spectrum of P(StyCl$_{0.49}$-stat-E$_{0.51}$) in CD_2Cl_2.*

3.4 DSC thermograms

The derivatives of heat flow were obtained by derivatization of the DSC thermograms. The derivative plots were smoothed to remove noise from signals and improve the readability of the graphs (Savitzky-Golay method, point of window = 50). The dash lines mark copolymer glass transition onsets and offsets.

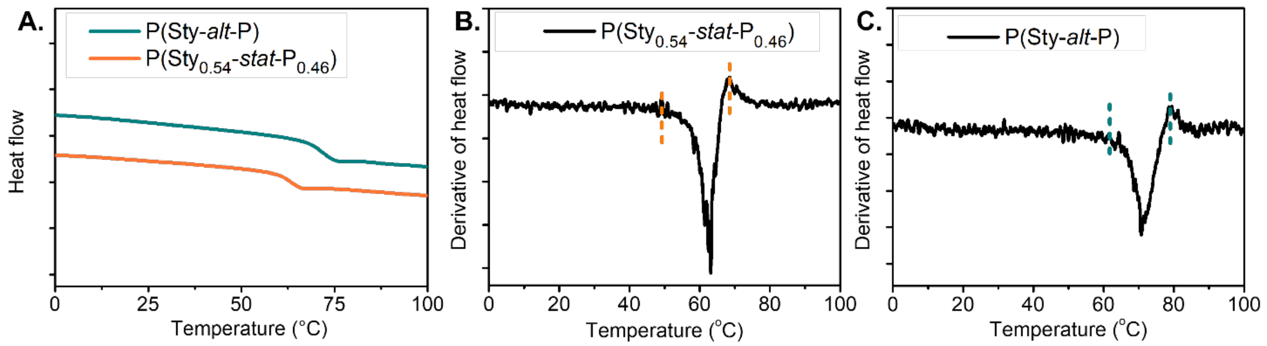

Figure S87. *A) Differential scanning calorimetry (DSC) of P(Sty-alt-P) and P($Sty_{0.54}$-stat-$P_{0.46}$). B) Derivative of heat flow of P(Sty-alt-P). C) Derivative of heat flow of P($Sty_{0.54}$-stat-$P_{0.46}$).*

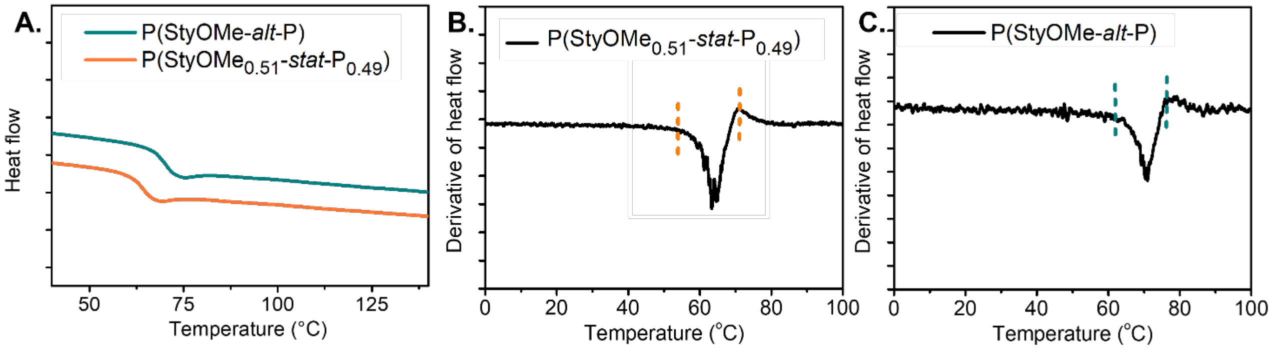

Figure S88. *A) DSC of P(StyOMe-alt-P) and P($StyOMe_{0.51}$-stat-$P_{0.49}$). B) Derivative of heat flow of P(StyOMe-alt-P). C) Derivative of heat flow of P($StyOMe_{0.51}$-stat-$P_{0.49}$).*

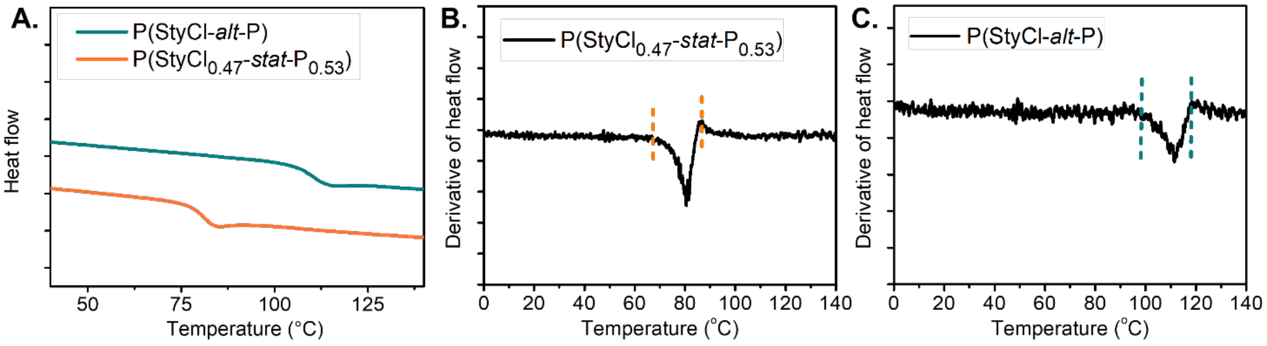

Figure S89. *A) DSC of P(StyCl-alt-P) and P(StyCl$_{0.47}$-stat-P$_{0.53}$). B) Derivative of heat flow of P(StyCl-alt-P). C) Derivative of heat flow of P(StyCl$_{0.47}$-stat-P$_{0.53}$).*

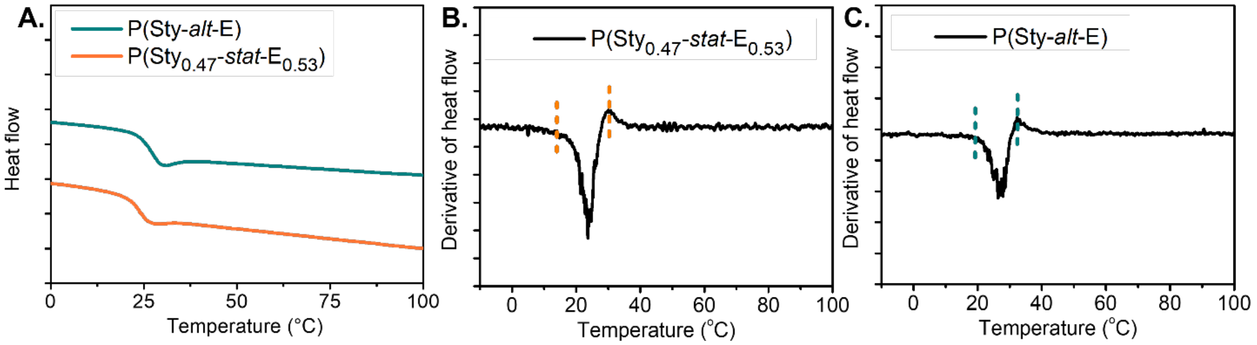

Figure S90. *A) DSC of P(Sty-alt-E) and P(Sty$_{0.47}$-stat-E$_{0.53}$). B) Derivative of heat flow of P(Sty-alt-E). C) Derivative of heat flow of P(Sty$_{0.47}$-stat-E$_{0.53}$).*

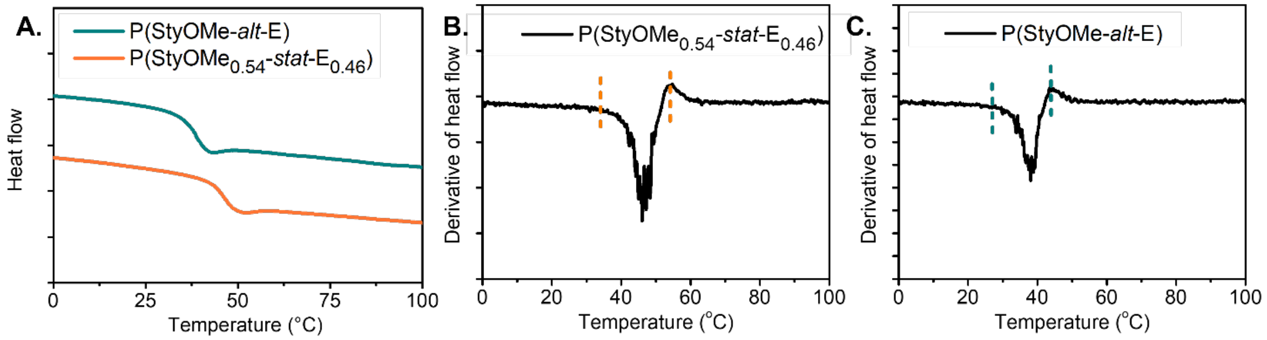

Figure S91. *A) DSC of P(StyOMe-alt-E) and P(StyOMe$_{0.54}$-stat-E$_{0.46}$). B) Derivative of heat flow of P(StyOMe-alt-E). C) Derivative of heat flow of P(StyOMe$_{0.54}$-stat-E$_{0.46}$).*

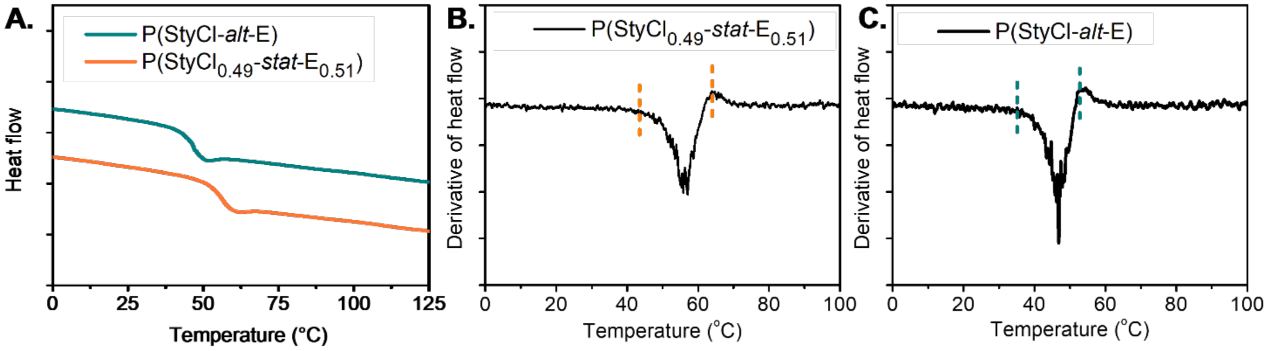

Figure S92. *A) DSC of P(StyCl-alt-E) and P(StyCl$_{0.49}$-stat-E$_{0.51}$). B) Derivative of heat flow of P(StyCl-alt-E). C) Derivative of heat flow of P(StyCl$_{0.49}$-stat-E$_{0.51}$).*

4. References

1. J. T. Lai, D. Filla and R. Shea, Functional Polymers from Novel Carboxyl-Terminated Trithiocarbonates as Highly Efficient RAFT Agents, *Macromolecules*, 2002, **35**, 6754-6756.
2. G. Moad, Y. K. Chong, A. Postma, E. Rizzardo and S. H. Thang, Advances in RAFT polymerization: the synthesis of polymers with defined end-groups, *Polymer*, 2005, **46**, 8458-8468.
3. Y. Kametani and M. Ouchi, Saccharin-pendant methacrylamide as a unique monomer in radical copolymerization: peculiar alternating copolymerization with styrene, *Polym. Chem.*, 2020, **11**, 6505-6511.
4. A. C. Benniston, A. Harriman, P. Y. Li, J. P. Rostron, H. J. van Ramesdonk, M. M. Groeneveld, H. Zhang and J. W. Verhoeven, Charge Shift and Triplet State Formation in the 9-Mesityl-10-methylacridinium Cation, *J. Am. Chem. Soc.*, 2005, **127**, 16054-16064.
5. V. T. Nguyen, V. D. Nguyen, G. C. Haug, H. T. Dang, S. F. Jin, Z. L. Li, C. Flores-Hansen, B. S. Benavides, H. D. Arman and O. V. Larionov, Alkene Synthesis by Photocatalytic Chemoenzymatically Compatible Dehydrodecarboxylation of Carboxylic Acids and Biomass, *ACS Catal.*, 2019, **9**, 9485-9498.
6. A. R. White, L. Wang and D. A. Nicewicz, Synthesis and Characterization of Acridinium Dyes for Photoredox Catalysis, *Synlett.*, 2019, **30**, 827-832.
7. Y. Kametani and M. Ouchi, One-Pot Preparation of Methacrylate/Styrene Alternating Copolymers via Radical Copolymerization and Alcoholysis Modification: Sequence Impacts on Glass Transition Temperature, *ACS Polym. Au*, 2021, **1**, 10-15.
8. A. Adili, A. B. Korpusik, D. Seidel and B. S. Sumerlin, Photocatalytic Direct Decarboxylation of Carboxylic Acids to Derivatize or Degrade Polymers, *Angew. Chem. Int. Ed.*, 2022, **61**, e202209085.
